# Supplementary material for: Thermodynamic modeling of RsmA - mRNA interactions capture novel direct binding across the Pseudomonas aeruginosa transcriptome
Source: Front Mol Biosci. 2025 Feb 20;12:1493891. doi: 10.3389/fmolb.2025.1493891 (PMC11882435; doi:10.3389/fmolb.2025.1493891)
Supplement: Supplementary file 4 [file DataSheet1.pdf]

Supplemental Figures

- **Figure 1- Comparison of prior model with that described in this work**
- **Figure 2- Extended description of peak calling method**
- **Figure 3 – Predicted effects of binding on translation**
- **Figure 4 – Validation of binding site predictions using previously footprinted mRNAs from closely related organisms**
- **Figure 5 – Summary of RNA Co-Immunoprecipitation Sequencing and Proteomics Results**
- **Figure 6 - RsmA EMSA results for *rhlR, rhlI, aprD,* and *aprX***
- **Figure 7 – UMAP clustering of aggregate sequencing data**
- **Figure 8 – Filter binding images for *rsaL* binding site mutations**
- **Figure 9 - Filter binding images for *mvaT* binding site mutations**

Supplementary Figure 1: Method additions to current model

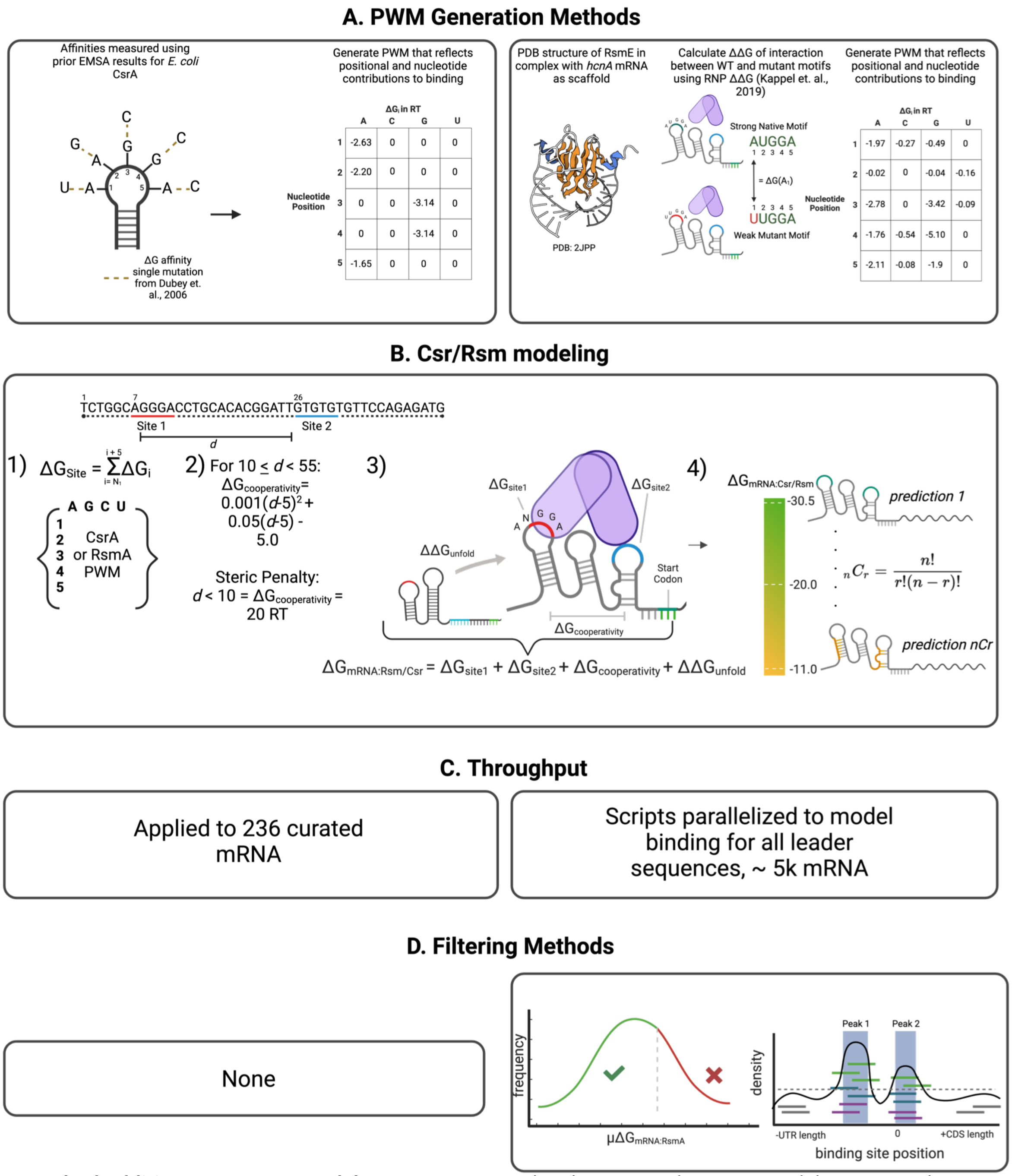

Figure 1: Method additions to current model Here we present the changes made to our modeling approach to customize and broader the protocol for capturing interactions between RsmA and mRNA targets in *P. aeruginosa*. The left side represents the procedure from our prior published model and the right represents additional alterations made to customize the approach for *P. aeruginosa*. (A) The method used for generating the position weight matrices (PWMs) differ between the two versions of the model. the positional energies were interpreted from prior mutational binding assays between CsrA and individually mutated mRNAs (Dubey et al., 2006). The method described in this publication generated an Rsm-family specific PWM using the Rosetta modeling RNP ΔΔG tool, which allowed for the generation of a new PWM using a computational mutagenesis screen. (B) Overview of the modeling parameters defined in Lesitra et. al., 2018 and used in this study. 1) Either CsrA (Leistra et. al., 2018) or RsmA (this work) PWMs are used to calculate the ΔG_{site} term, which is the sum of the individual nucleotide contributions to overall affinity. 2) The distance between binding sites informs the $\Delta G_{cooperativity}$ term, imposing a penalty to predictions that are less than 10 or more than 55 nt apart. 3) The change in minimum free energy from the unbound to the bound state is captured using RNAfold, and is included as the $\Delta\Delta G_{unfold}$ term in the model. For each pair of potential sites in the entire possible window, the $\Delta G_{mRNA:Rsm/Csr}$ is calculated and then 4) sorted from most to least probable based on the $\Delta G_{mRNA:Rsm/Csr.}$ (C) The current model script was updated for parallel computing on the Stampede2 compute cluster, allowing for predictions to be generated on an order of magnitude more genes than originally performed. (D) Our model uses prior experimental results that inform filtering terms such as a cutoff of the mean $\Delta G_{mRNA:RsmA}$ term and peak calling for identification of specific binding sites on the transcript. This differs from the prior model in that no cutoff metrics were defined to differentiate bound from unbound genes.

Supplementary Figure 2: Peak calling method

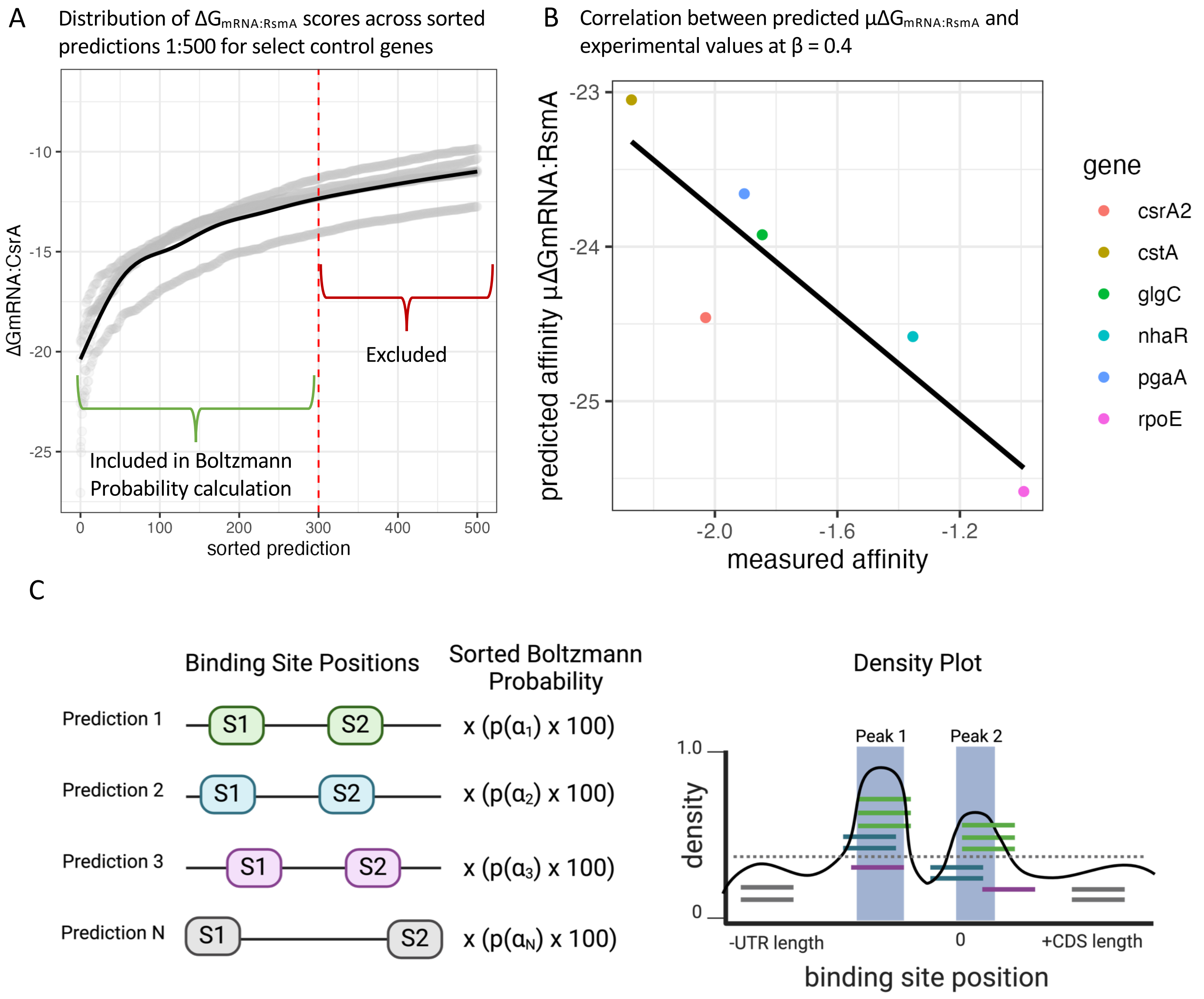

Figure 2: Transformation of model predicted energy to generate the $\mu\Delta G_{mRNA:RsmA}$ and perform peak calling. A) Affinity scores for the top 500 sorted predictions for a selection of positive control genes from [30] reveals that the $\Delta G_{mRNA:CsrA}$ for these predictions plateaus surrounding prediction 300. Predictions that fall beyond 300 are excluded from the overall Boltzmann probability calculation. B) Calculating the appropriate $\mu\Delta G_{mRNA:RsmA}$ requires an appropriate scaling factor β to weigh each prediction by the Boltzmann probability p(α). This plot shows the highest correlation between experimentally derived kD values (transformed into free energy) and predicted affinities occurs at β = 0.4 C) Visual depiction of how the binding site positions are weighed given the probability of each conformation occurring within the ensemble. The occurrence of each prediction is then multiplied by the probability of its conformation in the ensemble, and peaks are defined given the height of predictions at a given location along the span of the modeled sequence space. Peaks (blue boxes) are called given parameters defined in Methods section 2.4. The grey line reflects a minimum threshold that must be reached to be considered a peak, which we set as the maximum peak height calculated for the *lolB* transcript.

Supplemental Figure 3: Predicted effects of binding on translation

A

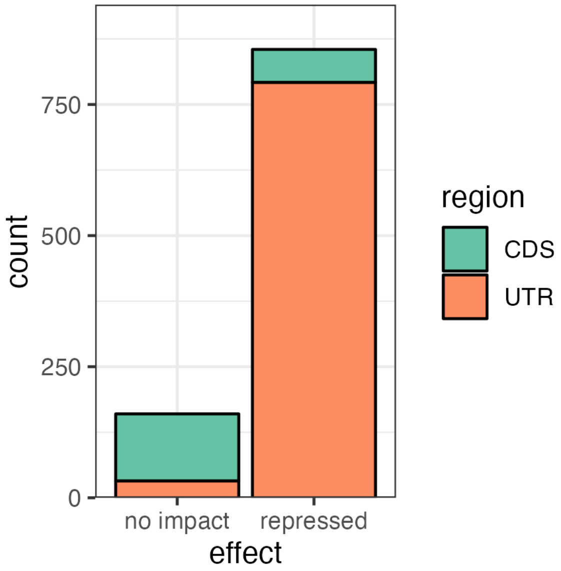

Figure 3: RsmA effects on translation across the transcriptome. The location of the binding sites influenced the predicted outcome of binding on translation using the OSTIR translation rate prediction tool. The majority of targets that passed energetic and peak filtering were predicted to be repressed upon binding by RsmA. The minority of these repressed outcomes are due to predictions present in the coding sequence (CDS) as opposed to the untranslated region (UTR). The majority of targets for which no regulatory outcome was predicted due to binding has predicted binding sites in the CDS relative to the UTR.

Supplemental Figure 4: Validation of model predictions using previously characterized binding sites

A ***Escherichia coli* CsrA-*glgC***

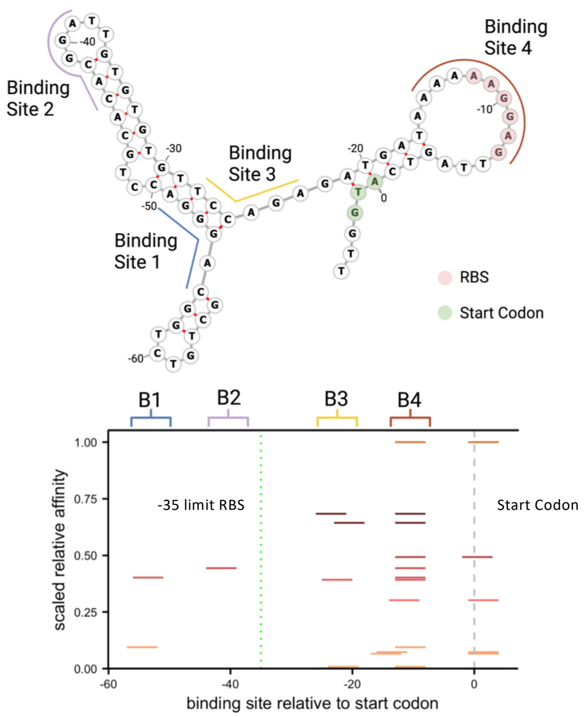

C ***Pseudomonas aeruginosa* RsmA-*tssA1***

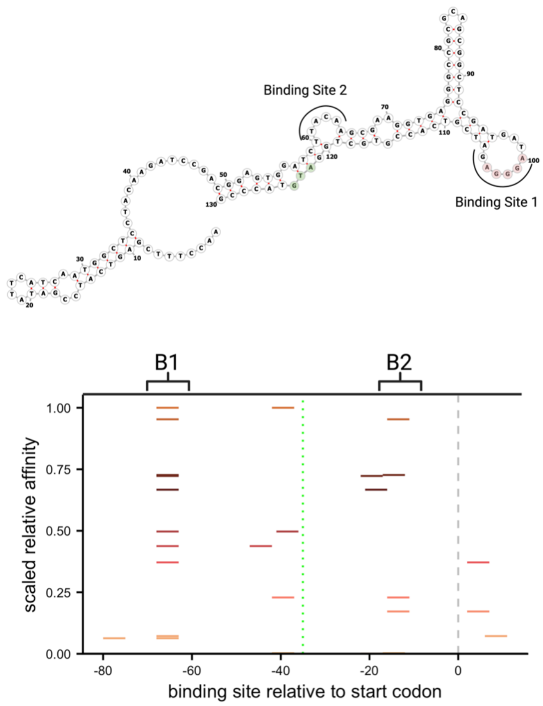

B ***Pseudomonas protegens* RsmE-*hcnA***

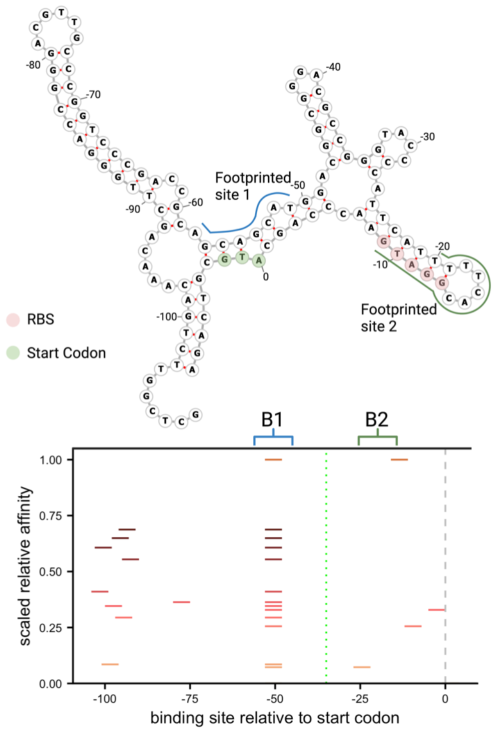

D **Binding peak frequencies across all filtered genes**

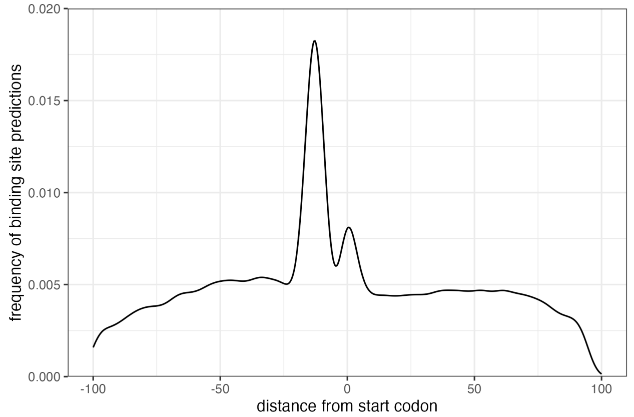

Figure 4: Applying our biophysical model to previously characterized Rsm-UTR interactions across three organisms validated broad applicability across Rsm/Csr encoding bacteria

A. 4 sites on the *glgC* UTR were previously found to be bound by CsrA in *E. coli* (Mercante et. al., 2009)**.** Applying our CsrA based model to this target correctly captured each binding site, witch a positive predictive value PPV of 0.77. **B.** Similarly as in A, we applied the same model to the *hcnA* UTR present in *P. protegens.* Binding sites of the CsrA homolog RsmE on the *hcnA* UTR were captured in (Lapouge et. al., 2007). Top predicted conformations centered around footprinted site 1. **C.** Within *P. aeruginosa*, the *tssA1* UTR is a well characterized target of the RsmA protein and two putative binding sites characterized in (Schulmeyer et. al., 2016) were accurately predicted by our model. **D.** Density plot of all binding peak frequencies plotted for all genes that pass the energetic and binding peak calling filters.

Supplemental Figure 5: RNA Co-Immunoprecipitation Sequencing and Proteomics Results

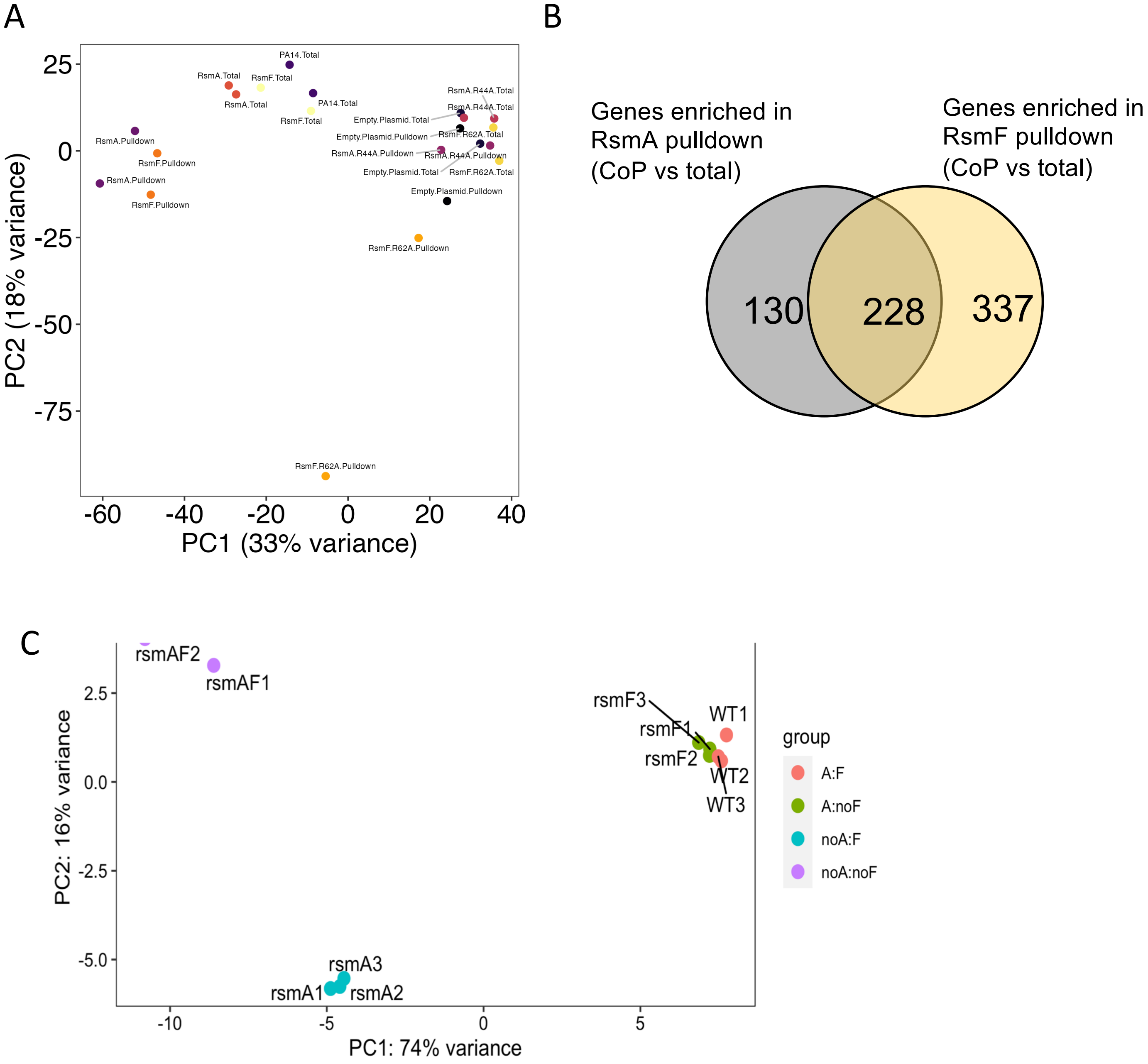

Figure 5: PCA analysis of counts data for RNA co-immunoprecipitation sequencing and proteomics on PA14 and PA103 respectively. A) PCA plot of RNA Co-Immunoprecipitation Sequencing read counts for pulled down and total RNA fractions. B) Venn diagram of exclusive and overlapping RNAs pulled down by either his-tagged RsmA or RsmF. C) PCA of protein counts in WT, delA, delF, and delAF PA103

Supplemental Figure 6: *rhlR, rhlI, aprD* and *aprX* EMSA results

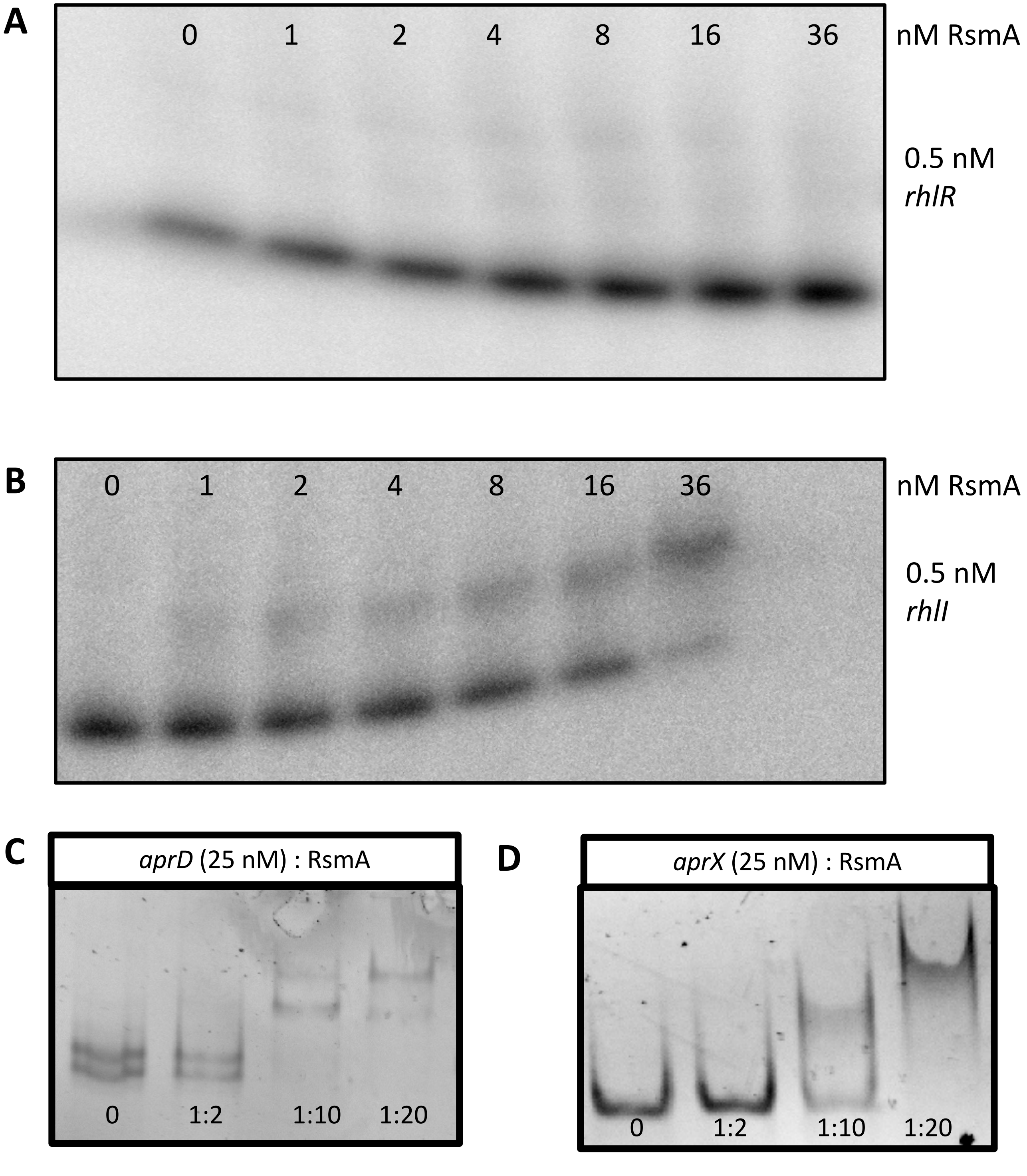

Figure 6: Electrophoretic Mobility Shift Assay to assess binding of RsmA to the 5’ leader sequence and first 100 bases of A) *rhlR* (no binding observed) and B) *rhlI* (kD = 20.88 $\underline{+}$ 7.5 nM). Un-radiolabeled EMSA was performed between the 5’ UTR sequence and first 100 bases of C) *aprD* and D) *aprX* RNAs

Supplemental Figure 7: UMAP clustering of sequencing data aggregated in Rajput et. al., 2022

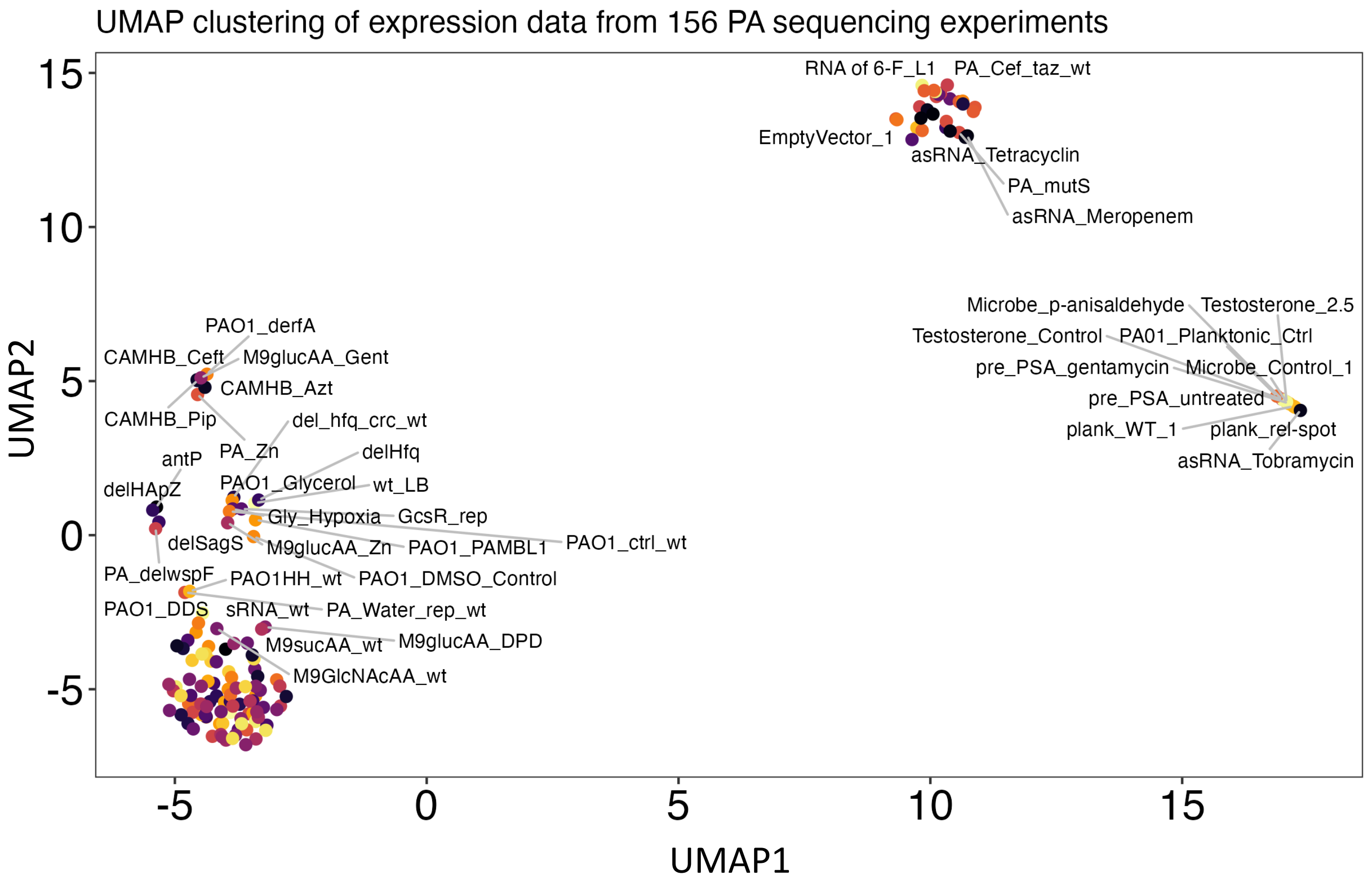

Figure 7: Uniform Manifold Approximation and Projection (UMAP) clustering expression data from 156 *Pseudomonas aeruginosa* sequencing experiments aggregated in Rajput et. al., 2022. Each data point is colored by experimental condition.

Supplemental Figure 8: Filter-binding images for *rsaL* binding mutants

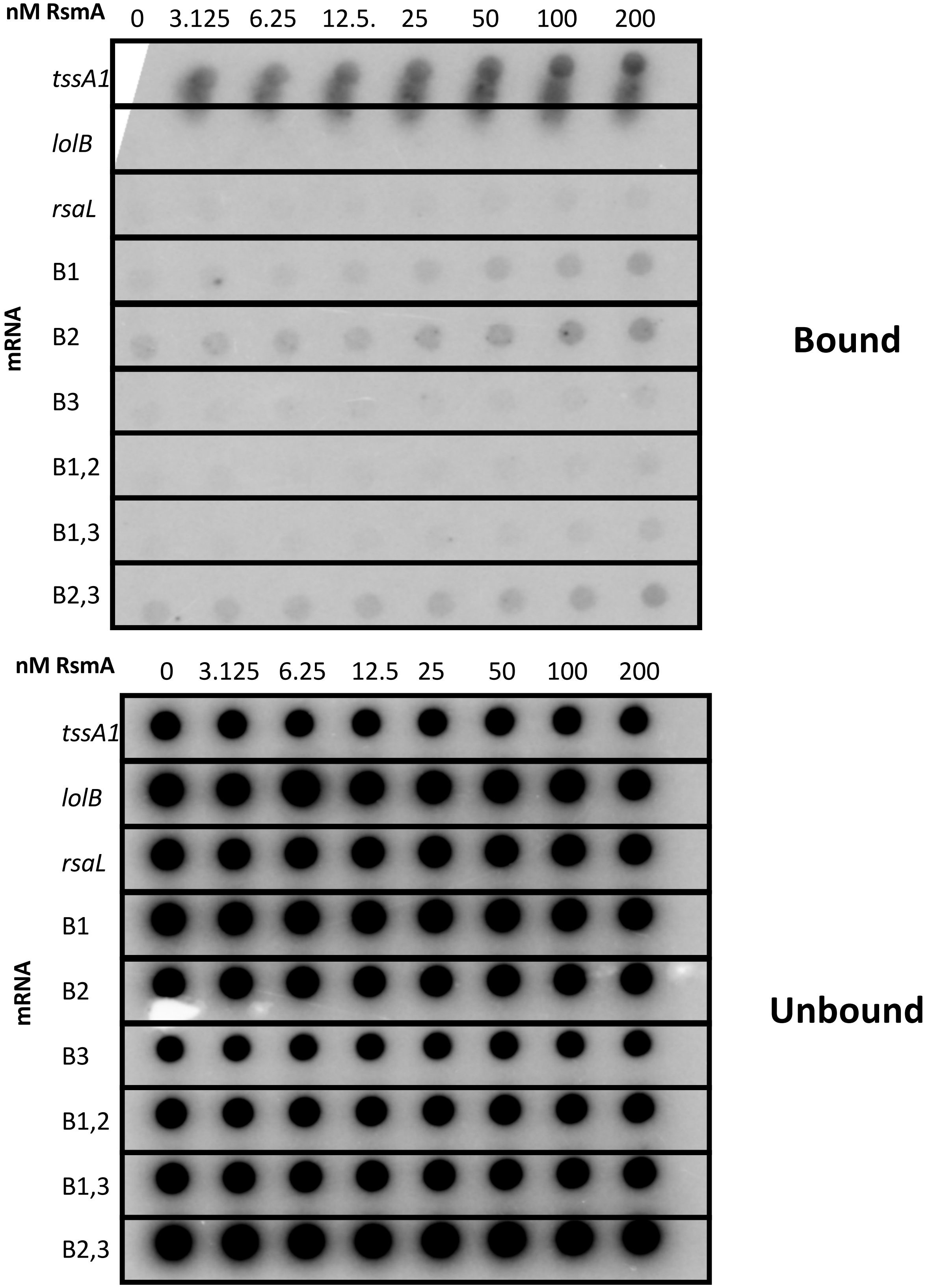

Figure 8: Filter-binding assay results for evaluating RsmA binding to *rsaL* and individual mutants at binding sites BS1, BS2, and BS3. Positive and negative controls *tssA1* and *lolB* are shown in the first two rows of the membrane. Bound fractions are shown on the top nitrocellulose membrane, and the unbound RNA flow through is shown on the bottom N+ membrane.

Supplemental Figure 9: Filter-binding images for *rsaL* binding mutants

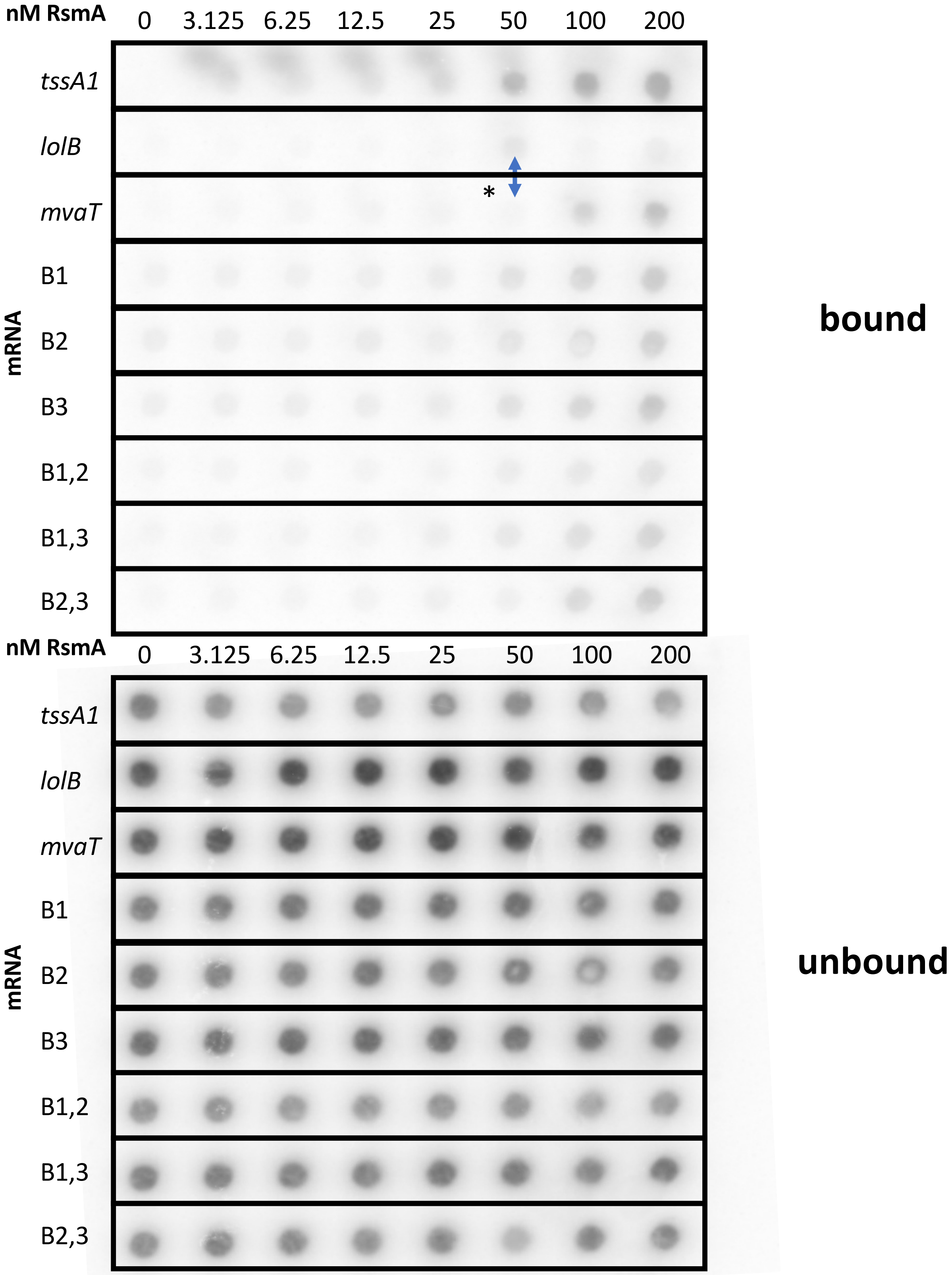

Figure 9: Filter-binding assay results for evaluating RsmA binding to *mvaT* and individual mutants at binding sites BS1, BS2, and BS3. Positive and negative controls *tssA1* and *lolB* are shown in the first two rows of the membrane. Bound fractions are shown on the top nitrocellulose membrane, and the unbound RNA flow through is shown on the bottom N+ membrane. * these two samples were switched due to pipetting error

Supplemental References

1. Mercante J, Edwards AN, Dubey AK, Babitzke P, Romeo T. Molecular Geometry of CsrA (RsmA) Binding to RNA and Its Implications for Regulated Expression. J Mol Biol [Internet]. 2009;392:511–28. Available from: http://dx.doi.org/10.1016/j.jmb.2009.07.034
2. Lapouge K, Sineva E, Lindell M, Starke K, Baker CS, Babitzke P, et al. Mechanism of hcnA mRNA recognition in the Gac/Rsm signal transduction pathway of Pseudomonas fluorescens. Mol Microbiol. 2007;66:341–56.
3. Schulmeyer KH, Diaz MR, Bair TB, Sanders W, Gode CJ, Laederach A, et al. Primary and secondary sequence structure requirements for recognition and discrimination of target RNAs by Pseudomonas aeruginosa RsmA and RsmF. J Bacteriol. 2016;198:2458–69.
4. Rajput A, Tsunemoto H, Sastry A V., Szubin R, Rychel K, Sugie J, et al. Machine learning from Pseudomonas aeruginosa transcriptomes identifies independently modulated sets of genes associated with known transcriptional regulators. Nucleic Acids Res. 2022;50:3658–72.
