# Supplementary material for: Thermodynamic modeling of RsmA - mRNA interactions capture novel direct binding across the Pseudomonas aeruginosa transcriptome
Source: Front Mol Biosci. 2025 Feb 20;12:1493891. doi: 10.3389/fmolb.2025.1493891 (PMC11882435; doi:10.3389/fmolb.2025.1493891)
Supplement: Supplementary file 6 [file DataSheet2.zip › Supplementary Binding Packet RsmA.pdf]

Modeled binding peaks for 1043 predicted targets of RsmA

PA14 gene ID	gene name	description	GO terms	KEGG pathways	overall affinity score in RT	predicted effect on translation	binding site predictions
PA14_00050	gyrB	DNA gyrase subunit B	DNA binding, DNA topoisomerase type II (ATP-hydrolyzing) activity, ATP binding, DNA topological change, chromosome		-26.28105	repression	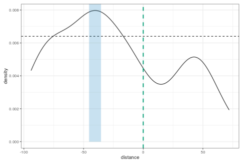
PA14_00090	glyS	glycyl-tRNA synthetase subunit beta	arginine-tRNA ligase activity, ATP binding, arginyl-tRNA aminoacylation, nucleotide binding, glycine-tRNA ligase activity, cytoplasm, glycyl-tRNA aminoacylation	Aminoacyl-tRNA biosynthesis	-25.86171	repression	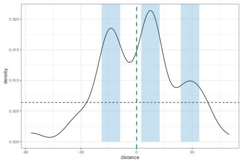
PA14_00160	NA	hypothetical protein	protein binding		-25.96560	no impact	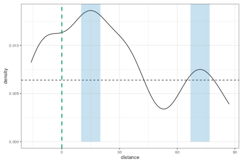
PA14_00210	NA	lysin domain-containing protein			-26.23488	repression	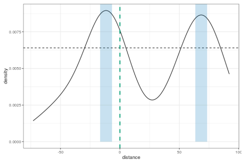
PA14_00230	NA	Rossmann fold nucleotide-binding protein	DNA mediated transformation		-26.91108	repression	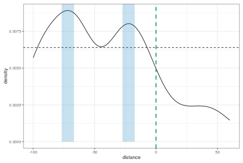
PA14_00250	qor	quinone oxidoreductase	oxidation-reduction process, oxidoreductase activity, zinc ion binding		-25.87822	repression	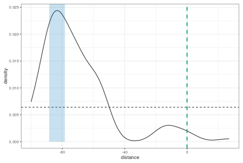
PA14_00290	aroE	shikimate 5-dehydrogenase	shikimate 3-dehydrogenase (NADP+) activity, shikimate metabolic process, NADP binding, oxidation-reduction process	Biosynthesis of amino acids, Biosynthesis of antibiotics, Biosynthesis of secondary metabolites, Metabolic pathways, Phenylalanine, tyrosine and tryptophan biosynthesis	-26.42002	repression	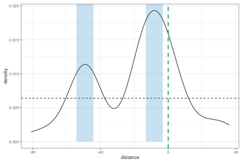
PA14_00340	NA	sulfate transporter	sulfate transport, sulfate transmembrane transporter activity, integral component of membrane		-25.84300	repression	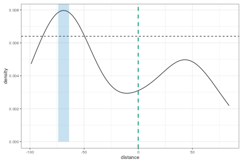
PA14_00530	NA	hypothetical protein			-26.77829	repression	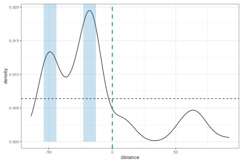
PA14_00580	NA	lipoprotein			-26.67633	repression	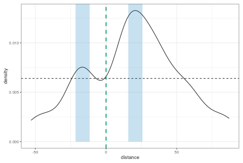

Modeled binding peaks for 1043 predicted targets of RsmA

PA14 gene ID	gene name	description	GO terms	KEGG pathways	overall affinity score in RT	predicted effect on translation	binding site predictions
PA14_00600	NA	transcriptional regulator	DNA binding		-26.14631	repression	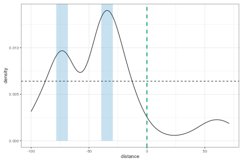
PA14_00630	NA	hypothetical protein			-26.95979	repression	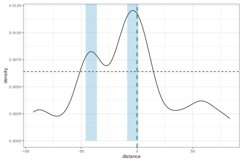
PA14_00660	NA	RNA 2'-phosphotransferase-like protein	phosphotransferase activity, alcohol group as acceptor, tRNA splicing, via endonucleolytic cleavage and ligation, transferase activity, transferring phosphorus-containing groups		-27.18575	repression	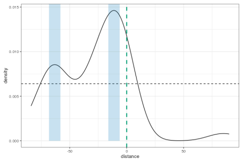
PA14_00730	NA	hypothetical protein			-25.87712	repression	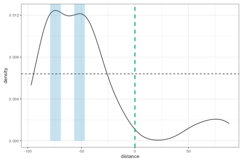
PA14_00740	NA	lipoprotein			-26.44604	repression	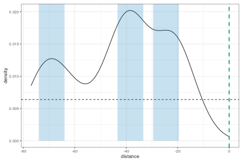
PA14_00820	tagQ1	TagQ1			-26.29270	repression	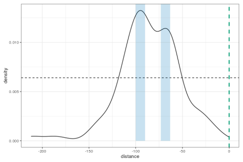
PA14_00875	ppkA	serine/threonine protein kinase PpkA	protein kinase activity, ATP binding, protein phosphorylation, positive regulation of protein secretion, protein serine/threonine kinase activity	Bacterial secretion system, Biofilm formation - Pseudomonas aeruginosa	-26.70750	repression	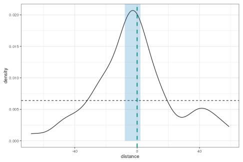
PA14_00990	tssA1	TssA1			-26.34988	repression	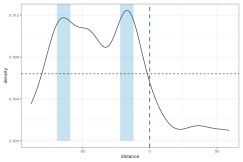
PA14_01010	hsiB1	HsiB1			-26.38080	repression	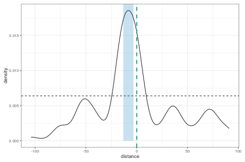
PA14_01030	hcp1	Hcp1			-26.72812	no impact	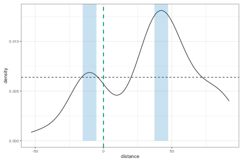

Modeled binding peaks for 1043 predicted targets of RsmA

PA14 gene ID	gene name	description	GO terms	KEGG pathways	overall affinity score in RT	predicted effect on translation	binding site predictions
PA14_01100	clpV1	ClpV1	protein metabolic process, ATP binding	Bacterial secretion system, Biofilm formation - Pseudomonas aeruginosa	-25.80157	repression	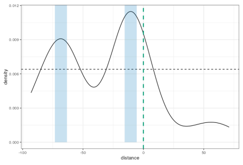
PA14_01110	vgrG1a	VgrG1a			-26.97112	repression	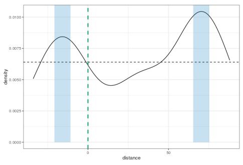
PA14_01120	tsi6	Tsi6			-26.44927	repression	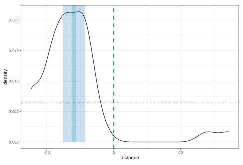
PA14_01170	NA	hypothetical protein			-26.34650	repression	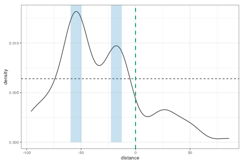
PA14_01180	NA	hypothetical protein			-26.19782	no impact	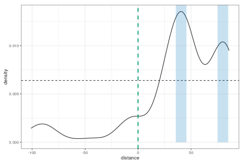
PA14_01200	NA	hypothetical protein			-26.15251	repression	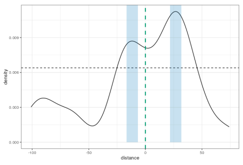
PA14_01240	NA	carbonic anhydrase	carbon utilization, carbonate dehydratase activity, zinc ion binding	Nitrogen metabolism	-26.50859	no impact	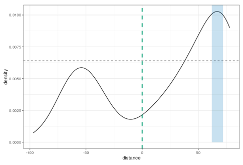
PA14_01290	coxB	cytochrome c oxidase subunit II	cytochrome-c oxidase activity, copper ion binding, membrane, electron transfer activity, heme binding, integral component of membrane, oxidoreductase activity, electron transport chain	Metabolic pathways, Oxidative phosphorylation	-26.30912	repression	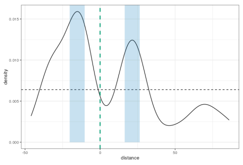
PA14_01300	coxA	cytochrome c oxidase subunit I	oxidation-reduction process, cytochrome-c oxidase activity, respiratory chain complex IV, aerobic respiration, integral component of membrane, heme binding, electron transport coupled proton transport	Metabolic pathways, Oxidative phosphorylation	-26.12748	no impact	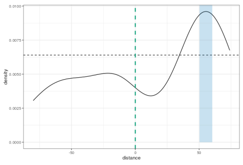
PA14_01340	NA	hypothetical protein	membrane		-26.61273	repression	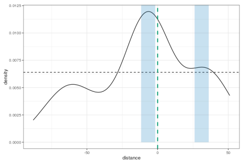

PA14 gene ID	gene name	description	GO terms	KEGG pathways	overall affinity score in RT	predicted effect on translation	binding site predictions
Modeled binding peaks for 1043 predicted targets of RsmA							
PA14_01380	NA	protoheme IX farnesyltransferase	integral component of membrane, transferase activity, transferring alkyl or aryl (other than methyl) groups, protoheme IX farnesyltransferase activity, heme O biosynthetic process	Biosynthesis of secondary metabolites, Metabolic pathways, Oxidative phosphorylation, Porphyrin and chlorophyll metabolism	-26.83773	repression	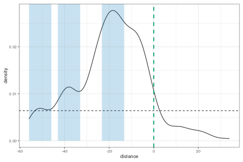
PA14_01490	NA	hemolysin	hemolysis by symbiont of host erythrocytes		-26.91096	repression	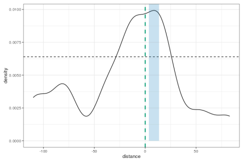
PA14_01500	NA	transcriptional regulator	DNA-binding transcription factor activity, regulation of transcription, DNA-templated		-27.00465	repression	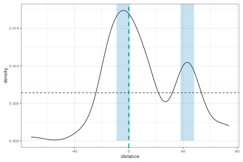
PA14_01600	NA	aldehyde dehydrogenase	oxidoreductase activity, oxidoreductase activity, acting on the aldehyde or oxo group of donors, NAD or NADP as acceptor, oxidation-reduction process, methylmalonate-semialdehyde dehydrogenase (acylating) activity, beta-alanine biosynthetic process	beta-Alanine metabolism, Carbon metabolism, Inositol phosphate metabolism, Metabolic pathways, Propanoate metabolism, Valine, leucine and isoleucine degradation	-27.26365	repression	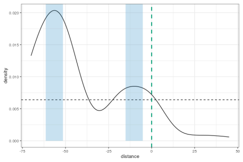
PA14_01660	NA	guanine deaminase	guanine catabolic process, zinc ion binding, guanine deaminase activity, hydrolase activity, hydrolase activity, acting on carbon-nitrogen (but not peptide) bonds	drosopterin and aurodrosopterin biosynthesis, guanosine nucleotides degradation II, guanosine nucleotides degradation III, Metabolic pathways, Purine metabolism, Purine metabolism, purine nucleobases degradation II (anaerobic)	-26.51067	repression	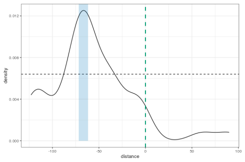
PA14_01810	NA	oxidoreductase	oxidoreductase activity, oxidation-reduction process		-25.84014	repression	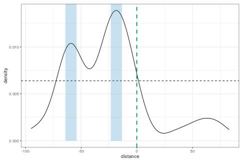
PA14_01940	NA	RND efflux membrane fusion protein	membrane, transmembrane transporter activity, transmembrane transport		-26.67288	repression	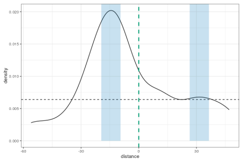
PA14_01970	NA	RND efflux transporter	membrane, transmembrane transporter activity, transmembrane transport		-25.82186	repression	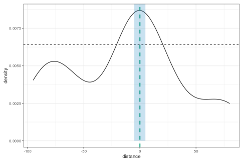
PA14_02020	NA	outer membrane porin	integral component of membrane		-26.21154	no impact	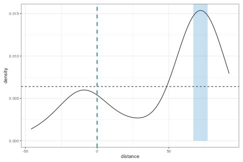
PA14_02140	NA	hypothetical protein			-26.04698	repression	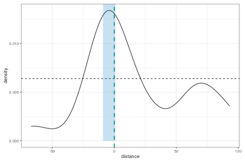

Modeled binding peaks for 1043 predicted targets of RsmA

PA14 gene ID	gene name	description	GO terms	KEGG pathways	overall affinity score in RT	predicted effect on translation	binding site predictions
PA14_02150	NA	hypothetical protein	signal transduction, integral component of membrane, catalytic activity		-26.27045	repression	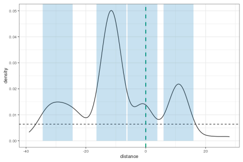
PA14_02200	NA	chemotaxis protein methyltransferase	S-adenosylmethionine-dependent methyltransferase activity	Bacterial chemotaxis, Two-component system	-26.32595	no impact	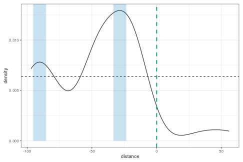
PA14_02220	NA	chemotaxis transducer	signal transduction, membrane, transmembrane signaling receptor activity, chemotaxis, integral component of membrane	Bacterial chemotaxis, Two-component system	-27.05187	repression	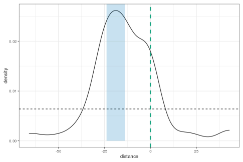
PA14_02260	NA	two-component response regulator	phosphorelay signal transduction system	Bacterial chemotaxis, Two-component system	-25.75452	repression	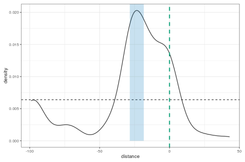
PA14_02300	fabG	3-ketoacyl-ACP reductase			-25.92959	repression	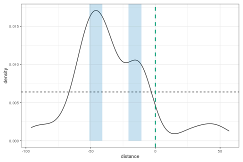
PA14_02310	atsA	arylsulfatase	catalytic activity, sulfuric ester hydrolase activity, arylsulfatase activity, phosphoric diester hydrolase activity	Sphingolipid metabolism	-26.04159	repression	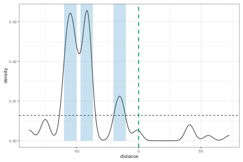
PA14_02370	NA	porin	integral component of membrane		-26.88642	no impact	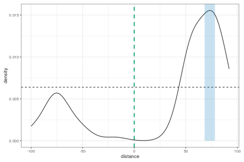
PA14_02435	NA	hypothetical protein	oxidoreductase activity, oxidation-reduction process		-26.10635	repression	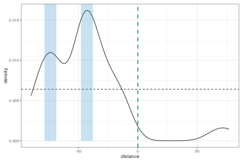
PA14_02460	NA	NAD(P) transhydrogenase subunit alpha part 2			-25.75933	repression	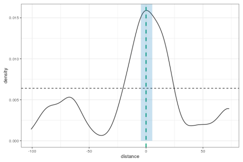
PA14_02570	mdcC	malonate decarboxylase subunit delta			-25.77812	repression	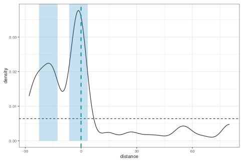

Modeled binding peaks for 1043 predicted targets of RsmA

PA14 gene ID	gene name	description	GO terms	KEGG pathways	overall affinity score in RT	predicted effect on translation	binding site predictions
PA14_02590	mdcE	malonate decarboxylase subunit gamma	cellular carbohydrate metabolic process, ligase activity	3-hydroxypropanoate cycle, 3-hydroxypropanoate/4-hydroxybutanate cycle, candicidin biosynthesis, fatty acid biosynthesis initiation I, glyoxylate assimilation, malonate degradation II (biotin-dependent)	-25.89917	repression	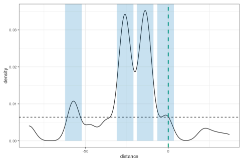
PA14_02740	NA	class II aldolase/adducin domain-containing protein			-25.84622	repression	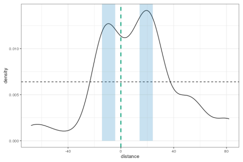
PA14_02790	pcaF	beta-ketoadipyl CoA thiolase	transferase activity, transferring acyl groups other than amino-acyl groups, catalytic activity, 3,4-dihydroxybenzoate catabolic process	Benzoate degradation, Microbial metabolism in diverse environments	-25.76400	repression	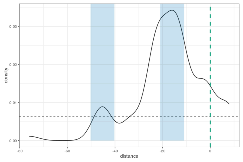
PA14_02810	pcaT	dicarboxylic acid transporter PcaT	integral component of membrane, transmembrane transporter activity, transmembrane transport, integral component of plasma membrane		-25.91422	repression	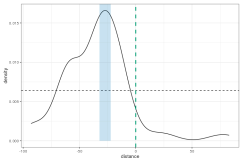
PA14_02890	NA	hypothetical protein	cell outer membrane		-26.00684	no impact	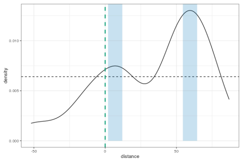
PA14_03170	NA	hypothetical protein	DNA integration, nucleic acid binding		-25.76743	repression	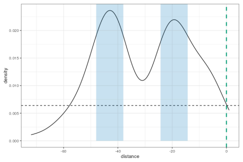
PA14_03200	tle3	Type VI effector Tle3			-26.28507	repression	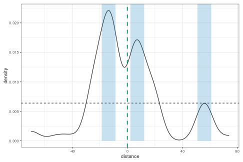
PA14_03220	vgrG2b	VgrG2b			-27.48331	repression	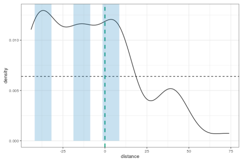
PA14_03510	NA	hypothetical protein			-25.93118	repression	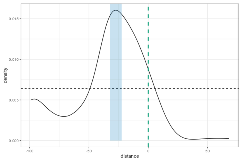
PA14_03530	NA	transcriptional regulator	regulation of transcription, DNA-templated, DNA-binding transcription factor activity		-26.15826	repression	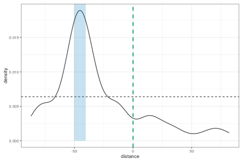

Modeled binding peaks for 1043 predicted targets of RsmA

PA14 gene ID	gene name	description	GO terms	KEGG pathways	overall affinity score in RT	predicted effect on translation	binding site predictions
PA14_03610	NA	Zn-dependent protease with chaperone function	metalloendopeptidase activity, proteolysis		-26.17998	repression	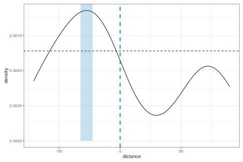
PA14_03680	cysT	sulfate transport protein CysT	sulfate transport, ATPase-coupled sulfate transmembrane transporter activity, membrane, plasma membrane, transmembrane transport	ABC transporters, Sulfur metabolism	-26.41424	repression	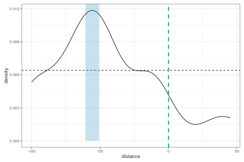
PA14_03760	NA	sodium:solute symporter	membrane, transmembrane transporter activity, transmembrane transport		-26.22386	no impact	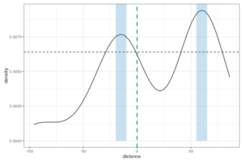
PA14_03930	spuE	polyamine transport protein	polyamine transport, polyamine binding, periplasmic space	ABC transporters	-26.04000	repression	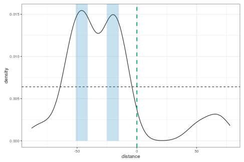
PA14_04080	NA	ABC transporter permease	membrane, transmembrane transport, integral component of membrane, transmembrane transporter activity, ATP-binding cassette (ABC) transporter complex, nitrogen compound transport	ABC transporters	-26.71677	repression	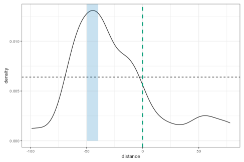
PA14_04100	NA	hypothetical protein			-26.01195	repression	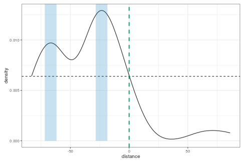
PA14_04250	NA	ABC transporter ATP-binding protein	ATP binding, transmembrane transporter activity, ATP-binding cassette (ABC) transporter complex, transmembrane transport, ATPase activity, ATPase-coupled polyamine transmembrane transporter activity, polyamine transport	ABC transporters	-25.99923	no impact	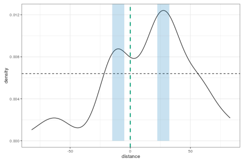
PA14_04330	NA	hypothetical protein			-27.43375	repression	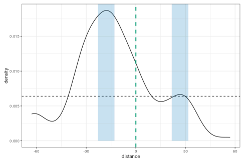
PA14_04410	ptsP	phosphoenolpyruvate-protein phosphotransferase PtsP	transferase activity, transferring phosphorus-containing groups, protein binding, phosphorylation, phosphoenolpyruvate-dependent sugar phosphotransferase system, catalytic activity	Phosphotransferase system (PTS)	-26.54031	repression	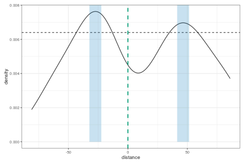
PA14_04440	NA	permease	integral component of membrane		-26.02724	repression	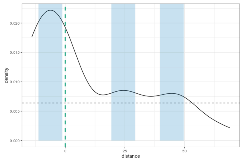

Modeled binding peaks for 1043 predicted targets of RsmA							
PA14 gene ID	gene name	description	GO terms	KEGG pathways	overall affinity score in RT	predicted effect on translation	binding site predictions
PA14_04490	NA	hypothetical protein			-26.00842	no impact	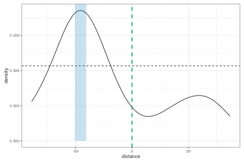
PA14_04560	NA	hypothetical protein			-26.42844	repression	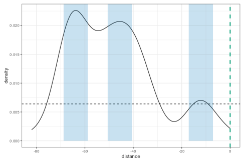
PA14_04700	NA	hypothetical protein	protein binding		-26.15406	repression	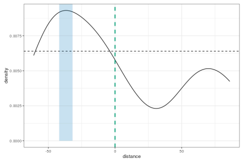
PA14_04950	mtgA	monofunctional biosynthetic peptidoglycan transglycosylase	peptidoglycan biosynthetic process, peptidoglycan-based cell wall, integral component of membrane, transferase activity, transferring pentosyl groups	Peptidoglycan biosynthesis	-26.61698	repression	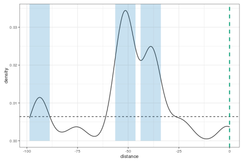
PA14_04960	NA	hypothetical protein			-26.63033	repression	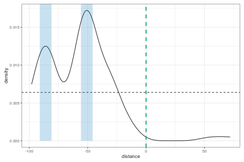
PA14_05030	NA	hypothetical protein			-25.96998	repression	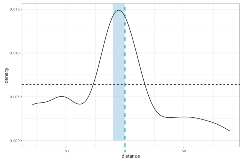
PA14_05050	NA	deoxyribonucleotide triphosphate pyrophosphatase	nucleoside triphosphate catabolic process, nucleoside-triphosphate diphosphatase activity, nucleoside-triphosphatase activity	Purine metabolism	-25.98232	repression	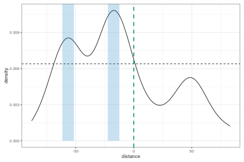
PA14_05060	NA	hypothetical protein			-25.75795	repression	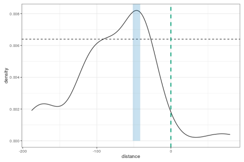
PA14_05110	NA	hypothetical protein			-25.76702	repression	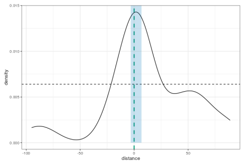
PA14_05180	pilT	twitching motility protein PilT	ATP binding, type IV pilus, pilus retraction, type IV pilus-dependent motility		-25.86196	repression	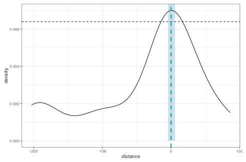

Modeled binding peaks for 1043 predicted targets of RsmA

PA14 gene ID	gene name	description	GO terms	KEGG pathways	overall affinity score in RT	predicted effect on translation	binding site predictions
PA14_05310	gshB	glutathione synthetase	glutathione synthase activity, ATP binding, glutathione biosynthetic process, metal ion binding	Cysteine and methionine metabolism, Glutathione metabolism, Metabolic pathways	-25.79507	no impact	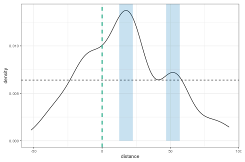
PA14_05320	pilG	twitching motility protein PilG	phosphorelay signal transduction system	Biofilm formation - Pseudomonas aeruginosa, Two-component system	-26.10482	repression	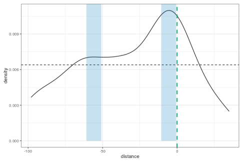
PA14_05390	chpA	ChpA	phosphorelay signal transduction system, chemotaxis, signal transduction, phosphorelay sensor kinase activity, protein histidine kinase activity, cytoplasm, phosphorylation, transferase activity, transferring phosphorus-containing groups	Biofilm formation - Pseudomonas aeruginosa, Two-component system	-27.23070	repression	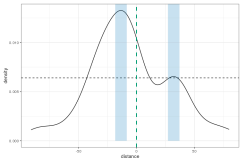
PA14_05540	mexB	RND multidrug efflux transporter MexB	membrane, transmembrane transporter activity, transmembrane transport, efflux transmembrane transporter activity, integral component of membrane, xenobiotic transport	beta-Lactam resistance, Cationic antimicrobial peptide (CAMP) resistance	-26.63600	repression	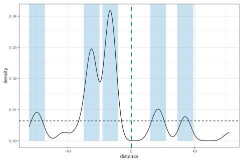
PA14_05550	oprM	major intrinsic multiple antibiotic resistance efflux outer membrane protein OprM precursor	membrane, transmembrane transporter activity, transmembrane transport, efflux transmembrane transporter activity, drug transmembrane transporter activity	beta-Lactam resistance, Quorum sensing	-25.93586	repression	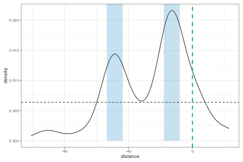
PA14_05620	sahH	S-adenosyl-L-homocysteine hydrolase	adenosylhomocysteinase activity	<i>S</i>-adenosyl-L-methionine cycle II, Cysteine and methionine metabolism, Cysteine and methionine metabolism, Metabolic pathways	-25.77400	repression	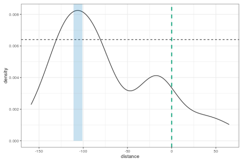
PA14_05890	NA	stomatin-like protein	membrane		-26.62783	repression	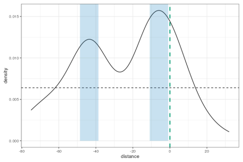
PA14_06090	NA	hypothetical protein			-26.56362	no impact	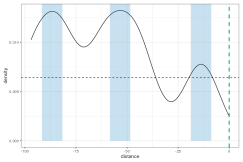
PA14_06150	NA	hypothetical protein			-25.93607	repression	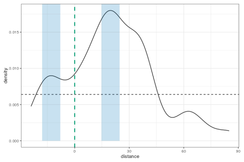
PA14_06280	NA	hypothetical protein			-26.29357	no impact	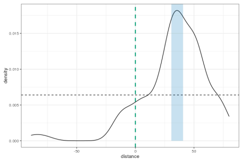

Modeled binding peaks for 1043 predicted targets of RsmA

PA14 gene ID	gene name	description	GO terms	KEGG pathways	overall affinity score in RT	predicted effect on translation	binding site predictions
PA14_06390	NA	hypothetical protein			-27.06072	repression	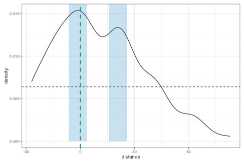
PA14_06400	NA	LysR family transcriptional regulator	DNA-binding transcription factor activity, regulation of transcription, DNA-templated		-25.95936	repression	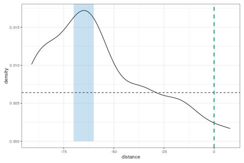
PA14_06420	NA	hypothetical protein	catalytic activity, carbohydrate metabolic process	γ-glutamyl cycle, 5-oxo-L-proline metabolism, Glutathione metabolism	-26.03131	no impact	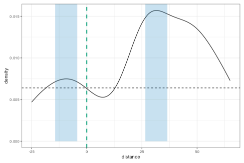
PA14_06620	NA	acyl-CoA dehydrogenase	oxidoreductase activity, acting on the CH-CH group of donors, flavin adenine dinucleotide binding, oxidation-reduction process		-26.01378	repression	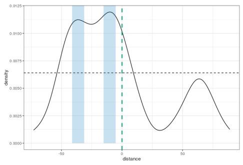
PA14_06750	nirS	nitrite reductase	electron transfer activity, heme binding	Microbial metabolism in diverse environments, Nitrogen metabolism	-26.54933	repression	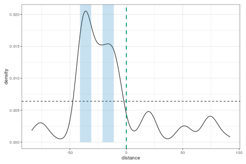
PA14_06890	NA	hypothetical protein	catalytic activity, molybdenum ion binding, pyridoxal phosphate binding		-25.83427	repression	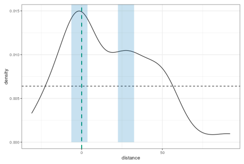
PA14_06920	NA	class III pyridoxal phosphate-dependent aminotransferase	transaminase activity, pyridoxal phosphate binding, catalytic activity	2-Oxocarboxylic acid metabolism, Arginine biosynthesis, Biosynthesis of amino acids, Biosynthesis of antibiotics, Biosynthesis of secondary metabolites, Lysine biosynthesis, Metabolic pathways, Microbial metabolism in diverse environments	-26.20679	repression	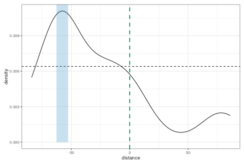
PA14_06940	NA	hypothetical protein			-26.38598	repression	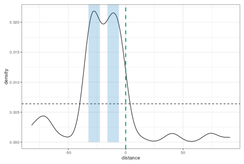
PA14_07010	NA	hypothetical protein	membrane, integral component of membrane		-26.50039	repression	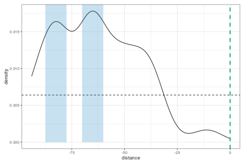
PA14_07070	NA	hypothetical protein	oxidoreductase activity, oxidation-reduction process		-26.43337	repression	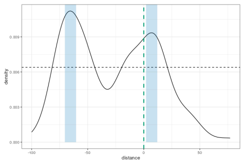

Modeled binding peaks for 1043 predicted targets of RsmA

PA14 gene ID	gene name	description	GO terms	KEGG pathways	overall affinity score in RT	predicted effect on translation	binding site predictions
PA14_07200	NA	hypothetical protein			-26.63206	repression	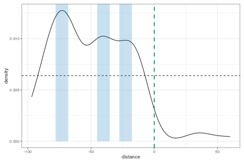
PA14_07210	NA	hypothetical protein			-26.51305	repression	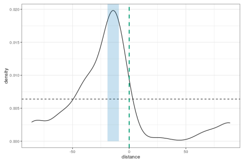
PA14_07250	NA	hypothetical protein			-26.45753	repression	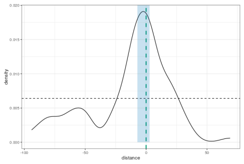
PA14_07300	NA	hypothetical protein			-26.33994	repression	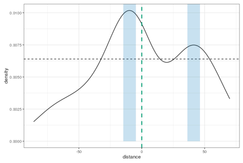
PA14_07330	NA	hypothetical protein	integral component of membrane		-26.87012	repression	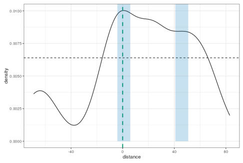
PA14_07370	NA	hypothetical protein	integral component of membrane		-26.04589	repression	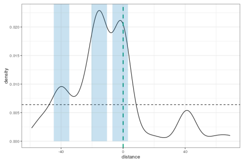
PA14_07380	NA	hypothetical protein			-26.97839	repression	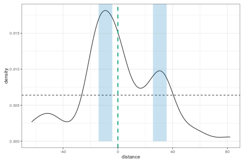
PA14_07420	NA	hypothetical protein			-25.76632	no impact	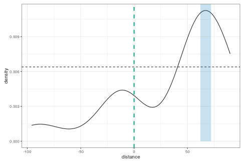
PA14_07520	rpoD	RNA polymerase sigma factor RpoD	DNA-binding transcription factor activity, DNA-templated transcription, initiation, regulation of transcription, DNA-templated, DNA binding, sigma factor activity		-25.94098	no impact	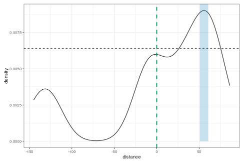
PA14_07590	folB	dihydroneopterin aldolase	dihydroneopterin aldolase activity, folic acid-containing compound metabolic process	Folate biosynthesis, Metabolic pathways	-26.55826	repression	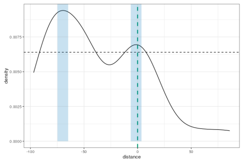

Modeled binding peaks for 1043 predicted targets of RsmA

PA14 gene ID	gene name	description	GO terms	KEGG pathways	overall affinity score in RT	predicted effect on translation	binding site predictions
PA14_07630	NA	hypothetical protein			-25.88896	no impact	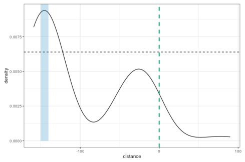
PA14_07680	NA	hypothetical protein	protein kinase activity		-27.09370	repression	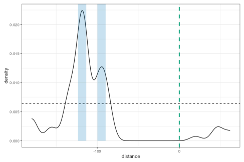
PA14_07840	NA	two-component response regulator	phosphorelay signal transduction system, regulation of transcription, DNA-templated, DNA binding		-26.09922	no impact	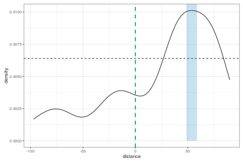
PA14_07850	NA	ABC transporter substrate-binding protein			-26.15679	repression	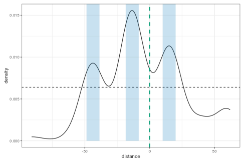
PA14_07900	NA	ABC transporter permease	membrane, transmembrane transport	Quorum sensing	-26.35649	repression	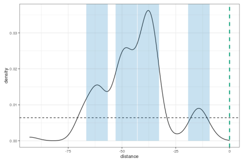
PA14_08020	NA	bacteriophage protein			-26.81742	repression	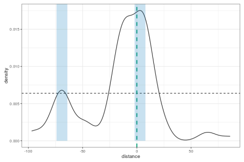
PA14_08060	NA	tail fiber assembly protein			-26.23744	repression	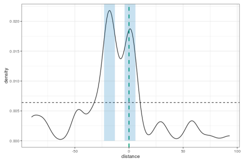
PA14_08160	NA	lytic enzyme			-26.58651	repression	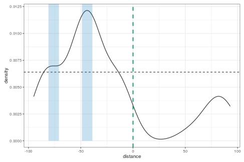
PA14_08200	NA	hypothetical protein			-26.00534	repression	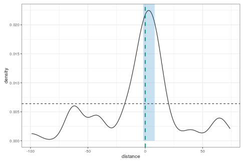
PA14_08210	NA	hypothetical protein			-25.94224	repression	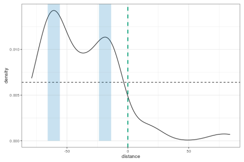

PA14 gene ID	gene name	description	GO terms	KEGG pathways	overall affinity score in RT	predicted effect on translation	binding site predictions
Modeled binding peaks for 1043 predicted targets of RsmA							
PA14_08240	NA	hypothetical protein			-26.80327	no impact	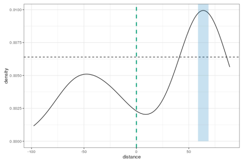
PA14_08250	NA	hypothetical protein			-26.25859	repression	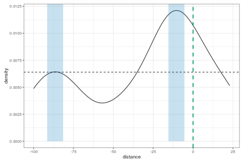
PA14_08300	NA	phage-related protein, tail component	protein binding		-26.38628	repression	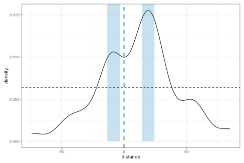
PA14_08310	NA	hypothetical protein			-25.88211	repression	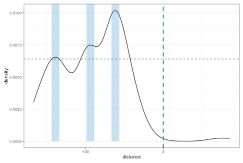
PA14_08320	NA	hypothetical protein			-25.76658	no impact	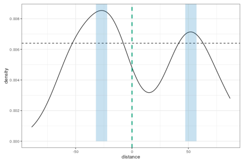
PA14_08350	trpD	anthranilate phosphoribosyltransferase	tryptophan biosynthetic process, anthranilate phosphoribosyltransferase activity, transferase activity, transferring glycosyl groups	Biosynthesis of amino acids, Biosynthesis of antibiotics, Biosynthesis of secondary metabolites, Metabolic pathways, Phenylalanine, tyrosine and tryptophan biosynthesis	-27.06760	repression	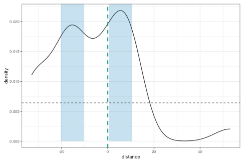
PA14_08420	NA	HIT family protein	catalytic activity		-25.96506	repression	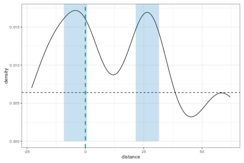
PA14_08480	argC	N-acetyl-gamma-glutamyl-phosphate reductase	cellular amino acid biosynthetic process, oxidoreductase activity, acting on the aldehyde or oxo group of donors, NAD or NADP as acceptor, protein dimerization activity, N-acetyl-gamma-glutamyl-phosphate reductase activity, arginine biosynthetic process, oxidation-reduction process, NAD binding	2-Oxocarboxylic acid metabolism, Arginine biosynthesis, Arginine biosynthesis, Biosynthesis of amino acids, Biosynthesis of antibiotics, Biosynthesis of secondary metabolites, L-arginine biosynthesis III (via <i>N</i>-acetyl-L-citrulline), L-arginine biosynthesis IV (archaebacteria), Metabolic pathways	-26.28718	repression	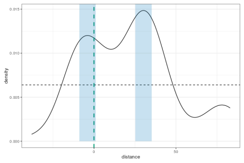
PA14_08630	NA	pantothenate kinase	pantothenate kinase activity	Metabolic pathways, Pantothenate and CoA biosynthesis	-26.33798	repression	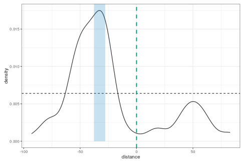
PA14_08680	tufB	elongation factor Tu	translation elongation factor activity, GTP binding, translational elongation, GTPase activity		-26.59689	repression	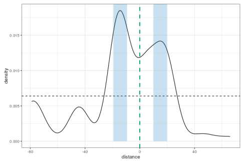

Modeled binding peaks for 1043 predicted targets of RsmA							
PA14 gene ID	gene name	description	GO terms	KEGG pathways	overall affinity score in RT	predicted effect on translation	binding site predictions
PA14_08730	rplA	50S ribosomal protein L1	RNA binding, structural constituent of ribosome, translation, large ribosomal subunit	Ribosome	-25.81372	repression	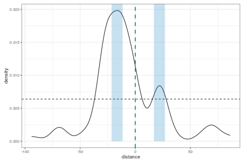
PA14_08760	rpoB	DNA-directed RNA polymerase subunit beta	DNA-directed 5'-3' RNA polymerase activity, transcription, DNA-templated, ribonucleoside binding, DNA binding	Metabolic pathways, Purine metabolism, Pyrimidine metabolism, RNA polymerase	-25.76213	no impact	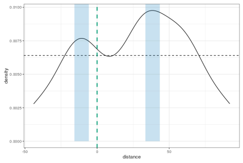
PA14_08900	rplV	50S ribosomal protein L22	structural constituent of ribosome, ribosome, translation, large ribosomal subunit	Ribosome	-27.00943	repression	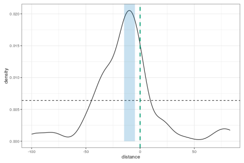
PA14_08910	rpsC	30S ribosomal protein S3	structural constituent of ribosome, translation, small ribosomal subunit, nucleic acid binding, RNA binding	Ribosome	-26.74368	repression	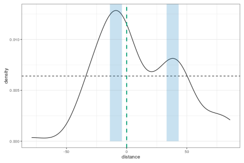
PA14_08950	rplN	50S ribosomal protein L14	structural constituent of ribosome, ribosome, translation, large ribosomal subunit	Ribosome	-25.80334	repression	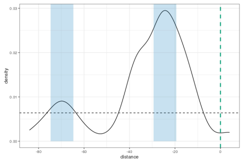
PA14_08960	rplX	50S ribosomal protein L24	structural constituent of ribosome, ribosome, translation	Ribosome	-25.98982	repression	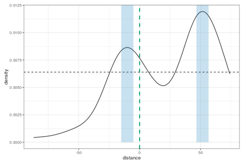
PA14_08970	rplE	50S ribosomal protein L5	structural constituent of ribosome, ribosome, translation	Ribosome	-26.33277	no impact	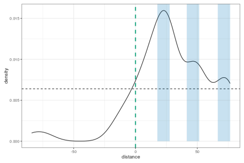
PA14_09270	pchE	dihydroaeruginoic acid synthetase	catalytic activity, phosphopantetheine binding	Biosynthesis of siderophore group nonribosomal peptides, Pyochelin synthesis	-26.00953	repression	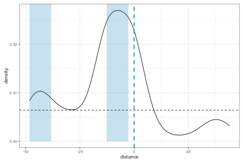
PA14_09340	fptA	Fe(III)-pyochelin outer membrane receptor	cell outer membrane, siderophore uptake transmembrane transporter activity, siderophore transport, signaling receptor activity		-26.44751	repression	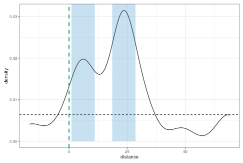
PA14_09410	phzG1	pyrodoxamine 5'-phosphate oxidase	oxidoreductase activity, acting on the CH-NH2 group of donors, oxidation-reduction process, pyridoxamine-phosphate oxidase activity, pyridoxine biosynthetic process, FMN binding, cofactor binding, phenazine biosynthetic process	Phenazine biosynthesis, Quorum sensing	-28.31133	repression	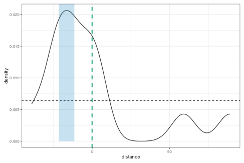

Modeled binding peaks for 1043 predicted targets of RsmA

PA14 gene ID	gene name	description	GO terms	KEGG pathways	overall affinity score in RT	predicted effect on translation	binding site predictions
PA14_09420	phzF1	phenazine biosynthesis protein	catalytic activity, biosynthetic process	Phenazine biosynthesis, Quorum sensing	-26.44280	NA	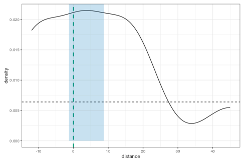
PA14_09440	phzE1	phenazine biosynthesis protein PhzE	biosynthetic process	Phenazine biosynthesis, Quorum sensing	-25.78680	NA	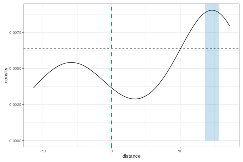
PA14_09520	mexI	RND efflux transporter	membrane, transmembrane transporter activity, transmembrane transport	Quorum sensing	-26.04972	repression	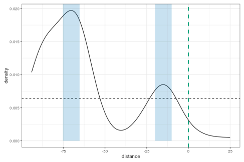
PA14_09600	ddlA	D-alanine--D-alanine ligase	cytoplasm, D-alanine-D-alanine ligase activity, ATP binding, metal ion binding	D-Alanine metabolism, Metabolic pathways, Peptidoglycan biosynthesis, Vancomycin resistance	-26.16340	repression	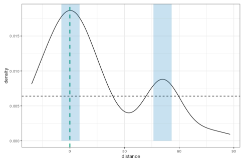
PA14_09690	NA	two-component response regulator	regulation of transcription, DNA-templated, phosphorelay signal transduction system, DNA binding		-26.03051	repression	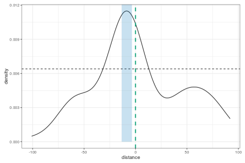
PA14_09700	NA	monooxygenase	FAD binding		-25.78646	repression	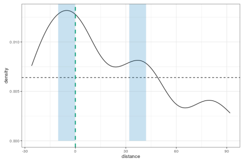
PA14_09710	NA	aldehyde dehydrogenase	oxidoreductase activity, oxidoreductase activity, acting on the aldehyde or oxo group of donors, NAD or NADP as acceptor, oxidation-reduction process	Arginine and proline metabolism, Ethanol utilization; second step, Metabolic pathways	-26.08322	repression	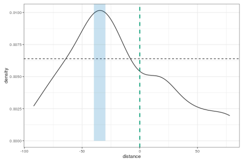
PA14_09770	souR	sarcosine oxidation and utilization regulator SouR	DNA-binding transcription factor activity, regulation of transcription, DNA-templated, sequence-specific DNA binding, DNA binding		-27.54743	no impact	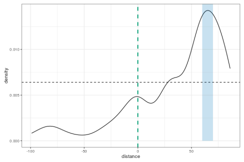
PA14_09820	NA	acetolactate synthase	catalytic activity, thiamine pyrophosphate binding, magnesium ion binding	2-Oxocarboxylic acid metabolism, Biosynthesis of amino acids, Biosynthesis of antibiotics, Biosynthesis of secondary metabolites, Butanoate metabolism, C5-Branched dibasic acid metabolism, Metabolic pathways, Pantothenate and CoA biosynthesis, Valine, leucine and isoleucine biosynthesis	-26.65378	repression	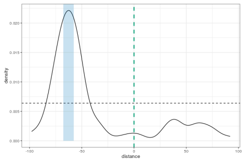
PA14_09920	NA	translation initiation inhibitor			-26.04459	repression	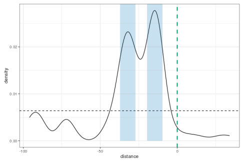

Modeled binding peaks for 1043 predicted targets of RsmA

PA14 gene ID	gene name	description	GO terms	KEGG pathways	overall affinity score in RT	predicted effect on translation	binding site predictions
PA14_09950	NA	oxidoreductase	oxidoreductase activity, oxidation-reduction process		-26.01042	repression	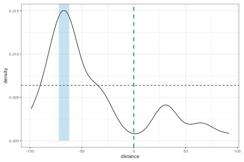
PA14_10200	NA	TonB-dependent receptor protein			-25.78131	repression	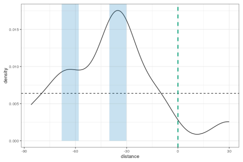
PA14_10240	NA	branched-chain alpha-keto acid dehydrogenase subunit E2			-26.49139	no impact	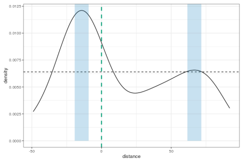
PA14_10360	NA	hypothetical protein			-26.59829	repression	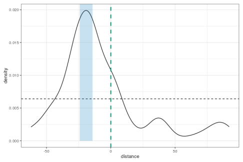
PA14_10370	NA	hypothetical protein	oxidoreductase activity, flavin adenine dinucleotide binding, oxidation-reduction process, FAD binding, catalytic activity	Bile acid biosynthesis, Zeatin biosynthesis	-26.67096	repression	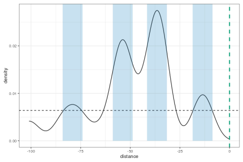
PA14_10490	NA	hypothetical protein			-25.92770	repression	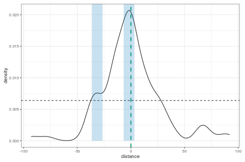
PA14_10500	NA	cbb3-type cytochrome c oxidase subunit I	cytochrome-c oxidase activity, oxidation-reduction process, aerobic respiration, integral component of membrane, heme binding, plasma membrane respiratory chain complex IV	Metabolic pathways, Oxidative phosphorylation, Two-component system	-26.32564	no impact	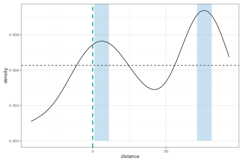
PA14_10660	NA	transcriptional regulator	regulation of transcription, DNA-templated, DNA-binding transcription factor activity, sequence-specific DNA binding, DNA binding		-26.16556	repression	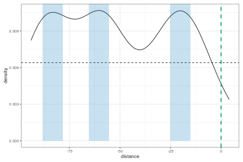
PA14_10670	aph	aminoglycoside 3'-phosphotransferase type IIB	ATP binding, phosphotransferase activity, alcohol group as acceptor, response to antibiotic		-26.00929	no impact	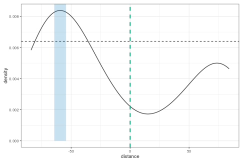
PA14_10840	NA	dehydrogenase			-26.20237	repression	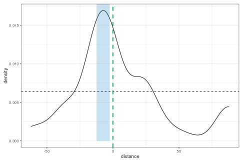

Modeled binding peaks for 1043 predicted targets of RsmA

PA14 gene ID	gene name	description	GO terms	KEGG pathways	overall affinity score in RT	predicted effect on translation	binding site predictions
PA14_11050	NA	hypothetical protein			-26.15485	repression	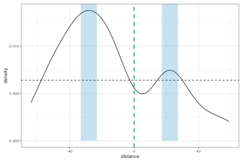
PA14_11060	cupB1	fimbrial subunit CupB1	cell adhesion, pilus		-26.02667	repression	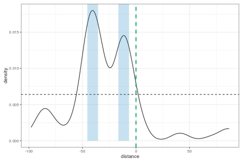
PA14_11080	cupB3	usher CupB3	protein binding, pilus assembly, fimbrial usher porin activity, membrane		-25.96571	repression	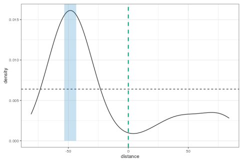
PA14_11100	cupB5	adhesive protein CupB5			-26.27332	repression	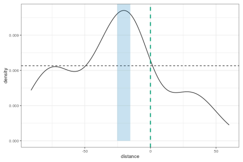
PA14_11120	NA	response regulator	phosphorelay signal transduction system, regulation of transcription, DNA-templated, DNA binding	Two-component system	-26.17816	repression	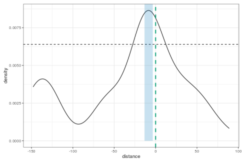
PA14_11150	NA	transcriptional regulator	DNA binding		-25.95858	repression	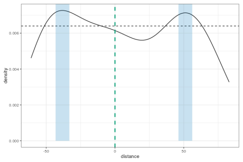
PA14_11170	NA	hypothetical protein			-26.64455	repression	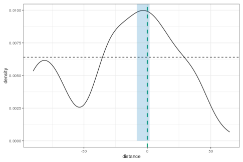
PA14_11180	NA	transcriptional regulator	regulation of transcription, DNA-templated, DNA binding		-26.11965	repression	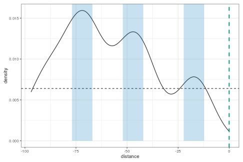
PA14_11230	NA	hypothetical protein			-26.66498	repression	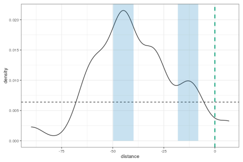
PA14_11420	ribB	bifunctional 3,4-dihydroxy-2-butanone 4-phosphate synthase/GTP cyclohydrolase II-like protein	3,4-dihydroxy-2-butanone-4-phosphate synthase activity, riboflavin biosynthetic process	Biosynthesis of secondary metabolites, Metabolic pathways, Riboflavin metabolism	-26.13107	no impact	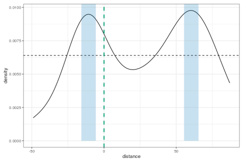

Modeled binding peaks for 1043 predicted targets of RsmA

PA14 gene ID	gene name	description	GO terms	KEGG pathways	overall affinity score in RT	predicted effect on translation	binding site predictions
PA14_11680	NA	two-component regulator	phosphorelay signal transduction system, DNA binding, regulation of transcription, DNA-templated		-26.17867	repression	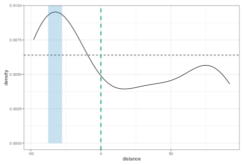
PA14_11845	mpl	UDP-N-acetylmuramate:L-alanyl-gamma-D-glutamyl- meso-diaminopimelate ligase	ATP binding, biosynthetic process, ligase activity, peptidoglycan biosynthetic process, acid-amino acid ligase activity, cell wall organization, cellular response to antibiotic		-25.97372	no impact	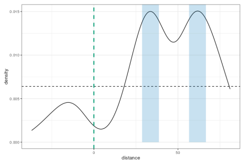
PA14_12100	dacC	D-ala-D-ala-carboxypeptidase	carboxypeptidase activity, proteolysis, serine-type D-Ala-D-Ala carboxypeptidase activity	Metabolic pathways, Peptidoglycan biosynthesis	-26.27169	repression	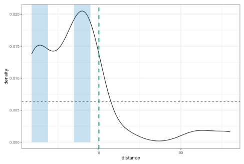
PA14_12130	lis	lipoyl synthase	catalytic activity, iron-sulfur cluster binding, lipoate biosynthetic process, lipoate synthase activity, 4 iron, 4 sulfur cluster binding	Lipoic acid metabolism, Metabolic pathways	-25.92238	no impact	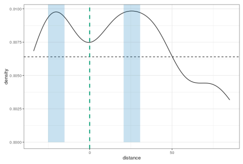
PA14_12230	leuS	leucyl-tRNA synthetase	nucleotide binding, aminoacyl-tRNA ligase activity, ATP binding, tRNA aminoacylation for protein translation, leucine-tRNA ligase activity, leucyl-tRNA aminoacylation, aminoacyl-tRNA editing activity	Aminoacyl-tRNA biosynthesis, Aminoacyl-tRNA biosynthesis	-25.97629	no impact	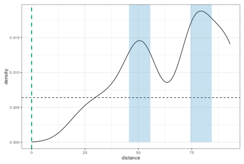
PA14_12260	NA	hypothetical protein			-25.85673	no impact	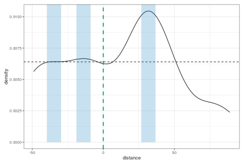
PA14_12300	NA	hypothetical protein	flavin adenine dinucleotide binding		-26.32822	repression	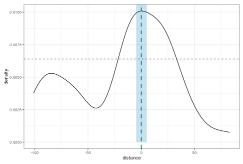
PA14_12310	NA	metalloprotease	metalloendopeptidase activity, rRNA processing		-26.38705	repression	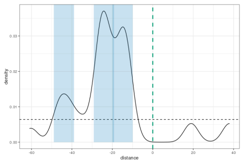
PA14_12550	NA	hypothetical protein	oxygen binding, heme binding		-25.97154	repression	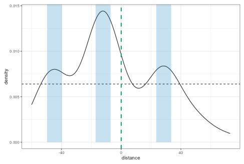
PA14_12730	NA	hypothetical protein	catalytic activity		-27.86752	repression	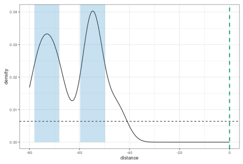

Modeled binding peaks for 1043 predicted targets of RsmA							
PA14 gene ID	gene name	description	GO terms	KEGG pathways	overall affinity score in RT	predicted effect on translation	binding site predictions
PA14_12780	NA	two-component response regulator	regulation of transcription, DNA-templated, phosphorelay signal transduction system, DNA binding	Two-component system	-26.06717	repression	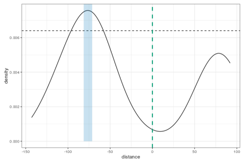
PA14_12810	NA	two-component response regulator	phosphorelay signal transduction system		-26.85600	repression	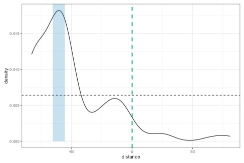
PA14_12900	NA	DNA binding protein	DNA binding		-26.06832	repression	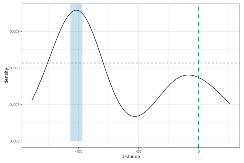
PA14_13040	cioB	CioB, cyanide insensitive terminal oxidase	membrane, oxidation-reduction process	Metabolic pathways, Oxidative phosphorylation, Two-component system	-26.15647	repression	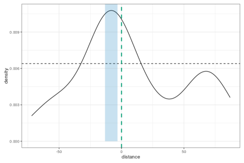
PA14_13060	NA	transcriptional regulator	DNA-binding transcription factor activity, regulation of transcription, DNA-templated, sequence-specific DNA binding, DNA binding		-26.33042	repression	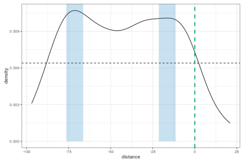
PA14_13190	NA	hypothetical protein	ATP binding		-26.04653	repression	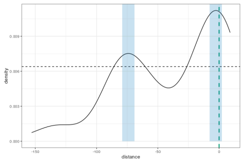
PA14_13240	moaD	molybdopterin converting factor, small subunit			-25.85719	repression	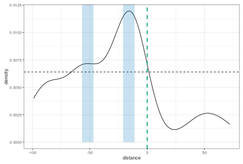
PA14_13320	NA	hypothetical protein			-25.85143	repression	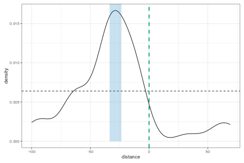
PA14_13350	NA	hypothetical protein			-26.85535	repression	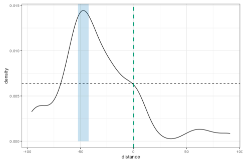
PA14_13360	NA	hypothetical protein			-26.12598	repression	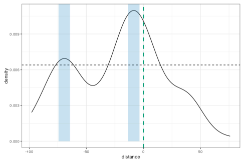

Modeled binding peaks for 1043 predicted targets of RsmA

PA14 gene ID	gene name	description	GO terms	KEGG pathways	overall affinity score in RT	predicted effect on translation	binding site predictions
PA14_13370	NA	hypothetical protein			-26.19700	no impact	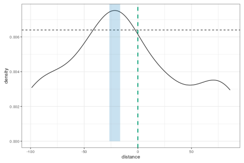
PA14_13490	NA	hypothetical protein	membrane, integral component of membrane		-25.82253	repression	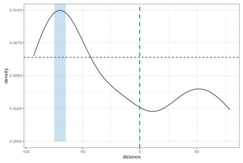
PA14_13580	NA	ABC transporter ATP-binding protein	ATP binding, membrane, glycine betaine transport, ATPase activity	ABC transporters	-26.13390	repression	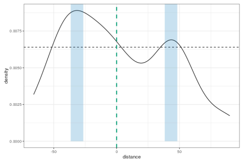
PA14_13590	NA	ABC transporter permease	membrane, transmembrane transport	ABC transporters	-25.95618	repression	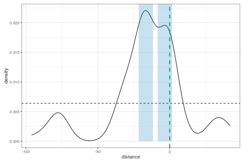
PA14_13620	nhaP	Na+/H+ antiporter NhaP	cation transport, solute:proton antiporter activity, integral component of membrane, transmembrane transport		-25.90977	repression	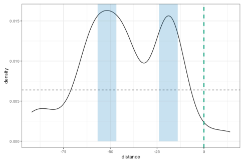
PA14_13690	NA	methyltransferase	methyltransferase activity		-26.80318	repression	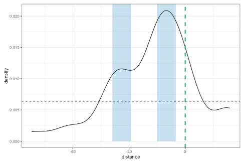
PA14_13850	moaA	molybdenum cofactor biosynthesis protein A	catalytic activity, iron-sulfur cluster binding, Mo-molybdopterin cofactor biosynthetic process, metal ion binding, molybdopterin synthase complex, 4 iron, 4 sulfur cluster binding	Folate biosynthesis, Metabolic pathways, Sulfur relay system	-26.00217	repression	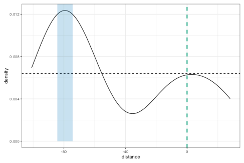
PA14_13870	NA	hypothetical protein			-26.10598	repression	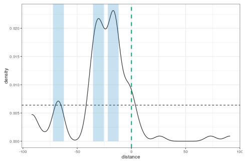
PA14_14100	NA	amino acid-binding protein			-27.06885	repression	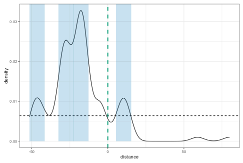
PA14_14520	NA	hypothetical protein			-26.15200	repression	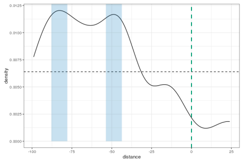

Modeled binding peaks for 1043 predicted targets of RsmA

PA14 gene ID	gene name	description	GO terms	KEGG pathways	overall affinity score in RT	predicted effect on translation	binding site predictions
PA14_14650	secF	preprotein translocase subunit SecF	intracellular protein transport, P-P-bond-hydrolysis-driven protein transmembrane transporter activity	Bacterial secretion system, Protein export	-25.82436	repression	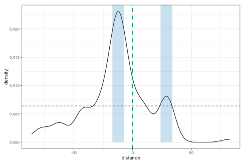
PA14_14750	NA	iron-binding protein IscA	structural molecule activity, iron-sulfur cluster binding, protein maturation by iron-sulfur cluster transfer, iron ion binding		-26.58702	no impact	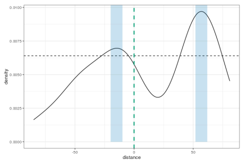
PA14_14810	NA	hypothetical protein	iron-sulfur cluster assembly		-25.97687	repression	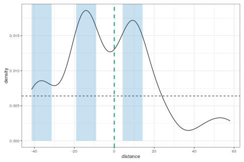
PA14_14910	NA	hypothetical protein	protein binding		-26.73598	repression	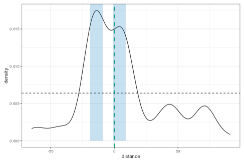
PA14_14975	NA	hypothetical protein			-26.59725	repression	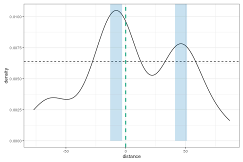
PA14_15070	oprC	outer membrane copper receptor OprC			-26.21452	repression	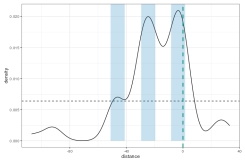
PA14_15090	NA	hypothetical protein			-26.43017	repression	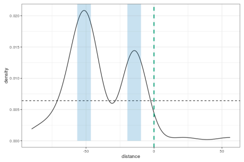
PA14_15120	NA	hypothetical protein			-25.97408	repression	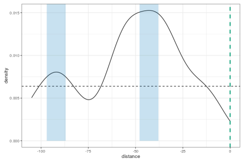
PA14_15290	NA	transcriptional regulator	DNA binding, regulation of transcription, DNA-templated		-27.18934	repression	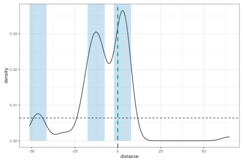
PA14_15430	NA	resolvase, essential for transposition	recombinase activity, DNA binding, DNA recombination		-25.95126	repression	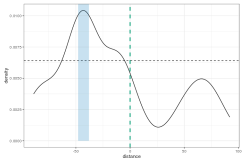

Modeled binding peaks for 1043 predicted targets of RsmA							
PA14 gene ID	gene name	description	GO terms	KEGG pathways	overall affinity score in RT	predicted effect on translation	binding site predictions
PA14_15930	NA	hemolysin	flavin adenine dinucleotide binding		-25.90461	repression	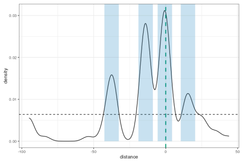
PA14_15990	trmD	tRNA (guanine-N(1)-)-methyltransferase	tRNA processing, tRNA (guanine(37)-N(1))-methyltransferase activity		-26.28788	repression	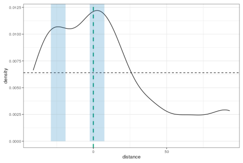
PA14_16030	NA	sodium/hydrogen antiporter	cation transport, solute:proton antiporter activity, integral component of membrane, transmembrane transport, potassium ion transport		-26.51285	repression	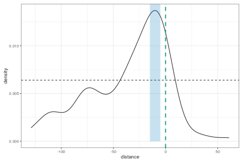
PA14_16130	NA	hypothetical protein	CoA-transferase activity		-26.82606	no impact	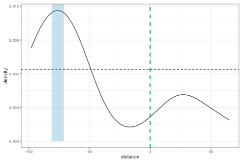
PA14_16140	NA	hypothetical protein			-27.32750	repression	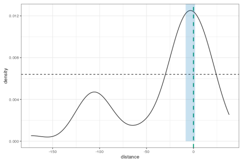
PA14_16150	NA	hypothetical protein			-26.41031	repression	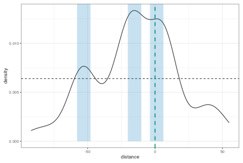
PA14_16160	NA	hypothetical protein			-27.14996	repression	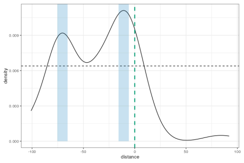
PA14_16180	NA	hypothetical protein			-26.45421	repression	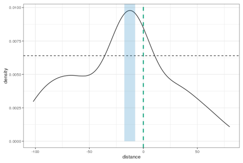
PA14_16200	NA	hypothetical protein			-26.89547	repression	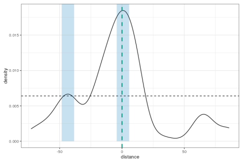
PA14_16250	lasB	elastase LasB	metalloendopeptidase activity, proteolysis, elastin biosynthetic process, elastin catabolic process	Cationic antimicrobial peptide (CAMP) resistance, Quorum sensing	-26.70078	repression	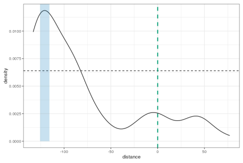

Modeled binding peaks for 1043 predicted targets of RsmA

PA14 gene ID	gene name	description	GO terms	KEGG pathways	overall affinity score in RT	predicted effect on translation	binding site predictions
PA14_16320	NA	peptidyl-prolyl cis-trans isomerase, FkbP-type	peptidyl-prolyl cis-trans isomerase activity		-26.42820	no impact	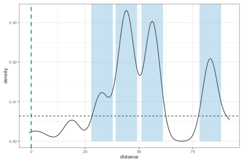
PA14_16410	NA	MFS transporter	integral component of membrane, transmembrane transporter activity, transmembrane transport		-26.17919	repression	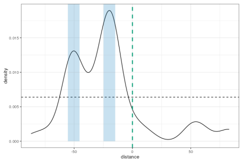
PA14_16450	wspC	methyltransferase	protein binding, S-adenosylmethionine-dependent methyltransferase activity	Two-component system	-26.63684	no impact	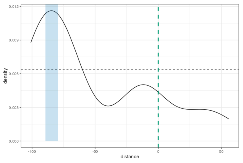
PA14_16560	NA	lipoprotein			-25.85995	repression	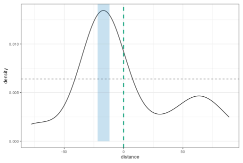
PA14_16600	NA	alpha/beta hydrolase	proteolysis, peptidase activity		-25.81383	repression	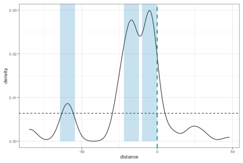
PA14_16620	NA	hypothetical protein			-26.12588	repression	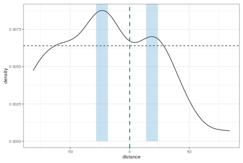
PA14_16630	NA	outer membrane protein, OmpA	cell outer membrane, integral component of membrane		-25.81715	repression	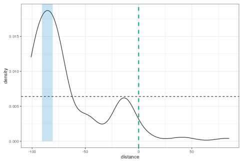
PA14_16640	NA	lipoprotein			-26.92431	repression	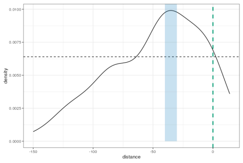
PA14_16660	NA	metal-transporting P-type ATPase	metal ion transport, metal ion binding, cation transport, integral component of membrane, ATPase-coupled cation transmembrane transporter activity, nucleotide binding		-25.80573	no impact	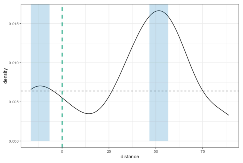
PA14_16780	NA	hypothetical protein	iron ion binding, oxidoreductase activity, acting on paired donors, with incorporation or reduction of molecular oxygen, heme binding, oxidation-reduction process		-26.27789	repression	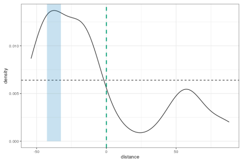

Modeled binding peaks for 1043 predicted targets of RsmA

PA14 gene ID	gene name	description	GO terms	KEGG pathways	overall affinity score in RT	predicted effect on translation	binding site predictions
PA14_16820	NA	efflux transmembrane protein	membrane, transmembrane transporter activity, transmembrane transport		-25.79636	repression	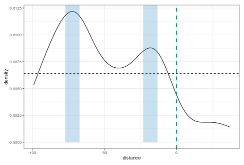
PA14_16870	NA	ABC transporter ATP-binding protein	ATP binding, ATPase activity		-26.23051	repression	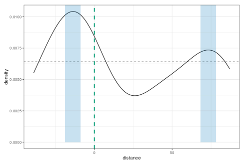
PA14_17000	NA	hypothetical protein			-27.08974	repression	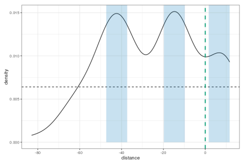
PA14_17010	NA	Na(+)/H(+) exchanger protein	cation transport, solute:proton antiporter activity, integral component of membrane, transmembrane transport		-26.81511	no impact	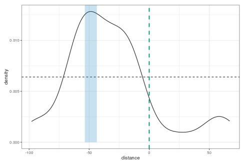
PA14_17040	glnD	PII uridylyl-transferase	nitrogen compound metabolic process, [protein-PII] uridylyltransferase activity, nucleotidyltransferase activity	Two-component system	-25.89944	repression	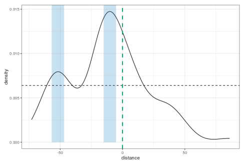
PA14_17060	rpsB	30S ribosomal protein S2	structural constituent of ribosome, ribosome, translation, small ribosomal subunit	Ribosome	-25.95631	no impact	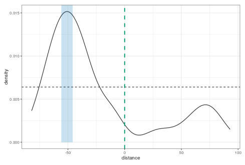
PA14_17100	frr	ribosome recycling factor	translation		-26.66028	no impact	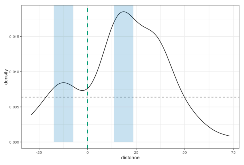
PA14_17110	uppS	UDP pyrophosphate synthetase	transferase activity, transferring alkyl or aryl (other than methyl) groups	Biosynthesis of secondary metabolites, Terpenoid backbone biosynthesis, Terpenoid biosynthesis	-26.95000	repression	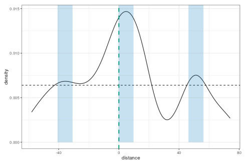
PA14_17290	pyrG	CTP synthetase	CTP synthase activity, CTP biosynthetic process, pyrimidine nucleotide biosynthetic process	Metabolic pathways, Pyrimidine metabolism	-26.11832	repression	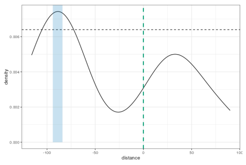
PA14_17380	gfnR	glutathione-dependent formaldehyde neutralization regulator GfnR	DNA-binding transcription factor activity, regulation of transcription, DNA-templated		-25.92163	no impact	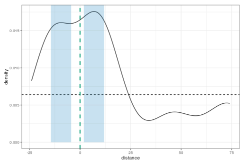

Modeled binding peaks for 1043 predicted targets of RsmA

PA14 gene ID	gene name	description	GO terms	KEGG pathways	overall affinity score in RT	predicted effect on translation	binding site predictions
PA14_17470	NA	hypothetical protein			-26.90654	repression	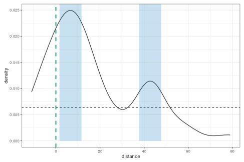
PA14_17530	recA	recombinase A	DNA binding, ATP binding, DNA metabolic process, DNA-dependent ATPase activity, single-stranded DNA binding, DNA repair	Homologous recombination	-26.01173	repression	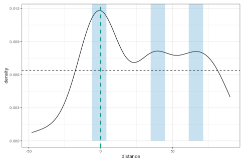
PA14_17550	NA	hypothetical protein			-26.52388	no impact	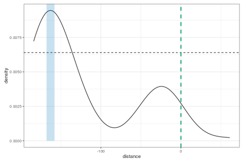
PA14_17570	NA	hypothetical protein			-26.19875	repression	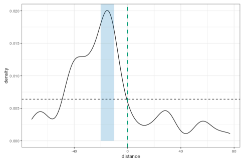
PA14_17620	potC	polyamine transport protein PotC	membrane, transmembrane transport	ABC transporters	-25.91694	repression	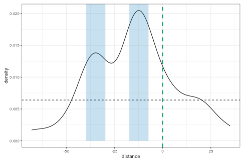
PA14_17700	rpmE2	50S ribosomal protein L31	structural constituent of ribosome, ribosome, translation	Ribosome	-26.80382	repression	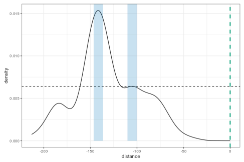
PA14_17780	NA	major facilitator transporter	integral component of membrane, transmembrane transporter activity, transmembrane transport		-25.86545	repression	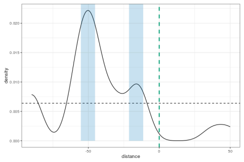
PA14_17850	NA	enoyl-CoA hydratase	catalytic activity		-25.96400	repression	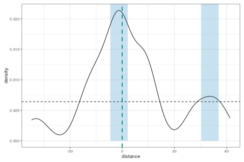
PA14_17860	NA	3-hydroxyacyl-CoA dehydrogenase	oxidation-reduction process, 3-hydroxyacyl-CoA dehydrogenase activity, fatty acid metabolic process, oxidoreductase activity	(4Z,7Z,10Z,13Z,16Z)-docosapentaenoate biosynthesis (6-desaturase), (8<i>E</i>,10<i>E</i>)-dodeca-8,10-dienol biosynthesis, (R)- and (S)-3-hydroxybutanoate biosynthesis (engineered), 2-methylpropene degradation, 3-hydroxypropanoate/4-hydroxybutanate cycle, 4-coumarate degradation (aerobic), 4-coumarate degradation (anaerobic), 4-hydroxybenzoate biosynthesis III (plants), <i>Spodoptera littoralis</i> pheromone biosynthesis, alpha-Linolenic acid metabolism, Aminobenzoate degradation, androstenedione degradation, Benzoate degradation, Benzoate degradation, benzoyl-CoA degradation I (aerobic), beta-Alanine metabolism, Butanoate metabolism, Butanoate metabolism, Caprolactam degradation, Carbon fixation pathways in prokaryotes, cholesterol degradation to androstenedione I (cholesterol oxidase), cholesterol degradation to androstenedione II (cholesterol dehydrogenase), crotonate fermentation (to acetate and cyclohexane carboxylate), docosahexaenoate biosynthesis III (6-desaturase, mammals), fatty acid β-oxidation II (peroxisome), Fatty acid degradation, Fatty acid elongation, fatty acid salvage, fermentation to 2-methylbutanoate, Geraniol degradation, glutaryl-CoA degradation, jasmonic acid biosynthesis, Limonene and pinene degradation, Lysine degradation, Metabolic pathways, methyl <i>tert</i>-butyl ether degradation, methyl ketone biosynthesis (engineered), Microbial metabolism in diverse environments, Phenylacetic acid aerobic catabolism, Phenylalanine metabolism, Phenylalanine metabolism, Propanoate metabolism, pyruvate fermentation to butanol I, pyruvate fermentation to butanol II (engineered), pyruvate fermentation to hexanol (engineered), Toluene degradation, Tryptophan	-25.84608	repression	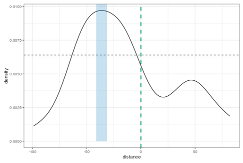

Modeled binding peaks for 1043 predicted targets of RsmA							
PA14 gene ID	gene name	description	GO terms	KEGG pathways	overall affinity score in RT	predicted effect on translation	binding site predictions
				metabolism, unsaturated, even numbered fatty acid β-oxidation, Valine, leucine and isoleucine degradation			
PA14_17920	glpM	membrane protein GlpM			-26.03977	no impact	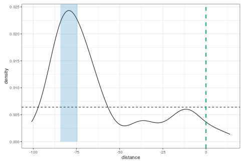
PA14_18040	NA	hypothetical protein			-26.50649	no impact	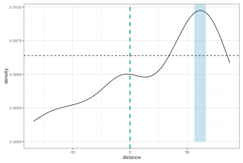
PA14_18140	mmsB	3-hydroxyisobutyrate dehydrogenase	oxidation-reduction process, oxidoreductase activity, NADP binding, NAD binding, 3-hydroxyisobutyrate dehydrogenase activity	Metabolic pathways, Valine, leucine and isoleucine degradation, Valine, leucine and isoleucine degradation	-26.46242	repression	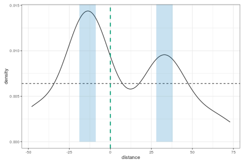
PA14_18200	NA	LysR family transcriptional regulator	DNA-binding transcription factor activity, regulation of transcription, DNA-templated		-27.15498	repression	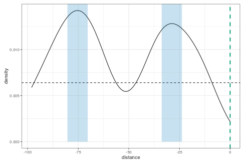
PA14_18250	NA	phosphotransferase system enzyme I	phosphoenolpyruvate-dependent sugar phosphotransferase system, transferase activity, transferring phosphorus-containing groups, phosphorylation, catalytic activity	Fructose and mannose metabolism, Metabolic pathways, Microbial metabolism in diverse environments, Phosphotransferase system (PTS)	-26.25190	no impact	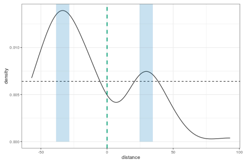
PA14_18450	algI	alginate o-acetyltransferase AlgI	alginic acid biosynthetic process		-25.90706	no impact	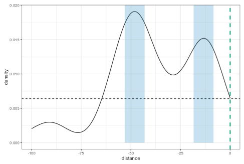
PA14_18565	alg8	alginate biosynthesis protein Alg8	alginic acid biosynthetic process	Alginate biosynthesis, Fructose and mannose metabolism	-25.79661	repression	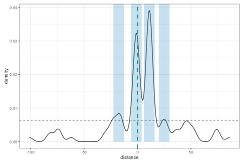
PA14_18580	algD	GDP-mannose 6-dehydrogenase AlgD	oxidation-reduction process, oxidoreductase activity, acting on the CH-OH group of donors, NAD or NADP as acceptor, alginic acid biosynthetic process, NAD binding, GDP-mannose 6-dehydrogenase activity, biofilm formation	Amino sugar and nucleotide sugar metabolism, Fructose and mannose metabolism, Two-component system	-26.19408	repression	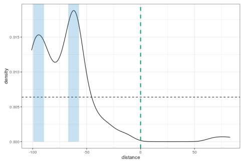
PA14_18620	NA	hypothetical protein			-26.83607	repression	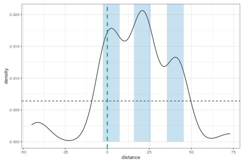
PA14_18760	NA	RND efflux membrane fusion protein	membrane, transmembrane transporter activity, transmembrane transport		-26.27402	repression	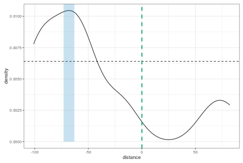

Modeled binding peaks for 1043 predicted targets of RsmA

PA14 gene ID	gene name	description	GO terms	KEGG pathways	overall affinity score in RT	predicted effect on translation	binding site predictions
PA14_18830	NA	adenylosuccinate lyase	N6-(1,2-dicarboxyethyl)AMP AMP-lyase (fumarate-forming) activity, purine ribonucleotide biosynthetic process, catalytic activity	adenosine ribonucleotides <i>de novo</i> biosynthesis, Alanine, aspartate and glutamate metabolism, Alanine, aspartate and glutamate metabolism, Biosynthesis of antibiotics, Biosynthesis of secondary metabolites, inosine-5'-phosphate biosynthesis I, inosine-5'-phosphate biosynthesis II, inosine-5'-phosphate biosynthesis III, Metabolic pathways, Purine metabolism, Purine metabolism	-26.29362	no impact	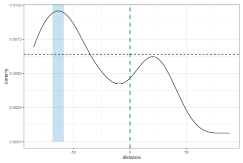
PA14_18850	NA	adenylosuccinate lyase	catalytic activity	adenosine ribonucleotides <i>de novo</i> biosynthesis, Alanine, aspartate and glutamate metabolism, Alanine, aspartate and glutamate metabolism, Biosynthesis of antibiotics, Biosynthesis of secondary metabolites, inosine-5'-phosphate biosynthesis I, inosine-5'-phosphate biosynthesis II, inosine-5'-phosphate biosynthesis III, Metabolic pathways, Purine metabolism, Purine metabolism	-27.01238	repression	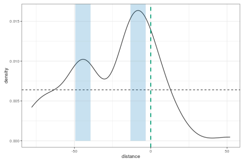
PA14_18860	NA	hypothetical protein			-25.80204	repression	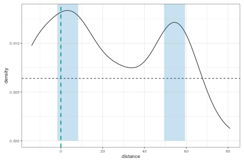
PA14_18960	tli5a	type VI secretion lipase immunity Tli5A			-27.15836	repression	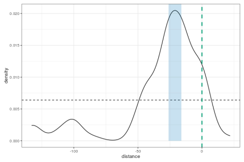
PA14_18970	pldA	phospholipase D	catalytic activity		-25.89773	repression	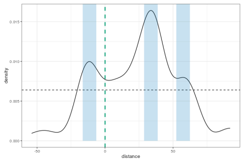
PA14_18985	vgrG4b	VgrG4b			-25.94737	repression	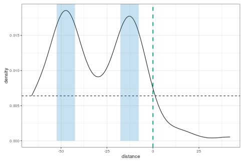
PA14_19020	tse3	Tse3			-26.51496	repression	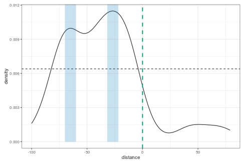
PA14_19030	NA	hypothetical protein			-26.85621	repression	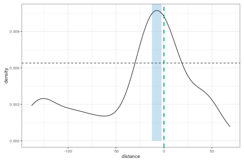
PA14_19130	rhlI	autoinducer synthesis protein RhlI	transferase activity	autoinducer AI-1 biosynthesis, Biofilm formation - Pseudomonas aeruginosa, Cysteine and methionine metabolism, Cysteine and methionine metabolism, Metabolic pathways, Quorum sensing, Two-component system	-26.37784	repression	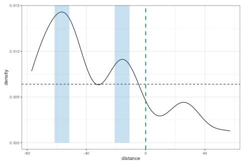
PA14_19170	NA	hypothetical protein			-25.84970	no impact	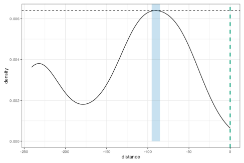

Modeled binding peaks for 1043 predicted targets of RsmA							
PA14 gene ID	gene name	description	GO terms	KEGG pathways	overall affinity score in RT	predicted effect on translation	binding site predictions
PA14_19230	NA	hypothetical protein	membrane, transmembrane transport		-25.87870	repression	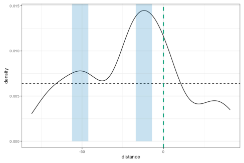
PA14_19360	NA	GNAT family acetyltransferase	ATP binding, metal ion binding, N-acetyltransferase activity		-26.85104	repression	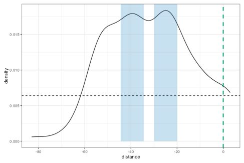
PA14_19370	NA	asparagine synthetase	asparagine synthase (glutamine-hydrolyzing) activity, asparagine biosynthetic process	Alanine, aspartate and glutamate metabolism, Biosynthesis of secondary metabolites, Metabolic pathways	-26.61743	repression	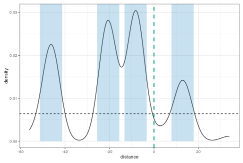
PA14_19380	NA	transcriptional regulator	DNA-binding transcription factor activity, regulation of transcription, DNA-templated		-27.06465	repression	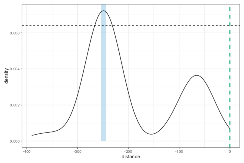
PA14_19490	lsfA	1-Cys peroxiredoxin LsfA	oxidation-reduction process, cell redox homeostasis, antioxidant activity, oxidoreductase activity, peroxiredoxin activity, response to hydrogen peroxide, pathogenesis		-25.81317	no impact	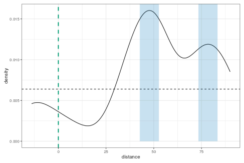
PA14_19590	NA	molybdopterin-binding protein	molybdate ion transport		-26.70072	no impact	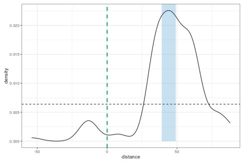
PA14_19620	folX	D-erythro-7,8-dihydroneopterin triphosphate 2'-epimerase	dihydroneopterin aldolase activity, folic acid-containing compound metabolic process, tetrahydrobiopterin biosynthetic process		-26.43002	no impact	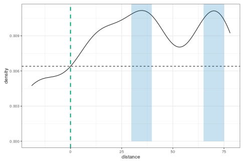
PA14_19670	NA	LysR family transcriptional regulator	DNA-binding transcription factor activity, regulation of transcription, DNA-templated		-27.29182	repression	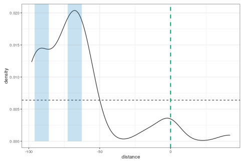
PA14_19720	NA	hypothetical protein			-26.26229	repression	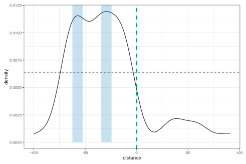
PA14_19860	NA	hypothetical protein			-26.01294	repression	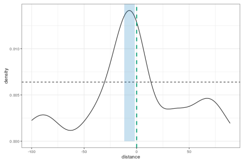

Modeled binding peaks for 1043 predicted targets of RsmA

PA14 gene ID	gene name	description	GO terms	KEGG pathways	overall affinity score in RT	predicted effect on translation	binding site predictions
PA14_19870	ldh	leucine dehydrogenase	cellular amino acid metabolic process, oxidoreductase activity, oxidation-reduction process, oxidoreductase activity, acting on the CH-NH2 group of donors, NAD or NADP as acceptor	Biosynthesis of antibiotics, Biosynthesis of secondary metabolites, Metabolic pathways, Valine, leucine and isoleucine biosynthesis, Valine, leucine and isoleucine degradation	-25.99024	no impact	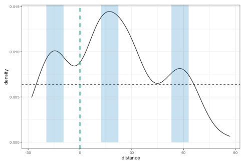
PA14_19910	NA	pyruvate dehydrogenase E1 component, beta chain	catalytic activity	Biosynthesis of antibiotics, Biosynthesis of secondary metabolites, Carbon metabolism, Citrate cycle (TCA cycle), Glycolysis / Gluconeogenesis, Metabolic pathways, Microbial metabolism in diverse environments, Pyruvate metabolism	-26.16541	repression	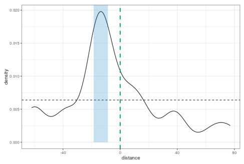
PA14_19950	NA	hypothetical protein			-25.82443	no impact	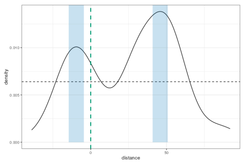
PA14_20010	hasR	heme uptake outer membrane receptor HasR	outer membrane, transmembrane transporter activity, transmembrane transport, heme transporter activity, heme transport, heme binding		-26.86567	no impact	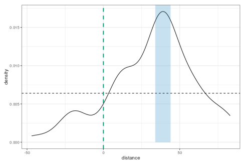
PA14_20120	NA	hypothetical protein			-25.76839	repression	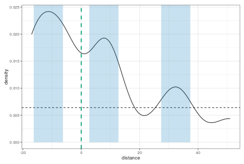
PA14_20130	NA	LysR family transcriptional regulator	DNA-binding transcription factor activity, regulation of transcription, DNA-templated		-26.42456	repression	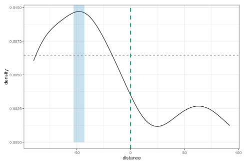
PA14_20190	nosD	copper ABC transporter periplasmic substrate-binding protein			-26.16724	repression	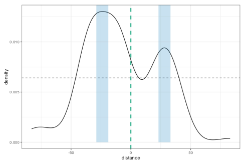
PA14_20280	NA	hypothetical protein			-25.88817	no impact	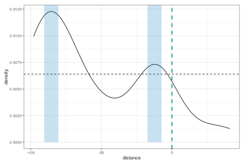
PA14_20440	phnN	phosphonate transport ATP-binding protein	5-phosphoribose 1-diphosphate biosynthetic process, ribose 1,5-bisphosphate phosphokinase activity	Pentose phosphate pathway	-26.32165	no impact	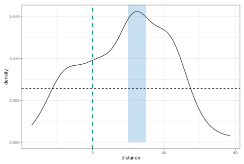
PA14_20460	NA	hypothetical protein			-26.73825	repression	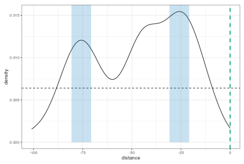

Modeled binding peaks for 1043 predicted targets of RsmA

PA14 gene ID	gene name	description	GO terms	KEGG pathways	overall affinity score in RT	predicted effect on translation	binding site predictions
PA14_20470	NA	hypothetical protein			-26.80276	repression	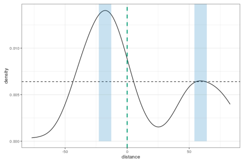
PA14_20560	amiE	acylamide amidohydrolase	nitrogen compound metabolic process, amidase activity	Aminobenzoate degradation, Arginine and proline metabolism, Microbial metabolism in diverse environments, Phenylalanine metabolism, Styrene degradation, Tryptophan metabolism	-26.36225	repression	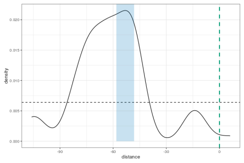
PA14_20580	amiC	aliphatic amidase expression-regulating protein	amino acid transport, negative regulation of hydrolase activity, amide binding, regulation of cellular amide catabolic process	ABC transporters, Quorum sensing	-26.62535	repression	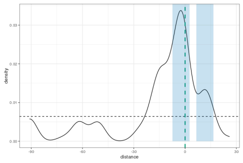
PA14_20670	NA	glutamine synthetase	catalytic activity, glutamate-ammonia ligase activity, nitrogen compound metabolic process	Alanine, aspartate and glutamate metabolism, Alanine, aspartate and glutamate metabolism, ammonia assimilation cycle I, ammonia assimilation cycle II, Arginine biosynthesis, Arginine biosynthesis, Biosynthesis of amino acids, Glyoxylate and dicarboxylate metabolism, Glyoxylate and dicarboxylate metabolism, L-glutamine biosynthesis III, Metabolic pathways, Microbial metabolism in diverse environments, nitrate reduction II (assimilatory), nitrate reduction V (assimilatory), Nitrogen metabolism, Nitrogen metabolism, Two-component system	-25.86110	repression	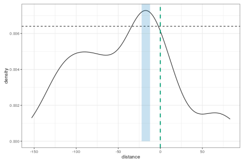
PA14_20770	NA	hypothetical protein			-25.93219	no impact	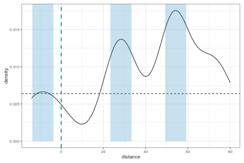
PA14_20800	NA	histidine phosphotransfer domain-containing protein	phosphorelay signal transduction system, histidine phosphotransfer kinase activity, negative regulation of single-species biofilm formation, positive regulation of chemotaxis, regulation of cell motility	Biofilm formation - Pseudomonas aeruginosa, Two-component system	-26.42412	repression	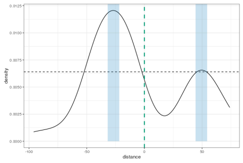
PA14_20940	NA	acyl carrier protein			-27.31959	repression	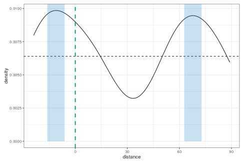
PA14_21020	NA	non-ribosomal peptide synthetase	biosynthetic process, hydrolase activity, acting on ester bonds, phosphopantetheine binding, catalytic activity		-26.91402	repression	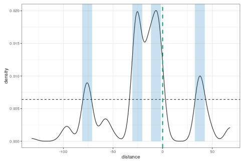
PA14_21030	NA	ATP-dependent Clp protease proteolytic subunit	serine-type endopeptidase activity, proteolysis		-27.05512	repression	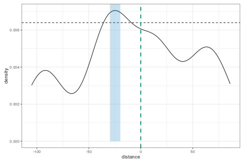
PA14_21210	NA	hypothetical protein	integral component of membrane, catalytic activity, sulfuric ester hydrolase activity	Cationic antimicrobial peptide (CAMP) resistance	-26.12260	repression	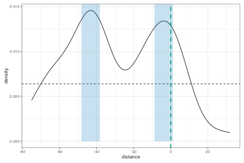

Modeled binding peaks for 1043 predicted targets of RsmA

PA14 gene ID	gene name	description	GO terms	KEGG pathways	overall affinity score in RT	predicted effect on translation	binding site predictions
PA14_21300	NA	MFS transporte	integral component of plasma membrane, transmembrane transport		-25.82375	repression	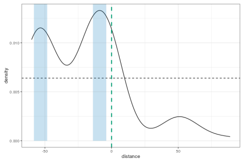
PA14_21310	phaJ1	hypothetical protein	fatty acid synthase activity, fatty acid synthase complex, fatty acid biosynthetic process, oxidation-reduction process		-25.91942	repression	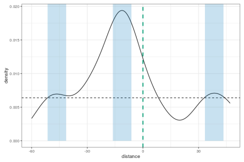
PA14_21450	NA	hypothetical protein			-25.94737	repression	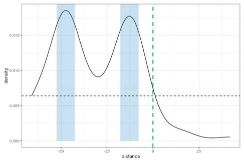
PA14_21530	NA	ankyrin domain-containing protein	protein binding		-25.79932	repression	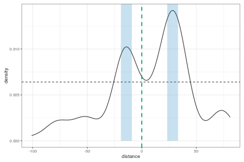
PA14_21580	NA	hypothetical protein			-26.59585	repression	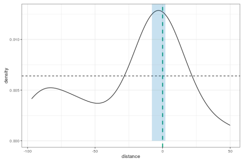
PA14_21600	NA	hypothetical protein			-25.94506	repression	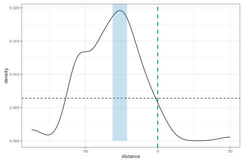
PA14_21610	oprO	pyrophosphate-specific outer membrane porin OprO precursor			-26.83569	repression	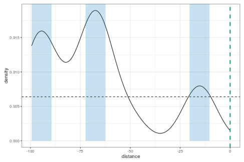
PA14_21620	oprP	phosphate-specific outer membrane porin OprP precursor			-26.48652	repression	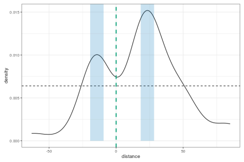
PA14_21630	NA	hypothetical protein			-26.15904	repression	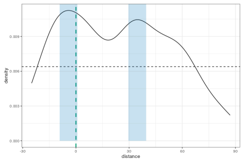
PA14_21670	NA	hypothetical protein			-25.88098	no impact	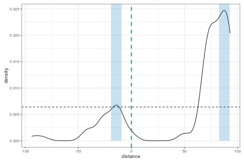

Modeled binding peaks for 1043 predicted targets of RsmA

PA14 gene ID	gene name	description	GO terms	KEGG pathways	overall affinity score in RT	predicted effect on translation	binding site predictions
PA14_21900	NA	HAD-superfamily hydrolase			-26.37002	no impact	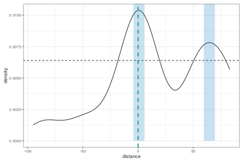
PA14_21960	NA	hypothetical protein			-25.82256	repression	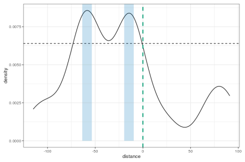
PA14_21970	NA	transcriptional regulator	DNA binding, regulation of transcription, DNA-templated, DNA-binding transcription factor activity		-26.32323	repression	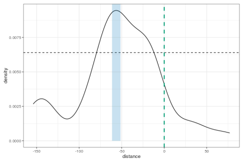
PA14_21980	NA	hypothetical protein	N-acetyltransferase activity		-25.82838	repression	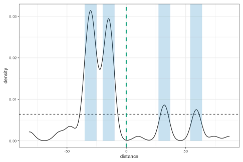
PA14_22320	NA	hypothetical protein			-26.31297	repression	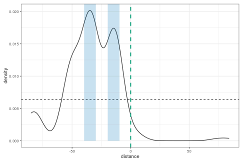
PA14_22330	NA	glycine betaine-binding protein	transmembrane transporter activity, ATP-binding cassette (ABC) transporter complex, transmembrane transport	ABC transporters	-26.57926	repression	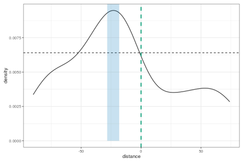
PA14_22420	NA	hypothetical protein			-26.74312	repression	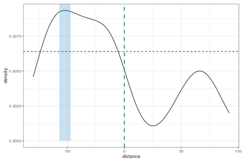
PA14_22440	NA	ABC transporter ATP-binding protein/permease	ATP binding, ATPase activity, integral component of membrane, ATPase-coupled transmembrane transporter activity, transmembrane transport	ABC transporters	-25.98806	repression	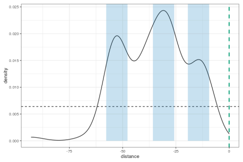
PA14_22470	NA	LysR family transcriptional regulator	DNA-binding transcription factor activity, regulation of transcription, DNA-templated		-26.47356	repression	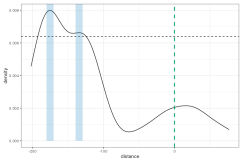
PA14_22570	csaA	CsaA protein	tRNA binding		-26.01327	repression	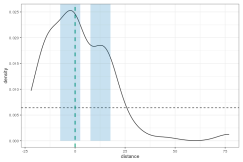

Modeled binding peaks for 1043 predicted targets of RsmA

PA14 gene ID	gene name	description	GO terms	KEGG pathways	overall affinity score in RT	predicted effect on translation	binding site predictions
PA14_22650	NA	ABC transporter			-25.78261	repression	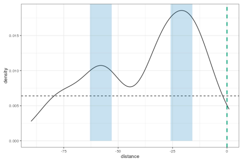
PA14_22710	NA	hypothetical protein	oxidoreductase activity		-25.85693	repression	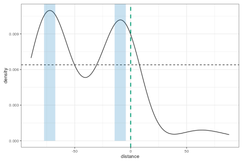
PA14_23050	NA	hypothetical protein	catalytic activity, carbohydrate metabolic process, carbohydrate binding, isomerase activity		-26.04791	repression	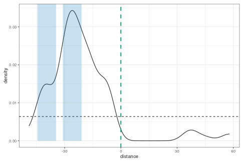
PA14_23080	pgl	6-phosphogluconolactonase	carbohydrate metabolic process, pentose-phosphate shunt, 6-phosphogluconolactonase activity	Amino sugar and nucleotide sugar metabolism, Biosynthesis of antibiotics, Biosynthesis of secondary metabolites, Carbon metabolism, chitin degradation I (archaea), chitin derivatives degradation, Entner-Doudoroff pathway I, Metabolic pathways, Microbial metabolism in diverse environments, Pentose phosphate pathway, Pentose phosphate pathway, UDP-<i>N</i>-acetyl-D-galactosamine biosynthesis II	-25.86417	repression	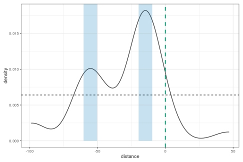
PA14_23120	NA	hypothetical protein			-26.39796	no impact	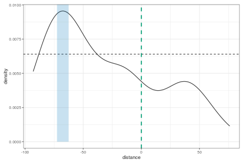
PA14_23260	gyrA	DNA gyrase subunit A	DNA binding, DNA topoisomerase type II (ATP-hydrolyzing) activity, ATP binding, DNA topological change, chromosome, DNA topoisomerase activity, DNA metabolic process		-26.47014	repression	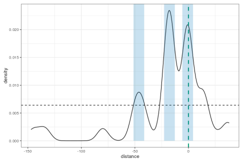
PA14_23280	pheA	chorismate mutase	prephenate dehydratase activity, L-phenylalanine biosynthetic process, chorismate metabolic process, chorismate mutase activity, cytoplasm	Biosynthesis of amino acids, Biosynthesis of antibiotics, Biosynthesis of secondary metabolites, Metabolic pathways, Phenylalanine, tyrosine and tryptophan biosynthesis	-25.96976	repression	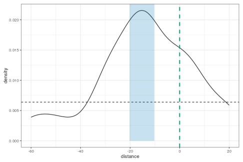
PA14_23340	ihfB	integration host factor subunit beta	DNA binding, chromosome, DNA recombination, regulation of transcription, DNA-templated		-27.11791	no impact	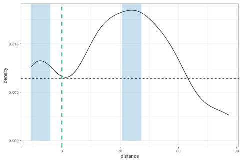
PA14_23370	orfK	UDP-N-acetylglucosamine 2-epimerase	UDP-N-acetylglucosamine 2-epimerase activity	Amino sugar and nucleotide sugar metabolism, Metabolic pathways	-27.07920	no impact	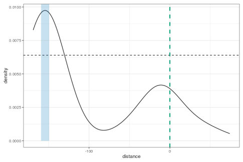
PA14_23450	orfM	NAD dependent epimerase/dehydratase	catalytic activity, coenzyme binding		-25.88524	no impact	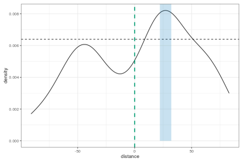

Modeled binding peaks for 1043 predicted targets of RsmA

PA14 gene ID	gene name	description	GO terms	KEGG pathways	overall affinity score in RT	predicted effect on translation	binding site predictions
PA14_23470	wbpM	nucleotide sugar epimerase/dehydratase WbpM			-27.51562	repression	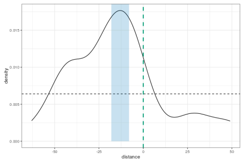
PA14_23540	act	transcriptional regulator	DNA-binding transcription factor activity, regulation of transcription, DNA-templated		-25.89344	repression	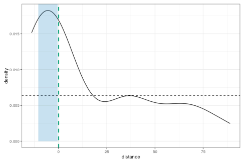
PA14_23630	NA	hypothetical protein			-26.12185	no impact	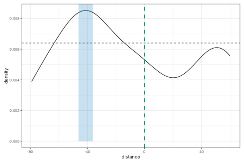
PA14_23700	NA	LysR family transcriptional regulator	DNA-binding transcription factor activity, regulation of transcription, DNA-templated		-26.38005	repression	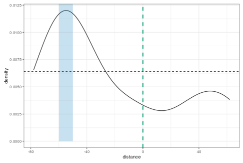
PA14_23830	fimV	pilus assembly protein	protein binding		-26.57988	repression	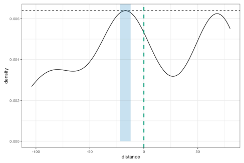
PA14_23890	NA	hypothetical protein	peptidoglycan binding		-26.78843	repression	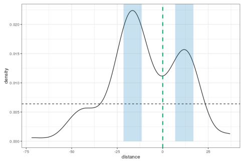
PA14_23920	purF	amidophosphoribosyltransferase	amidophosphoribosyltransferase activity, purine nucleobase biosynthetic process, nucleoside metabolic process	Alanine, aspartate and glutamate metabolism, Biosynthesis of antibiotics, Biosynthesis of secondary metabolites, Metabolic pathways, Purine metabolism	-26.02310	repression	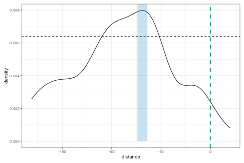
PA14_23930	metZ	O-succinylhomoserine sulfhydrylase	transsulfuration, pyridoxal phosphate binding, homocysteine biosynthetic process, catalytic activity, methionine biosynthetic process	Cysteine and methionine metabolism, Metabolic pathways, Sulfur metabolism	-26.19706	repression	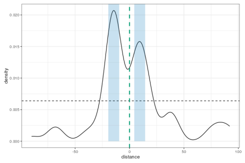
PA14_24040	xcpU	general secretion pathway outer membrane protein H precursor	protein secretion by the type II secretion system, type II protein secretion system complex	Bacterial secretion system	-25.90969	repression	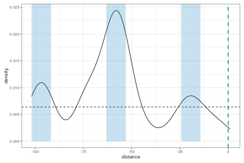
PA14_24070	xcpX	general secretion pathway protein K	protein secretion, integral component of membrane, type II protein secretion system complex	Bacterial secretion system	-25.93777	repression	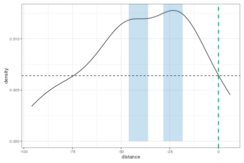

Modeled binding peaks for 1043 predicted targets of RsmA							
PA14 gene ID	gene name	description	GO terms	KEGG pathways	overall affinity score in RT	predicted effect on translation	binding site predictions
PA14_24210	NA	hypothetical protein			-26.41236	no impact	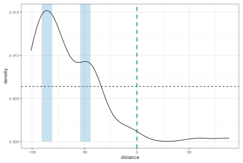
PA14_24440	NA	lipoprotein			-26.75498	repression	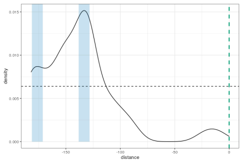
PA14_24490	pelB	hypothetical protein	protein binding, single-species biofilm formation, single-species biofilm formation	Biofilm formation - Pseudomonas aeruginosa	-25.84737	repression	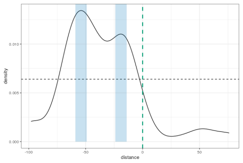
PA14_24580	NA	hypothetical protein	DNA binding		-26.52792	repression	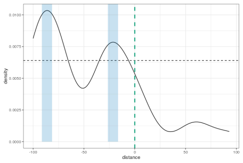
PA14_24590	NA	hypothetical protein	DNA binding		-26.60951	repression	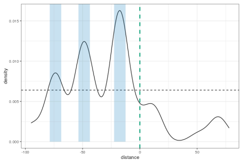
PA14_24700	NA	hypothetical protein			-25.92705	repression	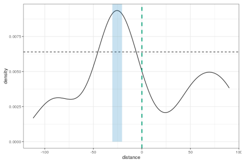
PA14_24840	NA	transcriptional regulator	DNA binding		-26.79541	repression	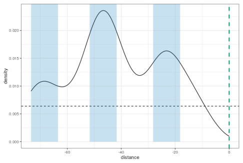
PA14_24890	mobA	molybdopterin-guanine dinucleotide biosynthesis protein MobA	catalytic activity, Mo-molybdopterin cofactor biosynthetic process		-26.07043	no impact	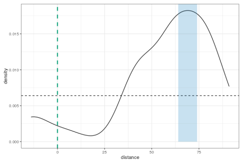
PA14_24940	NA	hypothetical protein	flavin adenine dinucleotide binding, FAD binding, catalytic activity, oxidoreductase activity, oxidation-reduction process	Ether lipid metabolism, Metabolic pathways	-26.44050	repression	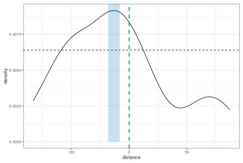
PA14_24970	NA	lipid kinase	lipid kinase activity, metal ion binding, kinase activity, NAD+ kinase activity		-27.05651	repression	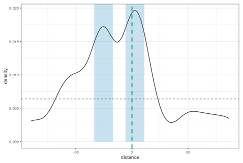

Modeled binding peaks for 1043 predicted targets of RsmA

PA14 gene ID	gene name	description	GO terms	KEGG pathways	overall affinity score in RT	predicted effect on translation	binding site predictions
PA14_25030	NA	hypothetical protein			-25.81730	repression	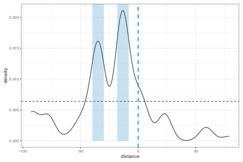
PA14_25040	NA	hypothetical protein			-26.24908	no impact	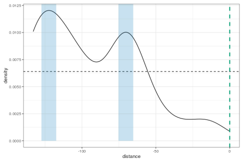
PA14_25060	NA	hypothetical protein			-26.56518	repression	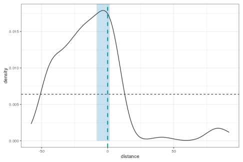
PA14_25080	fadB	multifunctional fatty acid oxidation complex subunit alpha	3-hydroxyacyl-CoA dehydrogenase activity, dodecenoyl-CoA delta-isomerase activity, enoyl-CoA hydratase activity, 3-hydroxybutyryl-CoA epimerase activity, fatty acid catabolic process, fatty acid beta-oxidation multienzyme complex, oxidation-reduction process, catalytic activity, fatty acid metabolic process, oxidoreductase activity	Benzoate degradation, beta-Alanine metabolism, Biosynthesis of antibiotics, Biosynthesis of secondary metabolites, Biosynthesis of unsaturated fatty acids, Butanoate metabolism, Caprolactam degradation, Carbon metabolism, Fatty acid degradation, Fatty acid metabolism, Geraniol degradation, Limonene and pinene degradation, Lysine degradation, Metabolic pathways, Microbial metabolism in diverse environments, Propanoate metabolism, Tryptophan metabolism, Valine, leucine and isoleucine degradation	-26.35696	repression	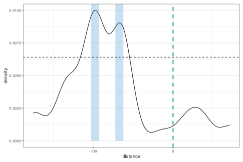
PA14_25090	fadA	3-ketoacyl-CoA thiolase	transferase activity, transferring acyl groups other than amino-acyl groups, acetyl-CoA C-acyltransferase activity, cytoplasm, fatty acid metabolic process, lipid catabolic process, catalytic activity	(4Z,7Z,10Z,13Z,16Z)-docosapentaenoate biosynthesis (6-desaturase), (8<i>E</i>,10<i>E</i>)-dodeca-8,10-dienol biosynthesis, 10-<i>cis</i>-heptadecenoyl-CoA degradation (yeast), 10-<i>trans</i>-heptadecenoyl-CoA degradation (MFE-dependent, yeast), 10-<i>trans</i>-heptadecenoyl-CoA degradation (reductase-dependent, yeast), 4-ethylphenol degradation (anaerobic), 4-hydroxybenzoate biosynthesis III (plants), 4-oxopentanoate degradation, 9-<i>cis</i>, 11-<i>trans</i>-octadecadienoyl-CoA degradation (isomerase-dependent, yeast), alpha-Linolenic acid metabolism, alpha-Linolenic acid metabolism, androstenedione degradation, Benzoate degradation, Benzoate degradation, Biosynthesis of antibiotics, Biosynthesis of secondary metabolites, cholesterol degradation to androstenedione I (cholesterol oxidase), cholesterol degradation to androstenedione II (cholesterol dehydrogenase), docosahexaenoate biosynthesis III (6-desaturase, mammals), Ethylbenzene degradation, fatty acid β-oxidation (peroxisome, yeast), fatty acid β-oxidation II (peroxisome), Fatty acid degradation, Fatty acid degradation, Fatty acid elongation, Fatty acid metabolism, fatty acid salvage, fermentation to 2-methylbutanoate, Geraniol degradation, Geraniol degradation, jasmonic acid biosynthesis, Metabolic pathways, Microbial metabolism in diverse environments, pyruvate fermentation to hexanol (engineered), sitosterol degradation to androstenedione, Valine, leucine and isoleucine degradation, Valine, leucine and isoleucine degradation	-25.81502	no impact	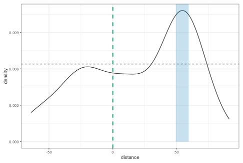
PA14_25210	NA	5'-methylthioadenosine phosphorylase	S-methyl-5-thioadenosine phosphorylase activity, catalytic activity, nucleoside metabolic process, transferase activity, transferring pentosyl groups	Cysteine and methionine metabolism, Metabolic pathways	-25.89917	no impact	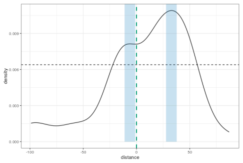
PA14_25270	aroP1	aromatic amino acid transport protein AroP1	amino acid transport, integral component of membrane, transmembrane transport, membrane, transmembrane transporter activity		-26.17613	repression	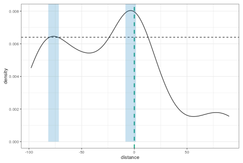
PA14_25305	nqrB	Na(+)-translocating NADH-quinone reductase subunit B	FMN binding, integral component of membrane, oxidoreductase activity, acting on NAD(P)H, quinone or similar compound as acceptor, respiratory electron transport chain, membrane, transmembrane transport		-26.26475	repression	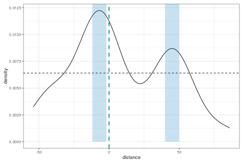
PA14_25340	nqrE	Na(+)-translocating NADH-quinone reductase subunit E	Gram-negative-bacterium-type cell wall, integral component of membrane, oxidoreductase activity, acting on NAD(P)H, quinone or similar compound as acceptor, respiratory electron transport chain, oxidation-reduction process, membrane		-26.42902	repression	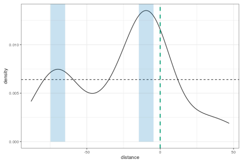

Modeled binding peaks for 1043 predicted targets of RsmA

PA14 gene ID	gene name	description	GO terms	KEGG pathways	overall affinity score in RT	predicted effect on translation	binding site predictions
PA14_25390	sth	soluble pyridine nucleotide transhydrogenase	cell redox homeostasis, oxidation-reduction process, oxidoreductase activity, flavin adenine dinucleotide binding, electron transfer activity, NAD(P)+ transhydrogenase (B-specific) activity	Metabolic pathways, Nicotinate and nicotinamide metabolism	-26.72353	no impact	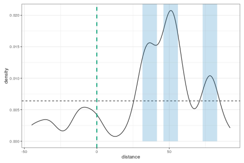
PA14_25420	NA	hypothetical protein	cyclic-di-GMP binding		-26.54937	no impact	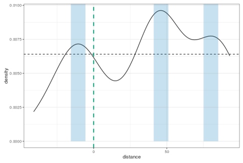
PA14_25480	NA	competence protein	integral component of membrane, establishment of competence for transformation		-26.63862	repression	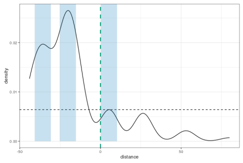
PA14_25520	NA	hypothetical protein			-25.89708	repression	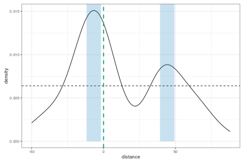
PA14_25830	NA	hypothetical protein			-26.41059	no impact	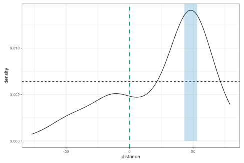
PA14_25840	NA	electron transfer flavoprotein-ubiquinone oxidoreductase			-25.80475	repression	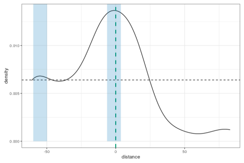
PA14_26000	NA	magnesium chelatase			-25.95272	repression	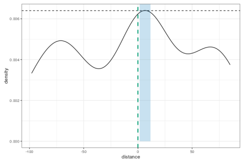
PA14_26140	NA	transcriptional regulator	DNA binding		-27.06976	no impact	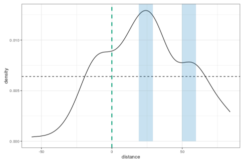
PA14_26165	NA	hypothetical protein			-27.31012	no impact	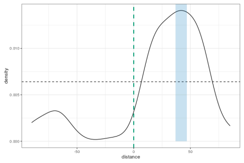
PA14_26210	hisP	histidine transport	ATP binding, ATPase activity, amino acid transmembrane transport, ATPase-coupled amino acid transmembrane transporter activity	ABC transporters	-26.58451	no impact	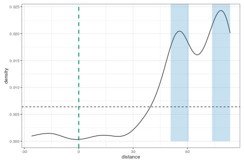

Modeled binding peaks for 1043 predicted targets of RsmA

PA14 gene ID	gene name	description	GO terms	KEGG pathways	overall affinity score in RT	predicted effect on translation	binding site predictions
PA14_26240	hisJ	periplasmic histidine-binding protein HisJ			-25.81831	repression	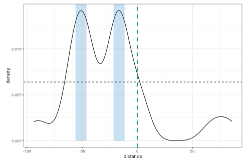
PA14_26510	cobI	precorrin-2 C(20)-methyltransferase	S-adenosylmethionine-dependent methyltransferase activity, cobalamin biosynthetic process, precorrin-2 C20-methyltransferase activity, methyltransferase activity	Metabolic pathways, Porphyrin and chlorophyll metabolism	-26.13547	repression	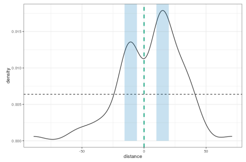
PA14_26690	NA	enoyl-CoA hydratase/isomerase	catalytic activity	Geraniol degradation	-26.08283	repression	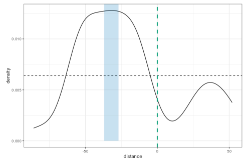
PA14_26700	NA	acyl-CoA dehydrogenase	oxidoreductase activity, acting on the CH-CH group of donors, oxidation-reduction process, flavin adenine dinucleotide binding, acyl-CoA dehydrogenase activity, citronellyl-CoA dehydrogenase activity, terpene catabolic process	Geraniol degradation	-26.38646	repression	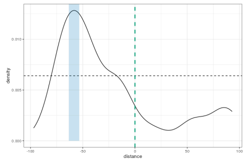
PA14_26760	NA	transcriptional regulator	DNA binding		-26.41044	repression	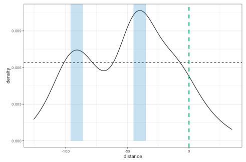
PA14_26850	NA	hypothetical protein			-26.42383	repression	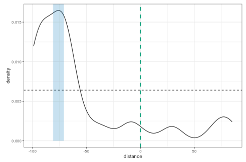
PA14_26880	bvlR	BvlR	DNA-binding transcription factor activity, regulation of transcription, DNA-templated, pathogenesis		-27.42778	repression	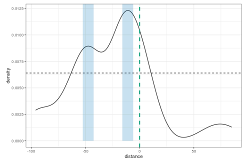
PA14_26940	NA	hypothetical protein			-26.56985	no impact	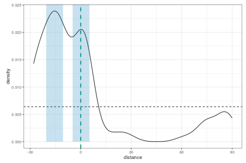
PA14_27370	NA	ATP-dependent RNA helicase	nucleic acid binding, ATP binding, ribosomal large subunit assembly, RNA helicase activity, cellular response to cold	RNA degradation	-26.15910	repression	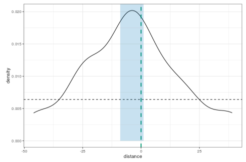
PA14_27400	NA	LysR family transcriptional regulator	DNA-binding transcription factor activity, regulation of transcription, DNA-templated		-26.60955	repression	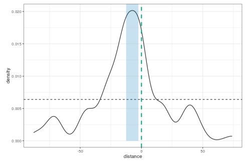

Modeled binding peaks for 1043 predicted targets of RsmA

PA14 gene ID	gene name	description	GO terms	KEGG pathways	overall affinity score in RT	predicted effect on translation	binding site predictions
PA14_27510	NA	methionine sulfoxide reductase B	peptide-methionine (R)-S-oxide reductase activity, oxidation-reduction process, pathogenesis, response to hypochlorite, cellular response to oxidative stress		-26.02742	repression	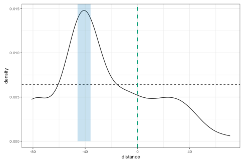
PA14_27730	fadE	acyl-CoA dehydrogenase	oxidoreductase activity, acting on the CH-CH group of donors, oxidation-reduction process, flavin adenine dinucleotide binding, acyl-CoA dehydrogenase activity, fatty acid beta-oxidation using acyl-CoA dehydrogenase	Fatty acid degradation, Fatty acid metabolism, Metabolic pathways	-25.89754	repression	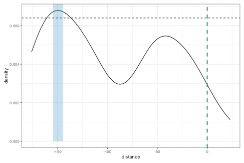
PA14_27755	NA	glutathione S-transferase	protein binding	Glutathione metabolism	-25.75142	repression	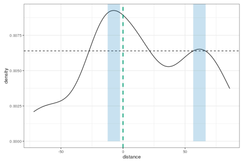
PA14_27810	NA	two-component response regulator	phosphorelay signal transduction system, DNA binding, regulation of transcription, DNA-templated	Two-component system	-25.83603	repression	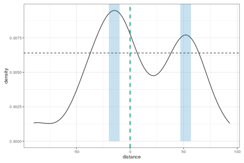
PA14_27830	NA	hypothetical protein	negative regulation of protein secretion, negative regulation of transcription, DNA-templated, stress response to copper ion		-25.96994	repression	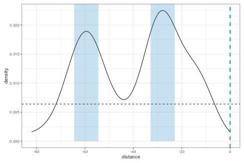
PA14_27910	NA	hypothetical protein			-26.02109	no impact	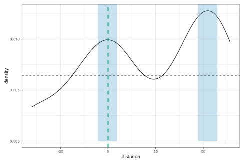
PA14_27990	NA	sialidase			-26.40647	no impact	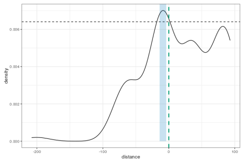
PA14_28010	NA	hypothetical protein			-26.54012	repression	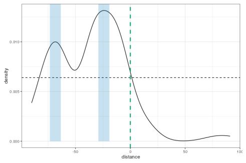
PA14_28070	NA	hypothetical protein	protein binding		-26.60333	repression	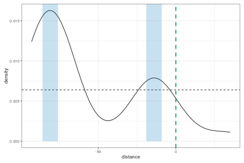
PA14_28130	NA	hypothetical protein	DNA binding		-26.71865	repression	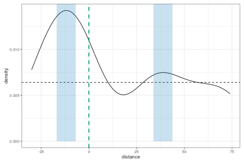

Modeled binding peaks for 1043 predicted targets of RsmA							
PA14 gene ID	gene name	description	GO terms	KEGG pathways	overall affinity score in RT	predicted effect on translation	binding site predictions
PA14_28170	NA	formate/nitrate transporter	membrane, transmembrane transporter activity, transmembrane transport		-26.27395	no impact	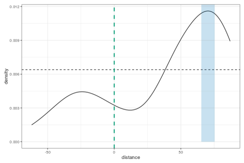
PA14_28290	NA	hypothetical protein	hydrolase activity		-26.04588	no impact	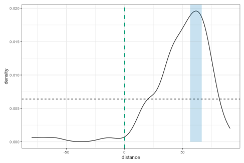
PA14_28400	NA	outer membrane OprD family porin	integral component of membrane		-26.52561	no impact	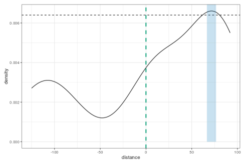
PA14_28410	NA	hypothetical protein			-26.33321	repression	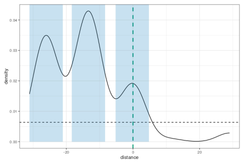
PA14_28440	NA	hypothetical protein			-26.66162	repression	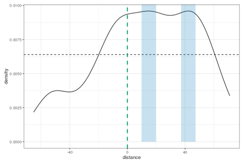
PA14_28450	eco	ecotin	serine-type endopeptidase inhibitor activity		-25.82337	repression	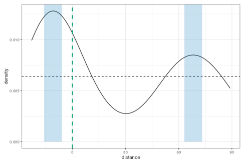
PA14_28530	NA	hypothetical protein			-26.32650	repression	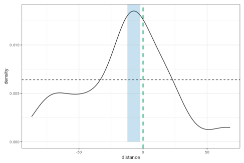
PA14_28600	NA	hypothetical protein			-25.98767	repression	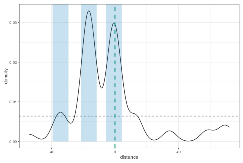
PA14_28630	NA	hydrolase			-26.31442	repression	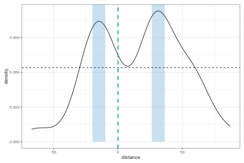
PA14_28650	thrS	threonyl-tRNA synthetase	aminoacyl-tRNA ligase activity, ATP binding, tRNA aminoacylation, nucleotide binding, tRNA aminoacylation for protein translation, threonine-tRNA ligase activity, cytoplasm, threonyl-tRNA aminoacylation	Aminoacyl-tRNA biosynthesis	-26.04016	repression	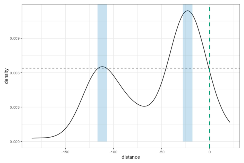

Modeled binding peaks for 1043 predicted targets of RsmA

PA14 gene ID	gene name	description	GO terms	KEGG pathways	overall affinity score in RT	predicted effect on translation	binding site predictions
PA14_28660	infC	translation initiation factor IF-3	translation initiation factor activity, translational initiation		-25.88104	repression	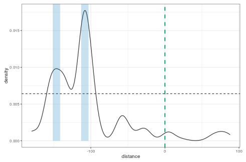
PA14_28670	rpmI	50S ribosomal protein L35	structural constituent of ribosome, ribosome, translation	Ribosome	-27.49188	no impact	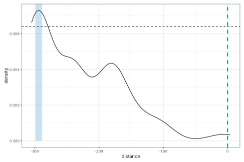
PA14_28690	pheS	phenylalanyl-tRNA synthetase subunit alpha	phenylalanine-tRNA ligase activity, nucleotide binding, tRNA binding, aminoacyl-tRNA ligase activity, ATP binding, tRNA aminoacylation, cytoplasm, phenylalanyl-tRNA aminoacylation	Aminoacyl-tRNA biosynthesis	-25.94277	repression	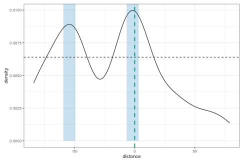
PA14_28720	ihfA	integration host factor subunit alpha	DNA binding, DNA recombination, regulation of transcription, DNA-templated		-26.96559	repression	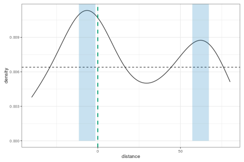
PA14_28910	NA	radical activating enzyme	catalytic activity, iron-sulfur cluster binding		-25.89496	repression	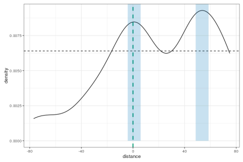
PA14_28940	NA	hypothetical protein			-26.66373	repression	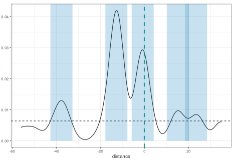
PA14_28950	NA	hypothetical protein	carbon-sulfur lyase activity		-26.88290	repression	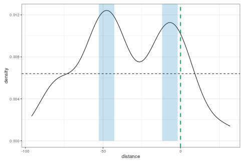
PA14_28990	NA	hypothetical protein			-25.92243	repression	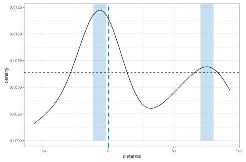
PA14_29020	cpo	chloroperoxidase			-26.83011	repression	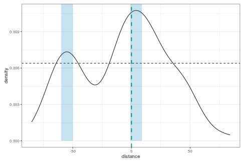
PA14_29060	NA	transcriptional regulator			-26.19856	repression	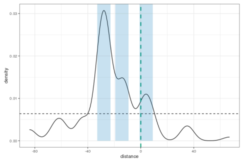

Modeled binding peaks for 1043 predicted targets of RsmA

PA14 gene ID	gene name	description	GO terms	KEGG pathways	overall affinity score in RT	predicted effect on translation	binding site predictions
PA14_29150	NA	hypothetical protein	carbon-sulfur lyase activity	formaldehyde oxidation II (glutathione-dependent), Methane metabolism	-25.79057	repression	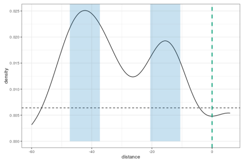
PA14_29190	NA	hypothetical protein			-25.91037	no impact	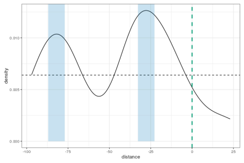
PA14_29260	NA	transcriptional regulator	DNA-binding transcription factor activity, regulation of transcription, DNA-templated, sequence-specific DNA binding, DNA binding		-27.72237	no impact	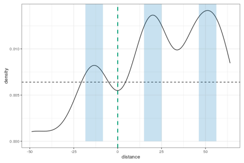
PA14_29270	NA	outer membrane lipoprotein			-26.14561	repression	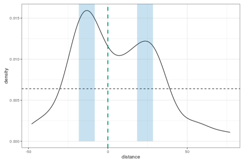
PA14_29360	pfeS	two-component sensor PfeS	signal transduction, integral component of membrane, phosphorelay sensor kinase activity, phosphorylation, transferase activity, transferring phosphorus-containing groups	Two-component system	-25.90853	repression	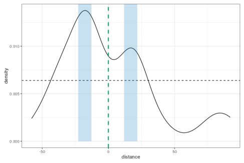
PA14_29400	NA	hypothetical protein			-26.49574	repression	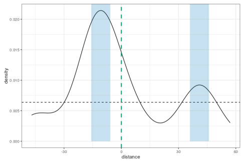
PA14_29520	NA	type II secretion system protein			-26.37774	repression	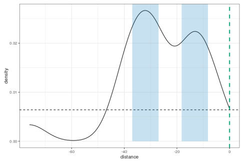
PA14_29530	NA	type II secretion system protein			-26.02191	repression	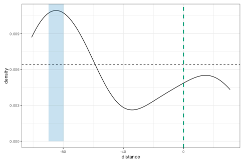
PA14_29560	NA	hypothetical protein			-26.02736	repression	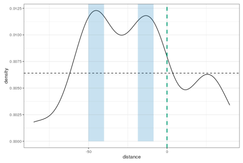
PA14_29570	NA	hypothetical protein			-26.53862	repression	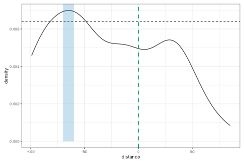

Modeled binding peaks for 1043 predicted targets of RsmA

PA14 gene ID	gene name	description	GO terms	KEGG pathways	overall affinity score in RT	predicted effect on translation	binding site predictions
PA14_29650	NA	hypothetical protein			-26.27926	repression	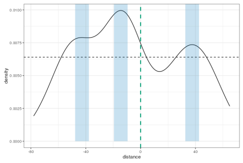
PA14_29720	NA	hypothetical protein			-25.98739	repression	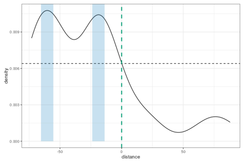
PA14_29730	bqsR	two-component response regulator BqsR	DNA binding, regulation of transcription, DNA-templated, phosphorelay signal transduction system, cellular response to iron(II) ion		-25.93389	repression	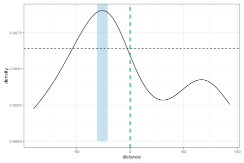
PA14_29750	NA	hypothetical protein			-26.74093	repression	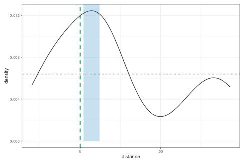
PA14_29890	nuoK	NADH dehydrogenase subunit K	oxidoreductase activity, acting on NAD(P)H, ATP synthesis coupled electron transport, oxidation-reduction process	Metabolic pathways, Oxidative phosphorylation	-26.05029	repression	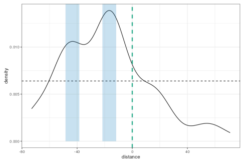
PA14_30020	nuoA	NADH dehydrogenase subunit A	oxidoreductase activity, acting on NAD(P)H, oxidation-reduction process, NADH dehydrogenase (ubiquinone) activity	Metabolic pathways, Oxidative phosphorylation	-25.78831	no impact	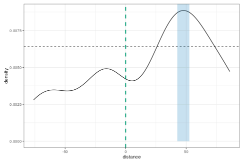
PA14_30190	icd	isocitrate dehydrogenase	isocitrate dehydrogenase (NADP+) activity, tricarboxylic acid cycle, oxidation-reduction process, oxidoreductase activity, acting on the CH-OH group of donors, NAD or NADP as acceptor, magnesium ion binding, NAD binding	2-Oxocarboxylic acid metabolism, Biosynthesis of amino acids, Biosynthesis of antibiotics, Biosynthesis of secondary metabolites, Carbon metabolism, Citrate cycle (TCA cycle), Glutathione metabolism, Metabolic pathways, Microbial metabolism in diverse environments	-25.94623	repression	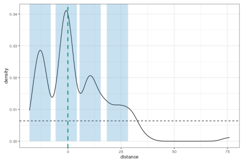
PA14_30200	cspD	cold-shock protein CspD	nucleic acid binding, cytoplasm, regulation of transcription, DNA-templated		-26.62991	no impact	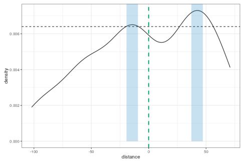
PA14_30210	clpS	ATP-dependent Clp protease adaptor protein ClpS	protein catabolic process, bacterial-type flagellum-dependent swarming motility, cellular response to antibiotic, single-species biofilm formation, single-species biofilm formation on inanimate substrate		-26.30038	no impact	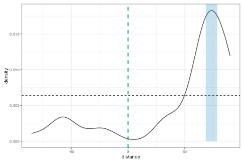
PA14_30230	clpA	ATP-dependent Clp protease, ATP-binding subunit ClpA	ATP binding, protein metabolic process, ATPase activity, protein unfolding		-25.89748	repression	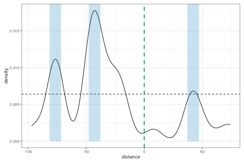

Modeled binding peaks for 1043 predicted targets of RsmA

PA14 gene ID	gene name	description	GO terms	KEGG pathways	overall affinity score in RT	predicted effect on translation	binding site predictions
PA14_30240	infA	translation initiation factor IF-1	translation initiation factor activity, translational initiation, RNA binding		-26.61809	repression	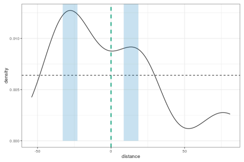
PA14_30340	cysG	siroheme synthase	methyltransferase activity, oxidation-reduction process, porphyrin-containing compound biosynthetic process, uroporphyrin-III C-methyltransferase activity, cobalamin biosynthetic process, siroheme biosynthetic process, precorrin-2 dehydrogenase activity, sirohydrochlorin ferrochelatase activity, NAD binding	Biosynthesis of secondary metabolites, cob(II)yrinate <i>a,c</i>-diamide biosynthesis I (early cobalt insertion), factor 430 biosynthesis, Metabolic pathways, Porphyrin and chlorophyll metabolism, Porphyrin and chlorophyll metabolism, siroheme biosynthesis	-25.81183	repression	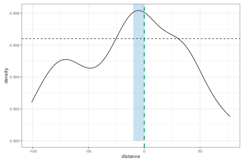
PA14_30350	NA	hypothetical protein	glutathione transferase activity, protein binding		-26.02175	repression	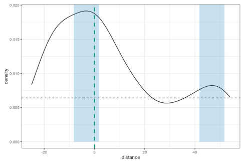
PA14_30370	NA	hypothetical protein			-25.82490	no impact	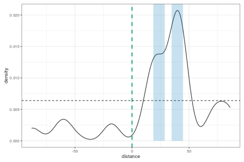
PA14_30560	NA	hypothetical protein			-26.43421	repression	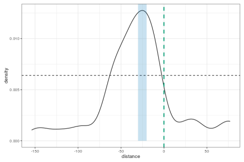
PA14_30630	pqsH	FAD-dependent monooxygenase	FAD binding	Biofilm formation - Pseudomonas aeruginosa, Quorum sensing	-27.04271	repression	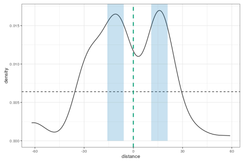
PA14_30750	NA	tryptophan oxygenase	tryptophan 2,3-dioxygenase activity, tryptophan catabolic process to kynurenine, heme binding, metal ion binding	Metabolic pathways, Tryptophan metabolism, Tryptophan metabolism	-26.43842	no impact	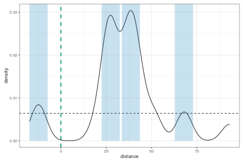
PA14_30790	NA	hypothetical protein	membrane, integral component of membrane		-26.53333	repression	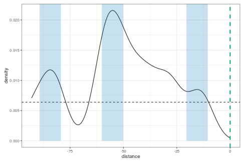
PA14_30820	NA	methyl-accepting chemotaxis transducer	signal transduction, integral component of membrane, membrane	Bacterial chemotaxis, Two-component system	-26.95724	repression	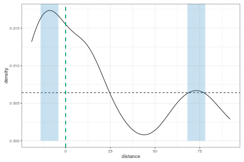
PA14_30840	NA	signal transduction histidine kinase	phosphorylation, transferase activity, transferring phosphorus-containing groups, phosphorelay sensor kinase activity, signal transduction		-26.60344	no impact	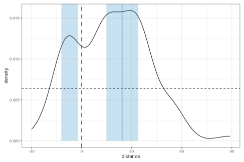

Modeled binding peaks for 1043 predicted targets of RsmA

PA14 gene ID	gene name	description	GO terms	KEGG pathways	overall affinity score in RT	predicted effect on translation	binding site predictions
PA14_31310	NA	hypothetical protein			-26.80463	repression	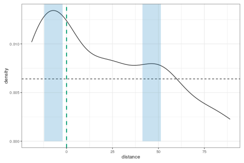
PA14_31360	NA	hypothetical protein			-27.07203	repression	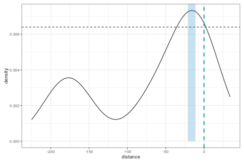
PA14_31390	NA	hypothetical protein			-25.85800	repression	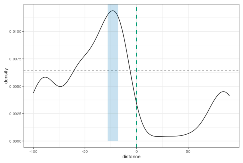
PA14_31420	NA	hypothetical protein			-26.07096	repression	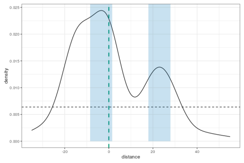
PA14_31440	NA	hypothetical protein			-26.69689	repression	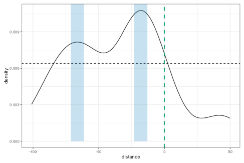
PA14_31460	NA	transporter	membrane		-26.52212	repression	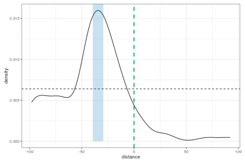
PA14_31580	NA	acyl-CoA dehydrogenase	oxidoreductase activity, acting on the CH-CH group of donors, oxidation-reduction process, flavin adenine dinucleotide binding	beta-Alanine metabolism, Biosynthesis of antibiotics, Biosynthesis of secondary metabolites, Carbon metabolism, Fatty acid degradation, Fatty acid metabolism, Metabolic pathways, Propanoate metabolism, Valine, leucine and isoleucine degradation	-27.33235	repression	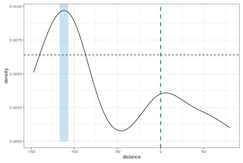
PA14_31720	NA	hypothetical protein			-26.93974	repression	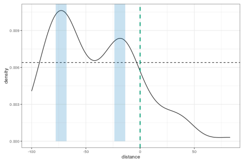
PA14_31760	NA	phosphatidate cytidylyltransferase			-26.37359	repression	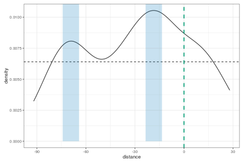
PA14_31800	NA	sodium:alanine symporter	sodium ion transport, alanine:sodium symporter activity, membrane, alanine transport		-26.38902	repression	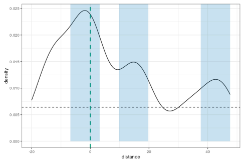

Modeled binding peaks for 1043 predicted targets of RsmA							
PA14 gene ID	gene name	description	GO terms	KEGG pathways	overall affinity score in RT	predicted effect on translation	binding site predictions
PA14_31810	tpx	thiol peroxidase	oxidoreductase activity, oxidoreductase activity, acting on peroxide as acceptor, oxidation-reduction process, cell redox homeostasis, thioredoxin peroxidase activity, antioxidant activity		-26.16785	repression	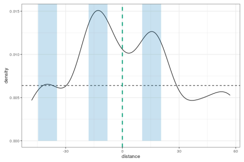
PA14_31870	NA	RND efflux membrane fusion protein	membrane, transmembrane transporter activity, transmembrane transport	Two-component system	-25.79007	repression	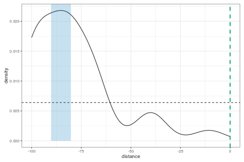
PA14_31970	czcC	CzcC family cobalt/zinc/cadmium efflux transporter outer membrane protein	efflux transmembrane transporter activity, transmembrane transport		-26.05385	repression	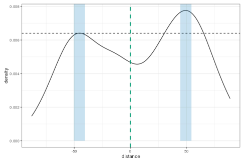
PA14_31990	czcB	cobalt/zinc/cadmium efflux RND transporter, membrane fusion protein, CzcB famil	membrane, transmembrane transporter activity, transmembrane transport		-26.04165	repression	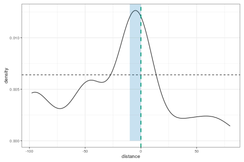
PA14_32100	xylY	toluate 1,2-dioxygenase subunit beta	cellular aromatic compound metabolic process, oxidation-reduction process	Benzoate degradation, Degradation of aromatic compounds, Fluorobenzoate degradation, Metabolic pathways, Microbial metabolism in diverse environments, Xylene degradation	-26.08987	repression	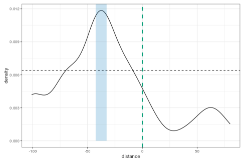
PA14_32110	xylZ	toluate 1,2-dioxygenase electron transfer component	oxidoreductase activity, oxidation-reduction process, electron transfer activity, iron-sulfur cluster binding, 2 iron, 2 sulfur cluster binding	Benzoate degradation, Degradation of aromatic compounds, Fluorobenzoate degradation, Metabolic pathways, Microbial metabolism in diverse environments, Xylene degradation	-25.79972	repression	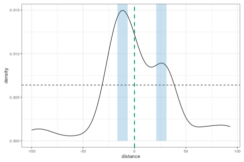
PA14_32230	catC	muconolactone delta-isomerase	cellular aromatic compound metabolic process	Benzoate degradation, Degradation of aromatic compounds, Metabolic pathways, Microbial metabolism in diverse environments	-25.78463	no impact	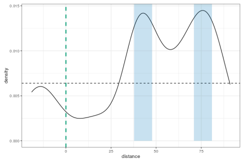
PA14_32270	NA	porin	integral component of membrane		-26.79213	repression	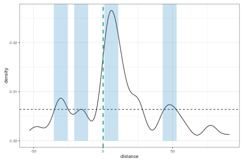
PA14_32300	NA	kinase	protein kinase activity, ATP binding, protein phosphorylation, protein binding		-26.05170	repression	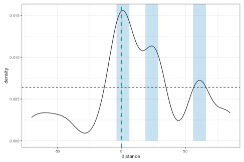
PA14_32470	NA	hypothetical protein			-26.18840	no impact	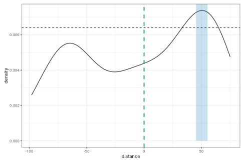

Modeled binding peaks for 1043 predicted targets of RsmA

PA14 gene ID	gene name	description	GO terms	KEGG pathways	overall affinity score in RT	predicted effect on translation	binding site predictions
PA14_32490	NA	hypothetical protein			-25.90426	repression	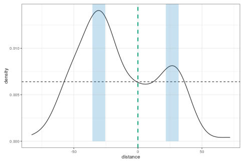
PA14_32630	NA	cytochrome P450	iron ion binding, oxidoreductase activity, acting on paired donors, with incorporation or reduction of molecular oxygen, heme binding, oxidation-reduction process		-25.80891	repression	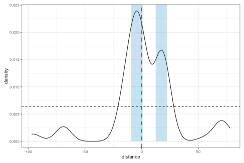
PA14_32700	NA	transcriptional regulator	DNA-binding transcription factor activity, regulation of transcription, DNA-templated		-25.95101	repression	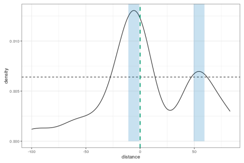
PA14_32770	NA	hypothetical protein			-27.02224	repression	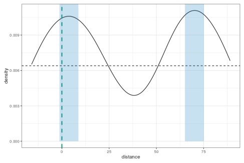
PA14_32780	NA	hypothetical protein	protein transport		-25.91193	repression	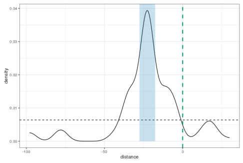
PA14_32790	NA	hypothetical protein			-25.88133	repression	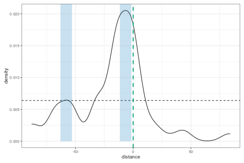
PA14_32880	NA	hypothetical protein			-26.32028	repression	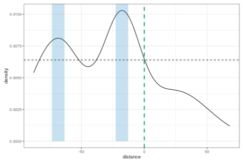
PA14_32905	NA	hypothetical protein	iron ion binding, cytoplasm, iron ion transport, enterochelin esterase activity		-27.10917	NA	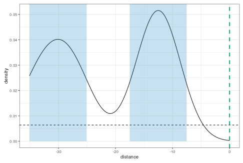
PA14_32970	NA	transcriptional regulator	DNA-binding transcription factor activity, regulation of transcription, DNA-templated		-25.97577	repression	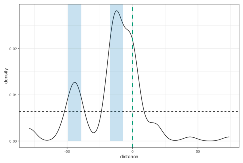
PA14_33040	gcvT2	glycine cleavage system protein T2	protein binding, aminomethyltransferase activity, glycine catabolic process	Biosynthesis of antibiotics, Biosynthesis of secondary metabolites, Carbon metabolism, Glycine, serine and threonine metabolism, Glycine, serine and threonine metabolism, Glycine, serine and threonine metabolism;Nitrogen metabolism;One carbon pool by folate, Glyoxylate and dicarboxylate metabolism, Metabolic pathways, One carbon pool by folate, One carbon pool by folate	-25.82749	repression	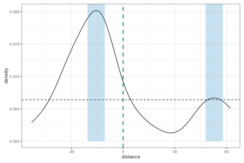

Modeled binding peaks for 1043 predicted targets of RsmA

PA14 gene ID	gene name	description	GO terms	KEGG pathways	overall affinity score in RT	predicted effect on translation	binding site predictions
PA14_33120	NA	hypothetical protein			-26.32505	repression	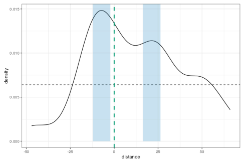
PA14_33170	NA	transcriptional regulator	DNA-binding transcription factor activity, regulation of transcription, DNA-templated		-25.90045	repression	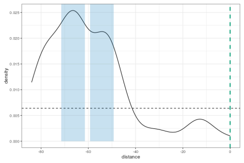
PA14_33190	NA	hypothetical protein	plasma membrane, transmembrane transporter activity, transmembrane transport		-26.25547	repression	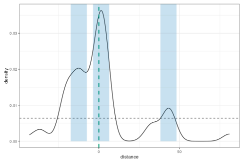
PA14_33280	pvdL	peptide synthase	phosphopantetheine binding, lipid biosynthetic process, catalytic activity	pyoverdine synthesis	-25.86004	repression	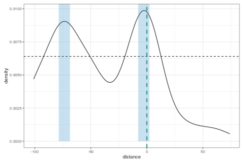
PA14_33410	NA	porin	integral component of membrane		-26.63941	repression	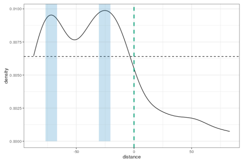
PA14_33420	NA	hydrolase	catalytic activity		-26.15758	repression	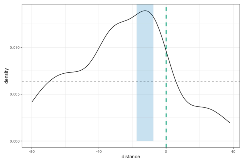
PA14_33520	NA	thioesterase	biosynthetic process, hydrolase activity, acting on ester bonds		-25.94658	repression	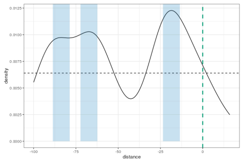
PA14_33690	pvdE	pyoverdine biosynthesis protein PvdE	ATP binding, integral component of membrane, ATPase-coupled transmembrane transporter activity, transmembrane transport, peptide transport, peptide transmembrane transporter activity, ATPase activity	ABC transporters	-25.81494	repression	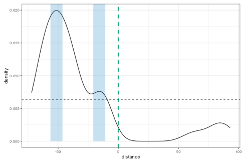
PA14_33740	pvdP	protein PvdP			-26.29546	repression	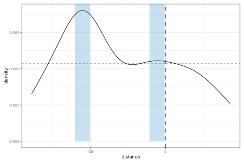
PA14_33760	NA	ABC transporter ATP-binding protein/permease	ATP binding, ATPase activity, efflux transmembrane transporter activity, xenobiotic detoxification by transmembrane export across the plasma membrane, membrane	ABC transporters	-25.93252	repression	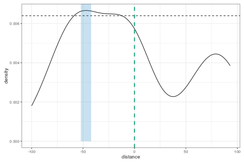

PA14 gene ID	gene name	description	GO terms	KEGG pathways	overall affinity score in RT	predicted effect on translation	binding site predictions
Modeled binding peaks for 1043 predicted targets of RsmA							
PA14_33800	NA	RNA polymerase sigma factor	DNA-binding transcription factor activity, DNA-templated transcription, initiation, regulation of transcription, DNA-templated, DNA binding, sigma factor activity		-25.93390	repression	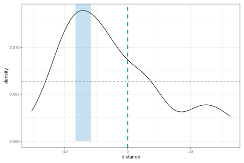
PA14_33810	pvdA	L-ornithine N5-oxygenase			-26.19276	repression	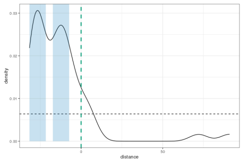
PA14_33820	pvdQ	penicillin acylase-related protein	hydrolase activity, acting on carbon-nitrogen (but not peptide) bonds, in linear amides, hydrolase activity, antibiotic biosynthetic process, N-acetyl-anhydromuramoyl-L-alanine amidase activity		-25.78850	repression	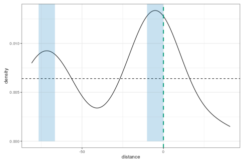
PA14_33830	NA	hypothetical protein			-26.88351	repression	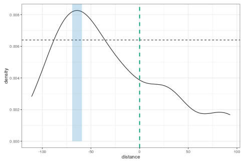
PA14_33880	NA	hypothetical protein			-26.72797	repression	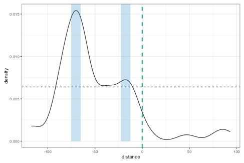
PA14_33920	NA	transcriptional regulator	phosphorelay signal transduction system, regulation of transcription, DNA-templated, DNA binding		-27.47880	repression	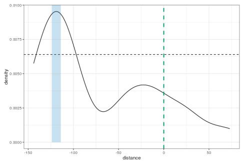
PA14_33940	tseF	TseF			-26.08340	repression	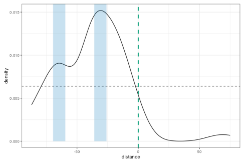
PA14_34020	hsiF3	HsiF3			-26.18784	repression	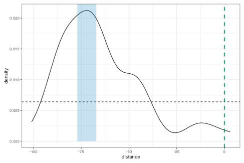
PA14_34070	hsiB3	HsiB3			-27.23878	repression	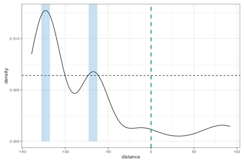
PA14_34100	hsiJ3	HsiJ3			-26.52365	no impact	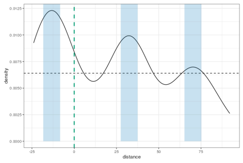

Modeled binding peaks for 1043 predicted targets of RsmA

PA14 gene ID	gene name	description	GO terms	KEGG pathways	overall affinity score in RT	predicted effect on translation	binding site predictions
PA14_34110	dotU3	DotU3			-26.65398	repression	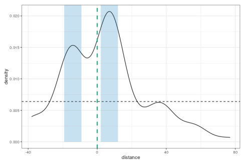
PA14_34250	NA	glycerophosphoryl diester phosphodiesterase	lipid metabolic process, phosphoric diester hydrolase activity	Glycerophospholipid metabolism	-26.65768	repression	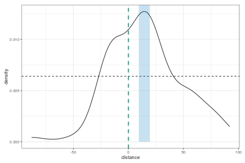
PA14_34540	NA	xenobiotic compound DszA family monooxygenase	monooxygenase activity, oxidoreductase activity, acting on paired donors, with incorporation or reduction of molecular oxygen, oxidation-reduction process		-26.58070	repression	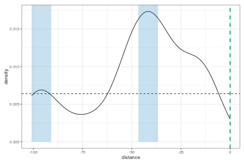
PA14_34720	NA	hypothetical protein			-26.23007	repression	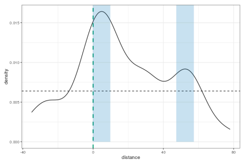
PA14_34740	NA	hypothetical protein			-26.13019	repression	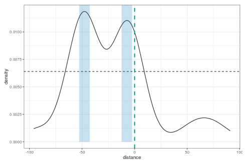
PA14_34810	NA	non-ribosomal peptide synthetase	catalytic activity		-25.85860	repression	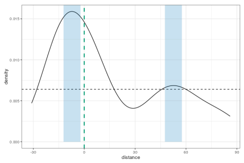
PA14_34820	NA	regulatory protein	oxidoreductase activity, oxidation-reduction process		-25.94038	repression	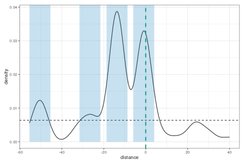
PA14_34900	NA	oxidoreductase	oxidoreductase activity, oxidation-reduction process		-26.09934	repression	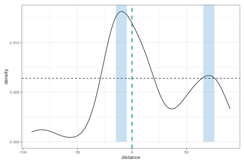
PA14_34940	NA	hypothetical protein			-26.24789	no impact	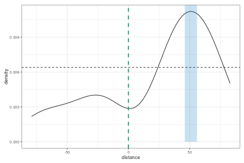
PA14_35060	NA	hypothetical protein			-26.46564	no impact	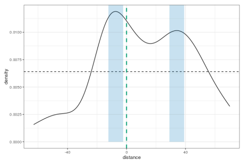

Modeled binding peaks for 1043 predicted targets of RsmA

PA14 gene ID	gene name	description	GO terms	KEGG pathways	overall affinity score in RT	predicted effect on translation	binding site predictions
PA14_35170	NA	redox-sensing activator of soxS	DNA binding, regulation of transcription, DNA-templated, response to oxidative stress, 2 iron, 2 sulfur cluster binding		-25.89750	no impact	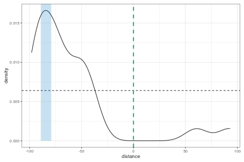
PA14_35200	NA	acetyltransferase	N-acetyltransferase activity		-26.35281	repression	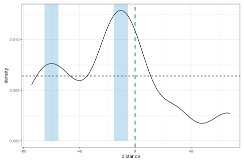
PA14_35290	gnd	gluconate dehydrogenase	oxidoreductase activity, acting on CH-OH group of donors, oxidation-reduction process, flavin adenine dinucleotide binding	Metabolic pathways, Microbial metabolism in diverse environments, Pentose phosphate pathway	-26.39052	no impact	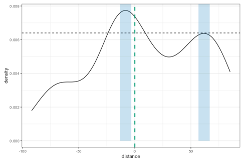
PA14_35500	bkdB	branched-chain alpha-keto acid dehydrogenase subunit E2	transferase activity, transferring acyl groups	Biosynthesis of antibiotics, Biosynthesis of secondary metabolites, Metabolic pathways, Propanoate metabolism, Valine, leucine and isoleucine degradation	-26.23396	repression	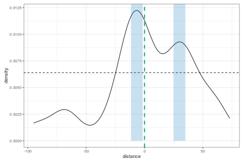
PA14_35590	pslM	FAD-binding dehydrogenase			-26.00246	repression	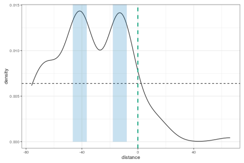
PA14_35640	pslI	transferase			-26.08339	NA	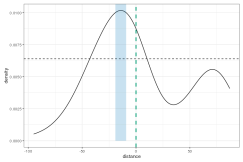
PA14_35670	pslG	glycosyl hydrolase	hydrolase activity, hydrolyzing O-glycosyl compounds, carbohydrate metabolic process	Biofilm formation - Pseudomonas aeruginosa	-26.27623	repression	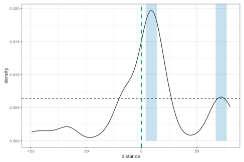
PA14_36060	NA	hypothetical protein	catalytic activity		-26.28597	repression	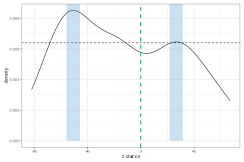
PA14_36120	NA	MFS transporter	integral component of plasma membrane, transmembrane transport		-25.82691	repression	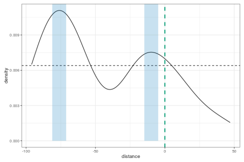
PA14_36230	NA	amino acid ABC transporter permease	membrane, transmembrane transport, integral component of membrane, transmembrane transporter activity, ATP-binding cassette (ABC) transporter complex, nitrogen compound transport		-26.12596	repression	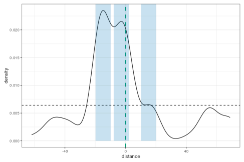

PA14 gene ID	gene name	description	GO terms	KEGG pathways	overall affinity score in RT	predicted effect on translation	binding site predictions
Modeled binding peaks for 1043 predicted targets of RsmA							
PA14_36250	NA	hypothetical protein			-25.95537	no impact	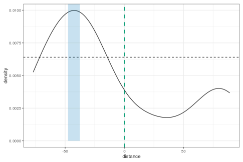
PA14_36270	NA	dehydrogenase	oxidoreductase activity, oxidation-reduction process, NADP binding, NAD binding		-26.51357	repression	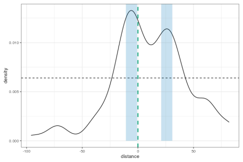
PA14_36350	NA	hypothetical protein			-26.13276	repression	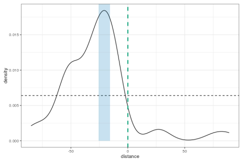
PA14_36360	NA	hypothetical protein	transmembrane transport		-26.50085	repression	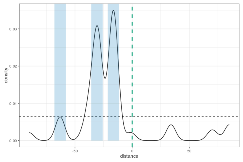
PA14_36420	NA	sensor/response regulator hybrid	phosphorylation, transferase activity, transferring phosphorus-containing groups, phosphorelay sensor kinase activity, signal transduction, phosphorelay signal transduction system		-26.82739	repression	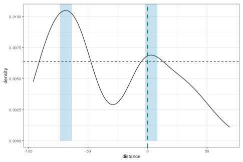
PA14_36500	NA	hypothetical protein			-25.94253	repression	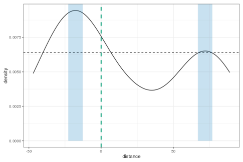
PA14_36530	NA	hypothetical protein			-25.77286	repression	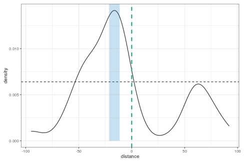
PA14_36550	NA	hypothetical protein			-25.91810	no impact	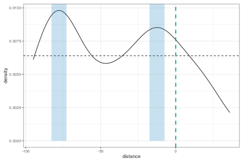
PA14_36570	glgA	glycogen synthase	glycogen (starch) synthase activity	Biosynthesis of secondary metabolites, Metabolic pathways, Starch and sucrose metabolism	-26.89338	repression	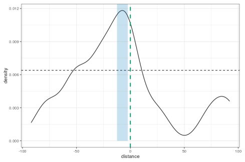
PA14_36670	NA	hypothetical protein			-26.57906	no impact	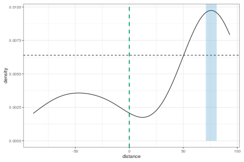

Modeled binding peaks for 1043 predicted targets of RsmA							
PA14 gene ID	gene name	description	GO terms	KEGG pathways	overall affinity score in RT	predicted effect on translation	binding site predictions
PA14_36810	katE	hydroperoxidase II	catalase activity, response to oxidative stress, heme binding, oxidation-reduction process	Biosynthesis of antibiotics, Biosynthesis of secondary metabolites, Carbon metabolism, Glyoxylate and dicarboxylate metabolism, Glyoxylate and dicarboxylate metabolism, Tryptophan metabolism, Tryptophan metabolism	-26.88837	repression	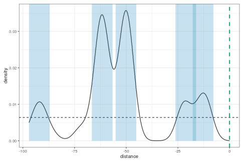
PA14_36820	NA	hypothetical protein			-25.98320	no impact	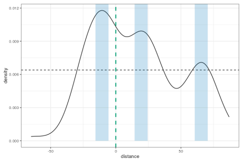
PA14_36850	NA	hypothetical protein			-26.54302	repression	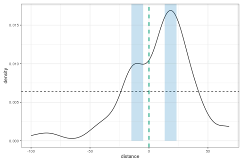
PA14_37080	NA	hypothetical protein	transcription regulatory region DNA binding		-26.54359	repression	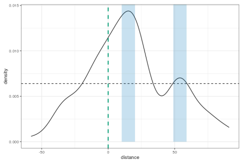
PA14_37090	NA	aldehyde dehydrogenase	oxidoreductase activity, oxidation-reduction process, oxidoreductase activity, acting on the aldehyde or oxo group of donors, NAD or NADP as acceptor		-25.89539	repression	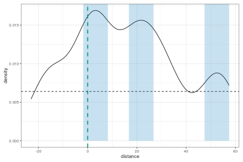
PA14_37120	NA	LysR family transcriptional regulator	DNA-binding transcription factor activity, regulation of transcription, DNA-templated		-26.33448	repression	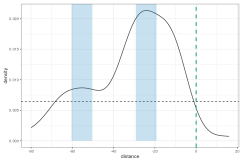
PA14_37140	NA	LysR family transcriptional regulator	DNA-binding transcription factor activity, regulation of transcription, DNA-templated		-26.19753	repression	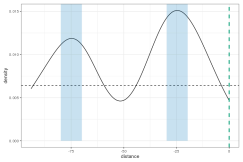
PA14_37200	NA	hypothetical protein			-26.44452	repression	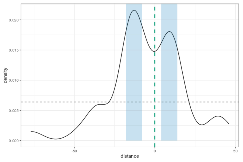
PA14_37350	NA	hypothetical protein			-26.66673	repression	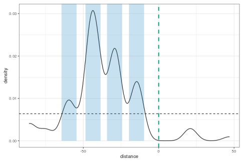
PA14_37410	NA	hypothetical protein			-26.11195	repression	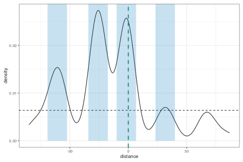

Modeled binding peaks for 1043 predicted targets of RsmA

PA14 gene ID	gene name	description	GO terms	KEGG pathways	overall affinity score in RT	predicted effect on translation	binding site predictions
PA14_37420	NA	transmembrane sensor protein			-26.46435	no impact	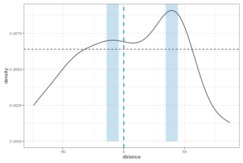
PA14_37440	NA	MFS transporter	integral component of plasma membrane, transmembrane transport		-27.04293	repression	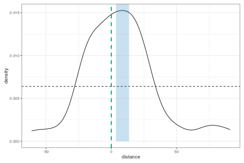
PA14_37510	NA	hypothetical protein			-26.73276	repression	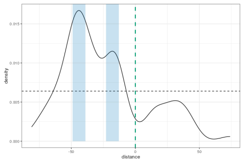
PA14_37560	NA	asparagine synthetase, glutamine-hydrolysing	asparagine synthase (glutamine-hydrolyzing) activity, asparagine biosynthetic process	Alanine, aspartate and glutamate metabolism, Alanine, aspartate and glutamate metabolism, Biosynthesis of secondary metabolites, Metabolic pathways	-26.58029	repression	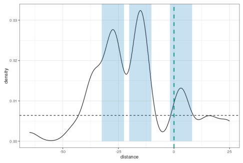
PA14_37580	NA	leucine-responsive regulatory protein	DNA-binding transcription factor activity, regulation of transcription, DNA-templated, sequence-specific DNA binding		-25.82896	no impact	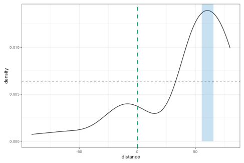
PA14_37730	NA	TonB dependent receptor			-26.50438	repression	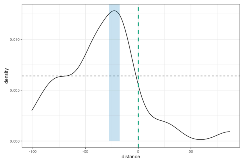
PA14_37810	pcoB	copper resistance protein B	copper ion binding, cellular copper ion homeostasis, cell outer membrane		-26.34793	repression	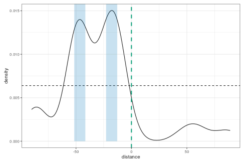
PA14_37830	NA	pyridoxal-phosphate dependent protein	catalytic activity	Metabolic pathways, Sulfur relay system, Thiamine metabolism, Thiamine metabolism, [2Fe-2S] iron-sulfur cluster biosynthesis	-26.09970	repression	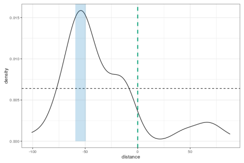
PA14_37840	sppD	ABC transporter ATP-binding protein, SppD	ATP binding, ATPase activity, nucleotide binding, peptide transport	ABC transporters	-25.76776	no impact	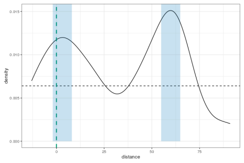
PA14_38110	NA	serine/threonine transporter SstT	symporter activity, integral component of membrane, threonine transport, serine transport		-26.54428	repression	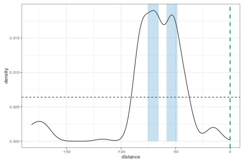

Modeled binding peaks for 1043 predicted targets of RsmA

PA14 gene ID	gene name	description	GO terms	KEGG pathways	overall affinity score in RT	predicted effect on translation	binding site predictions
PA14_38190	NA	hypothetical protein	methyltransferase activity		-26.61305	repression	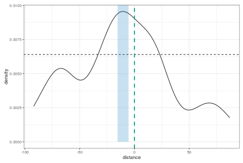
PA14_38200	NA	thiamine pyrophosphate protein	catalytic activity, thiamine pyrophosphate binding, magnesium ion binding	2-Oxocarboxylic acid metabolism, Biosynthesis of amino acids, Biosynthesis of antibiotics, Biosynthesis of secondary metabolites, Butanoate metabolism, C5-Branched dibasic acid metabolism, Metabolic pathways, Pantothenate and CoA biosynthesis, Valine, leucine and isoleucine biosynthesis	-26.91223	repression	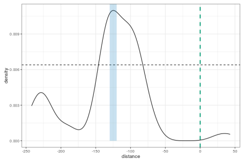
PA14_38210	NA	hypothetical protein			-26.41769	repression	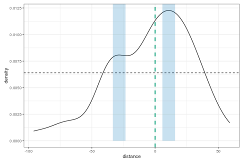
PA14_38270	NA	hypothetical protein			-25.87066	no impact	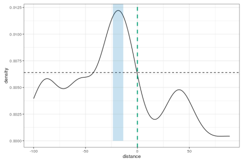
PA14_38360	NA	nucleotide sugar dehydrogenase	oxidoreductase activity, acting on the CH-OH group of donors, NAD or NADP as acceptor, NAD binding, oxidation-reduction process, polysaccharide biosynthetic process, UDP-glucose 6-dehydrogenase activity	Amino sugar and nucleotide sugar metabolism, Ascorbate and aldarate metabolism, Metabolic pathways, Pentose and glucuronate interconversions	-25.77618	no impact	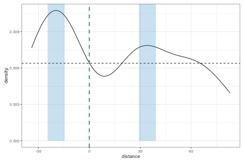
PA14_38395	NA	periplasmic multidrug efflux lipoprotein	membrane, transmembrane transporter activity, transmembrane transport	beta-Lactam resistance, Two-component system	-26.12439	repression	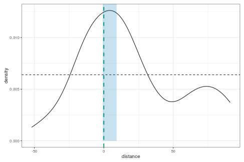
PA14_38490	gnyL	hydroxymethylglutaryl-CoA lyase	hydroxymethylglutaryl-CoA lyase activity, catalytic activity	Butanoate metabolism, Geraniol degradation, Metabolic pathways, Synthesis and degradation of ketone bodies, Valine, leucine and isoleucine degradation	-26.06754	no impact	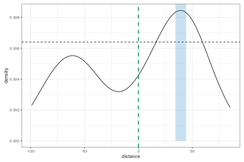
PA14_38560	NA	MFS transporter	integral component of plasma membrane, transmembrane transport, integral component of membrane, transmembrane transporter activity		-26.45491	repression	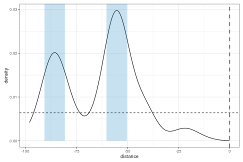
PA14_38610	NA	hypothetical protein			-25.79016	repression	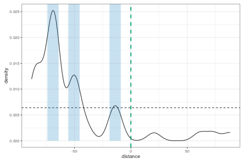
PA14_38690	NA	acetoacetyl-CoA synthetase	catalytic activity, lipid metabolic process, acetoacetate-CoA ligase activity	Butanoate metabolism, Valine, leucine and isoleucine degradation	-26.53530	repression	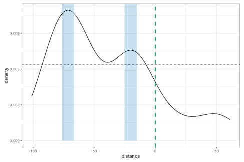

Modeled binding peaks for 1043 predicted targets of RsmA

PA14 gene ID	gene name	description	GO terms	KEGG pathways	overall affinity score in RT	predicted effect on translation	binding site predictions
PA14_38800	pqqC	pyrroloquinoline quinone biosynthesis protein PqqC	pyrroloquinoline quinone biosynthetic process, oxidation-reduction process		-26.95991	no impact	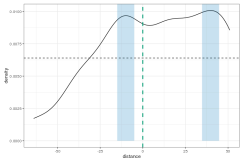
PA14_38825	pqqA	coenzyme PQQ synthesis protein PqqA	pyrroloquinoline quinone biosynthetic process	Pyrroloquinoline quinone biosynthesis	-26.49150	no impact	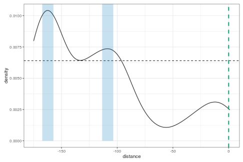
PA14_38900	NA	two-component response regulator	phosphorelay signal transduction system, regulation of transcription, DNA-templated, DNA binding		-26.12861	no impact	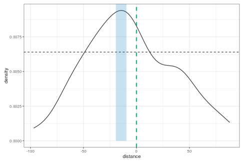
PA14_38990	NA	hypothetical protein			-27.85489	no impact	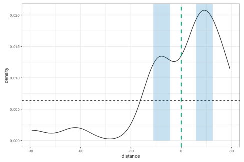
PA14_39190	bacA	UDP pyrophosphate phosphatase	membrane, dephosphorylation, undecaprenyl-diphosphatase activity	Peptidoglycan biosynthesis	-27.46641	repression	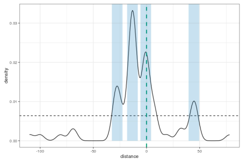
PA14_39260	NA	hypothetical protein			-26.13349	repression	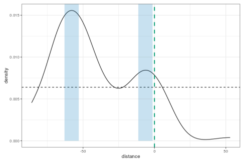
PA14_39280	rbsK	ribokinase	ribokinase activity, D-ribose metabolic process, phosphotransferase activity, alcohol group as acceptor, kinase activity	Pentose phosphate pathway	-26.70863	no impact	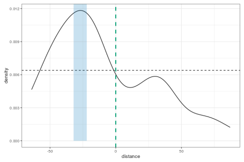
PA14_39300	rbsR	ribose operon repressor RbsR	DNA binding, regulation of transcription, DNA-templated		-26.26325	repression	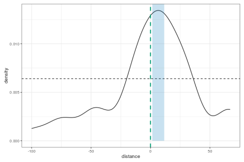
PA14_39500	NA	hypothetical protein			-26.09176	repression	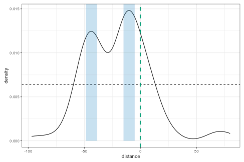
PA14_39520	NA	hydroxylase large subunit	oxidoreductase activity, oxidation-reduction process	Metabolic pathways, Microbial metabolism in diverse environments, Purine metabolism	-26.07114	no impact	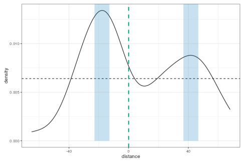

Modeled binding peaks for 1043 predicted targets of RsmA

PA14 gene ID	gene name	description	GO terms	KEGG pathways	overall affinity score in RT	predicted effect on translation	binding site predictions
PA14_39560	NA	chemotaxis transducer	signal transduction, membrane, transmembrane signaling receptor activity, chemotaxis	Bacterial chemotaxis, Two-component system	-26.48256	repression	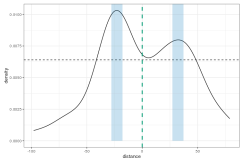
PA14_39590	metE	5-methyltetrahydropteroyltriglutamate/homocysteine S-methyltransferase	5-methyltetrahydropteroyltriglutamate-homocysteine S-methyltransferase activity, zinc ion binding, methionine biosynthetic process, cellular amino acid biosynthetic process	Biosynthesis of amino acids, Biosynthesis of secondary metabolites, Cysteine and methionine metabolism, Metabolic pathways, Selenocompound metabolism	-25.75036	no impact	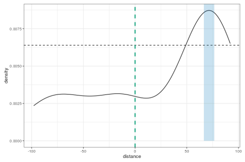
PA14_39750	NA	amino acid permease	amino acid transmembrane transport, integral component of plasma membrane, aromatic amino acid transmembrane transporter activity, aromatic amino acid transport		-25.79305	repression	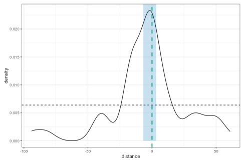
PA14_39890	phzF2	phenazine biosynthesis protein	catalytic activity, biosynthetic process	Phenazine biosynthesis, Quorum sensing	-26.44280	NA	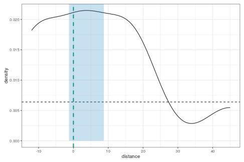
PA14_39945	phzC2	phenazine biosynthesis protein PhzC	catalytic activity, 3-deoxy-7-phosphoheptulonate synthase activity, aromatic amino acid family biosynthetic process	Biosynthesis of amino acids, Biosynthesis of antibiotics, Biosynthesis of secondary metabolites, Metabolic pathways, Phenazine biosynthesis, Phenylalanine, tyrosine and tryptophan biosynthesis, Quorum sensing	-28.02415	repression	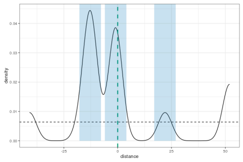
PA14_39970	phzA2	phenazine biosynthesis protein	antibiotic biosynthetic process, phenazine biosynthetic process	Phenazine biosynthesis, Quorum sensing	-25.97961	repression	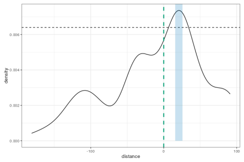
PA14_39980	qscR	transcriptional regulator	regulation of transcription, DNA-templated, DNA binding	Phenazine biosynthesis	-27.13635	no impact	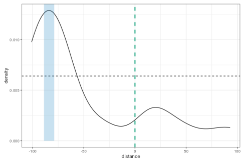
PA14_40020	NA	hypothetical protein	lipid metabolic process		-25.90425	no impact	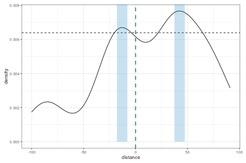
PA14_40100	NA	hypothetical protein			-25.78868	repression	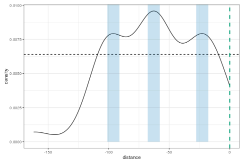
PA14_40120	polB	DNA polymerase II	nucleotide binding, nucleic acid binding, DNA-directed DNA polymerase activity, DNA binding		-26.07252	repression	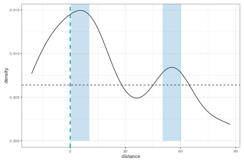

PA14 gene ID	gene name	description	GO terms	KEGG pathways	overall affinity score in RT	predicted effect on translation	binding site predictions
Modeled binding peaks for 1043 predicted targets of RsmA							
PA14_40230	NA	secretion protein			-26.89720	repression	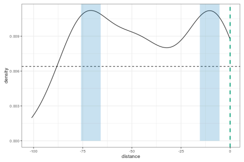
PA14_40250	NA	outer membrane protein	efflux transmembrane transporter activity, transmembrane transport		-27.39896	no impact	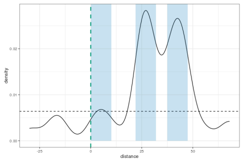
PA14_40300	NA	hypothetical protein			-26.64647	repression	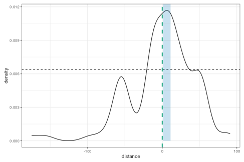
PA14_40310	NA	acyl carrier protein	phosphopantetheine binding, fatty acid biosynthetic process		-26.39792	repression	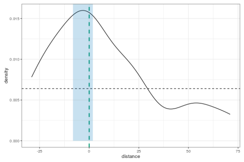
PA14_40490	NA	hypothetical protein			-26.34378	repression	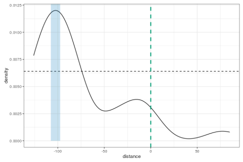
PA14_40510	ccoN-2	cbb3-type cytochrome c oxidase subunit I	oxidation-reduction process, cytochrome-c oxidase activity, aerobic respiration, integral component of membrane, heme binding, plasma membrane respiratory chain complex IV	aerobic respiration I (cytochrome c), aerobic respiration II (cytochrome c) (yeast), arsenite oxidation I (respiratory), Fe(II) oxidation, Metabolic pathways, Oxidative phosphorylation, Oxidative phosphorylation, Respiratory chain; terminal step, Two-component system	-25.80670	repression	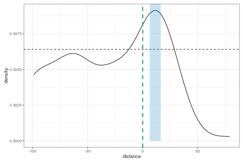
PA14_40660	NA	hypothetical protein			-25.88398	repression	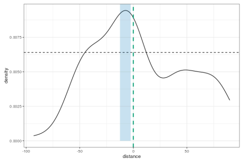
PA14_40780	NA	hypothetical protein			-26.00768	repression	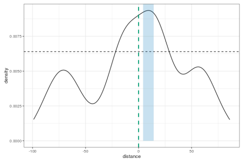
PA14_40790	NA	transcriptional regulator	DNA binding		-25.86526	repression	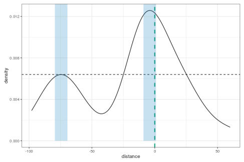
PA14_40830	NA	oxidoreductase	oxidation-reduction process, oxidoreductase activity		-25.84296	no impact	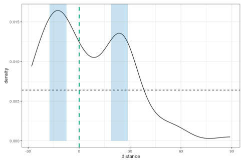

Modeled binding peaks for 1043 predicted targets of RsmA

PA14 gene ID	gene name	description	GO terms	KEGG pathways	overall affinity score in RT	predicted effect on translation	binding site predictions
PA14_40840	NA	periplasmic protease	proteolysis, peptidase activity, serine-type endopeptidase activity, plasma membrane		-26.35110	repression	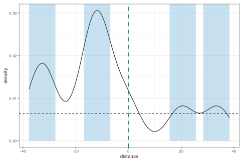
PA14_40910	NA	LysR family transcriptional regulatory protein	DNA-binding transcription factor activity, regulation of transcription, DNA-templated		-26.16122	repression	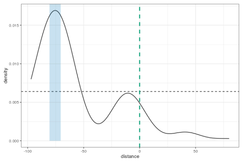
PA14_40930	NA	hypothetical protein			-25.93181	repression	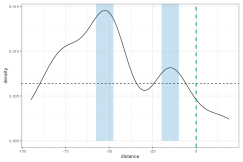
PA14_40960	NA	pilin biosynthetic protein	phosphorelay signal transduction system		-25.87612	no impact	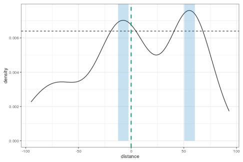
PA14_41010	NA	amino acid permease	membrane, transmembrane transporter activity, transmembrane transport		-26.02811	repression	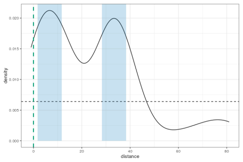
PA14_41060	rnhA	ribonuclease H	nucleic acid binding, RNA-DNA hybrid ribonuclease activity	DNA replication	-26.31384	no impact	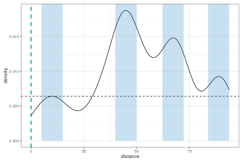
PA14_41170	fabI	NADH-dependent enoyl-ACP reductase	enoyl-[acyl-carrier-protein] reductase (NADH) activity, fatty acid biosynthetic process, oxidation-reduction process, enoyl-[acyl-carrier-protein] reductase activity	(5Z)-dodecenoate biosynthesis II, 8-amino-7-oxononanoate biosynthesis I, <i>cis</i>-vaccenate biosynthesis, Biotin metabolism, Fatty acid biosynthesis, Fatty acid biosynthesis, Fatty acid metabolism, gondoate biosynthesis (anaerobic), Metabolic pathways, mycolate biosynthesis, oleate biosynthesis IV (anaerobic), palmitate biosynthesis II (bacteria and plants), palmitoleate biosynthesis I (from (5Z)-dodec-5-enoate), stearate biosynthesis II (bacteria and plants), superpathway of mycolate biosynthesis	-26.94666	repression	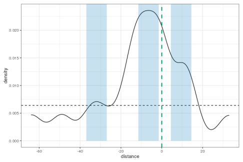
PA14_41190	ppiD	peptidyl-prolyl cis-trans isomerase D	peptidyl-prolyl cis-trans isomerase activity		-26.08300	no impact	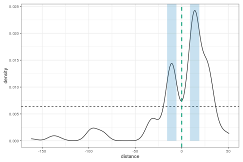
PA14_41480	nasS	hypothetical protein			-25.88176	repression	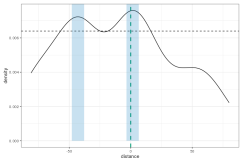
PA14_41530	nirB	assimilatory nitrite reductase large subunit	oxidoreductase activity, heme binding, iron-sulfur cluster binding, oxidation-reduction process, nitrite reductase [NAD(P)H] activity, nitrate assimilation, flavin adenine dinucleotide binding, NADP binding	assimilatory sulfate reduction III, Microbial metabolism in diverse environments, Nitrogen metabolism, Sulfur metabolism	-25.85741	repression	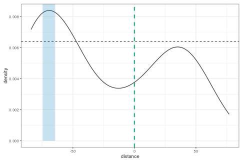

Modeled binding peaks for 1043 predicted targets of RsmA

PA14 gene ID	gene name	description	GO terms	KEGG pathways	overall affinity score in RT	predicted effect on translation	binding site predictions
PA14_41540	nirD	assimilatory nitrite reductase small subunit	nitrite reductase [NAD(P)H] activity, oxidation-reduction process, oxidoreductase activity, 2 iron, 2 sulfur cluster binding	Microbial metabolism in diverse environments, Nitrogen metabolism	-25.96966	no impact	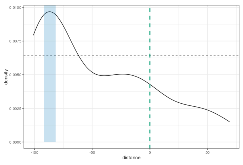
PA14_41560	NA	assimilatory nitrate reductase	oxidoreductase activity, oxidation-reduction process, molybdopterin cofactor binding, 4 iron, 4 sulfur cluster binding, electron transfer activity	Microbial metabolism in diverse environments, nitrate reduction IV (dissimilatory), Nitrogen metabolism, Nitrogen metabolism	-26.12420	repression	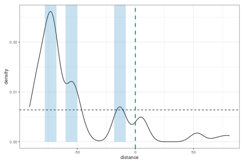
PA14_41570	oprF	major porin and structural outer membrane porin OprF precursor	cell outer membrane, integral component of membrane, calcium ion binding, porin activity, outer membrane, adhesion of symbiont to host		-26.10800	repression	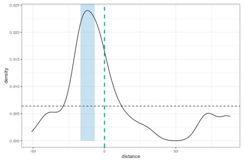
PA14_41575	sigX	RNA polymerase sigma factor SigX	DNA-binding transcription factor activity, DNA-templated transcription, initiation, regulation of transcription, DNA-templated, sigma factor activity, positive regulation of cell growth		-26.54150	repression	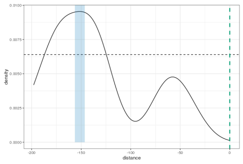
PA14_41730	NA	hypothetical protein	ATP binding, metal ion binding		-26.37460	no impact	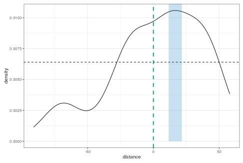
PA14_41750	NA	hypothetical protein			-26.06864	no impact	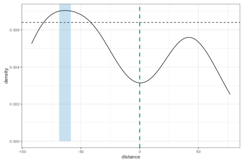
PA14_41780	NA	hypothetical protein	ATP binding, proteolysis, peptidase activity, integral component of membrane		-26.27066	repression	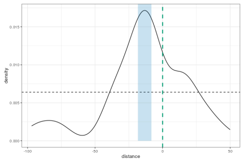
PA14_41790	NA	hypothetical protein			-26.30129	repression	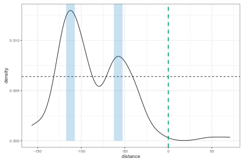
PA14_41860	NA	hypothetical protein			-25.86570	no impact	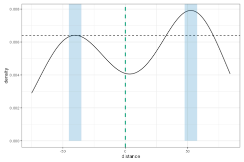
PA14_41880	NA	universal stress protein			-27.67748	repression	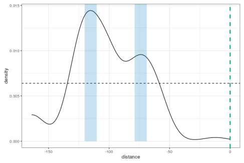

Modeled binding peaks for 1043 predicted targets of RsmA

PA14 gene ID	gene name	description	GO terms	KEGG pathways	overall affinity score in RT	predicted effect on translation	binding site predictions
PA14_41900	panE	2-dehydropantoate 2-reductase	oxidation-reduction process, oxidoreductase activity, 2-dehydropantoate 2-reductase activity, pantothenate biosynthetic process	Biosynthesis of secondary metabolites, Metabolic pathways, Pantothenate and CoA biosynthesis, Pantothenate and CoA biosynthesis, phosphopantothenate biosynthesis III (archaebacteria)	-27.02706	repression	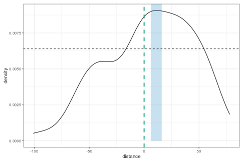
PA14_41910	NA	hypothetical protein			-25.95312	repression	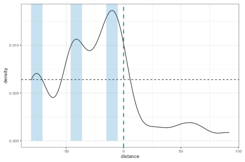
PA14_41930	NA	hypothetical protein			-26.25638	no impact	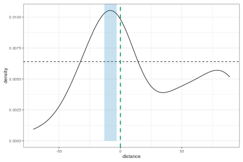
PA14_42020	NA	hypothetical protein	protein binding, electron transfer activity, protein disulfide oxidoreductase activity, cell redox homeostasis		-27.02148	repression	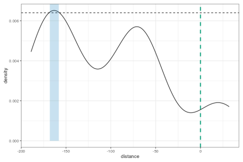
PA14_42030	NA	hypothetical protein			-25.96684	repression	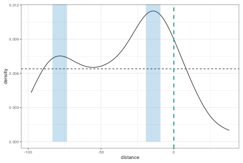
PA14_42080	NA	3-hydroxyacyl-CoA dehydrogenase	3-hydroxyacyl-CoA dehydrogenase activity, fatty acid metabolic process, oxidoreductase activity, oxidation-reduction process, catalytic activity	Benzoate degradation, beta-Alanine metabolism, Biosynthesis of antibiotics, Biosynthesis of secondary metabolites, Biosynthesis of unsaturated fatty acids, Butanoate metabolism, Caprolactam degradation, Carbon metabolism, Fatty acid degradation, Fatty acid metabolism, Geraniol degradation, Limonene and pinene degradation, Lysine degradation, Metabolic pathways, Microbial metabolism in diverse environments, Propanoate metabolism, Tryptophan metabolism, Valine, leucine and isoleucine degradation	-26.63659	repression	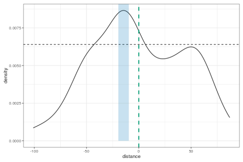
PA14_42090	NA	acetyl-CoA acetyltransferase	transferase activity, transferring acyl groups other than amino-acyl groups, catalytic activity	Benzoate degradation, Biosynthesis of antibiotics, Biosynthesis of secondary metabolites, Butanoate metabolism, Carbon metabolism, Fatty acid degradation, Fatty acid metabolism, Glyoxylate and dicarboxylate metabolism, Lysine degradation, Metabolic pathways, Microbial metabolism in diverse environments, Propanoate metabolism, Pyruvate metabolism, Synthesis and degradation of ketone bodies, Terpenoid backbone biosynthesis, Tryptophan metabolism, Two-component system, Valine, leucine and isoleucine degradation	-25.91760	no impact	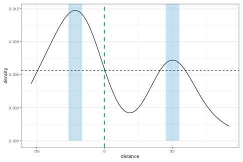
PA14_42100	NA	hypothetical protein	integral component of membrane, transmembrane transport		-27.17437	repression	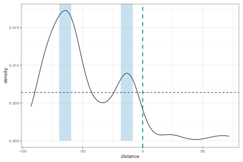
PA14_42130	NA	hypothetical protein	threonine-type endopeptidase activity, proteasome core complex, proteolysis involved in cellular protein catabolic process		-27.53686	repression	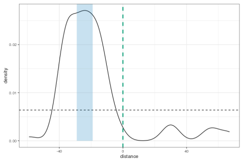
PA14_42150	NA	hypothetical protein			-26.21547	repression	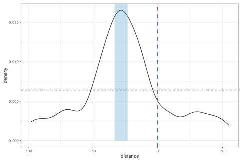

Modeled binding peaks for 1043 predicted targets of RsmA

PA14 gene ID	gene name	description	GO terms	KEGG pathways	overall affinity score in RT	predicted effect on translation	binding site predictions
PA14_42200	NA	hypothetical protein			-25.86085	repression	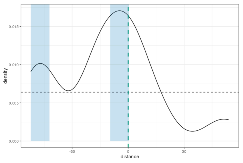
PA14_42280	pscI	type III export protein PscI	protein secretion, protein secretion by the type III secretion system		-26.06406	repression	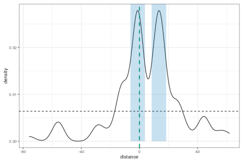
PA14_42310	pscF	type III export protein PscF	type III protein secretion system complex, pathogenesis, protein transport, protein secretion by the type III secretion system	Bacterial secretion system	-25.92634	no impact	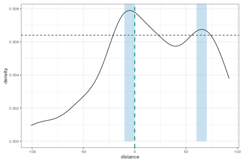
PA14_42340	pscD	type III export protein PscD	protein secretion by the type III secretion system		-26.56853	repression	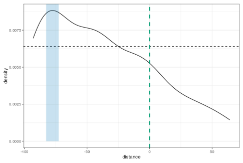
PA14_42360	pscB	type III export apparatus protein	cytoplasm, regulation of protein secretion, protein secretion by the type III secretion system		-26.67391	no impact	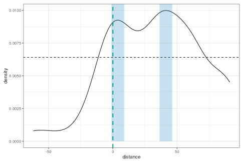
PA14_42380	NA	hypothetical protein	negative regulation of protein secretion, negative regulation of transcription, DNA-templated, negative regulation of DNA binding, negative regulation of protein binding, cellular response to calcium ion		-25.94342	repression	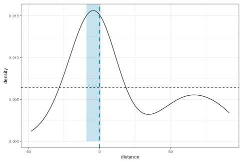
PA14_42390	exsA	transcriptional regulator ExsA	DNA binding, DNA-binding transcription factor activity, regulation of transcription, DNA-templated, sequence-specific DNA binding	Biofilm formation - Pseudomonas aeruginosa	-26.71337	repression	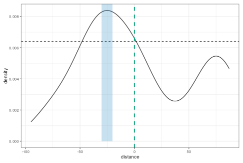
PA14_42450	popB	translocator protein PopB	pathogenesis, chaperone binding, translocation of peptides or proteins into host		-26.42863	no impact	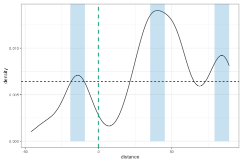
PA14_42530	NA	type III secretion protein	protein secretion		-25.84286	repression	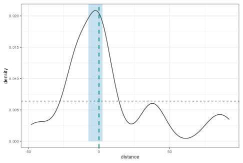
PA14_42540	NA	protein in type III secretion	negative regulation of protein secretion		-26.40519	repression	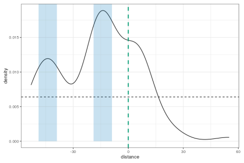

Modeled binding peaks for 1043 predicted targets of RsmA

PA14 gene ID	gene name	description	GO terms	KEGG pathways	overall affinity score in RT	predicted effect on translation	binding site predictions
PA14_42770	NA	hypothetical protein			-26.91876	repression	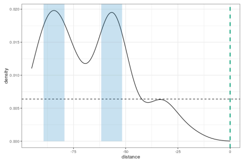
PA14_42870	NA	hypothetical protein			-26.13516	repression	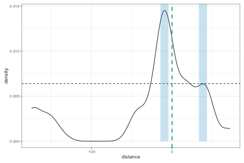
PA14_42910	dotU2	DotU2			-26.91400	no impact	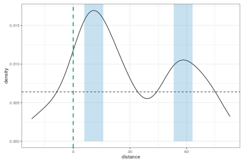
PA14_42950	fha2	Fha2	protein binding		-26.53355	repression	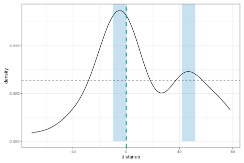
PA14_42980	clpV2	ClpV2	ATP binding, protein metabolic process	Bacterial secretion system, Biofilm formation - Pseudomonas aeruginosa	-26.04647	repression	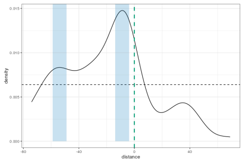
PA14_42990	hsiH2	HsiH2			-27.10569	repression	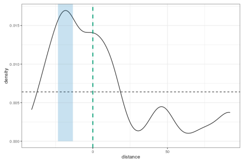
PA14_43040	hsiB2	HsiB2	protein secretion by the type VI secretion system	Biofilm formation - Pseudomonas aeruginosa	-26.45089	repression	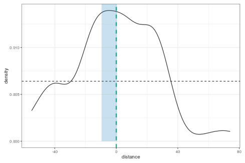
PA14_43220	NA	methyl-accepting chemotaxis transducer	signal transduction, membrane, integral component of membrane	Bacterial chemotaxis, Two-component system	-26.16862	repression	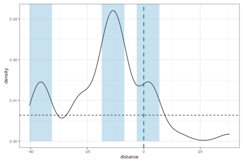
PA14_43270	NA	tRNA 2-selenouridine synthase	transferase activity, transferring selenium-containing groups, tRNA 2-selenouridine synthase activity, tRNA seleno-modification		-25.83102	no impact	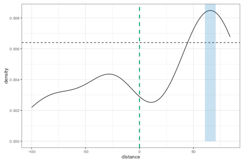
PA14_43405	kdbF	potassium-transporting ATPase subunit F	plasma membrane, potassium transmembrane transporter activity, phosphorylative mechanism, regulation of ATPase activity	Two-component system	-26.11260	repression	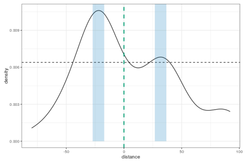

PA14 gene ID	gene name	description	GO terms	KEGG pathways	overall affinity score in RT	predicted effect on translation	binding site predictions
PA14_43420	NA	acyl-CoA dehydrogenase	oxidoreductase activity, acting on the CH-CH group of donors, oxidation-reduction process, acyl-CoA dehydrogenase activity, flavin adenine dinucleotide binding	Caprolactam degradation, Metabolic pathways, Microbial metabolism in diverse environments	-25.97633	repression	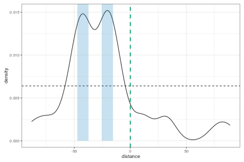
PA14_43460	NA	3-hydroxyacyl-CoA dehydrogenase	3-hydroxyacyl-CoA dehydrogenase activity, fatty acid metabolic process, oxidoreductase activity, oxidation-reduction process	(4Z,7Z,10Z,13Z,16Z)-docosapentaenoate biosynthesis (6-desaturase), (8<i>E</i>,10<i>E</i>)-dodeca-8,10-dienol biosynthesis, (R)- and (S)-3-hydroxybutanoate biosynthesis (engineered), 2-methylpropene degradation, 3-hydroxypropanoate/4-hydroxybutanate cycle, 4-coumarate degradation (aerobic), 4-coumarate degradation (anaerobic), 4-hydroxybenzoate biosynthesis III (plants), <i>Spodoptera littoralis</i> pheromone biosynthesis, alpha-Linolenic acid metabolism, Aminobenzoate degradation, androstenedione degradation, Benzoate degradation, Benzoate degradation, benzoyl-CoA degradation I (aerobic), beta-Alanine metabolism, Butanoate metabolism, Butanoate metabolism, Caprolactam degradation, Carbon fixation pathways in prokaryotes, cholesterol degradation to androstenedione I (cholesterol oxidase), cholesterol degradation to androstenedione II (cholesterol dehydrogenase), crotonate fermentation (to acetate and cyclohexane carboxylate), docosahexaenoate biosynthesis III (6-desaturase, mammals), fatty acid β-oxidation II (peroxisome), Fatty acid degradation, Fatty acid elongation, fatty acid salvage, fermentation to 2-methylbutanoate, Geraniol degradation, glutaryl-CoA degradation, jasmonic acid biosynthesis, Limonene and pinene degradation, Lysine degradation, Metabolic pathways, methyl <i>tert</i>-butyl ether degradation, methyl ketone biosynthesis (engineered), Microbial metabolism in diverse environments, Phenylalanine metabolism, Phenylalanine metabolism, Propanoate metabolism, pyruvate fermentation to butanol I, pyruvate fermentation to butanol II (engineered), pyruvate fermentation to hexanol (engineered), Toluene degradation, Tryptophan metabolism, unsaturated, even numbered fatty acid β-oxidation, Valine, leucine and isoleucine degradation	-26.12471	repression	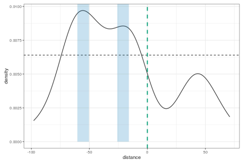
PA14_43760	NA	hypothetical protein			-27.05185	no impact	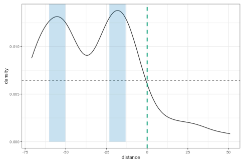
PA14_43820	NA	transcriptional regulator	regulation of transcription, DNA-templated, DNA-binding transcription factor activity, sequence-specific DNA binding, DNA binding		-26.41731	repression	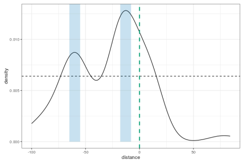
PA14_43870	NA	hypothetical protein			-26.11220	repression	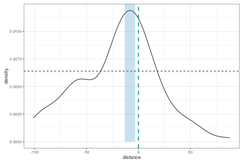
PA14_43880	NA	hypothetical protein			-25.90407	repression	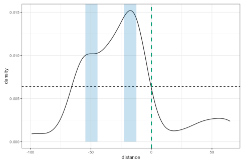
PA14_43910	NA	hypothetical protein			-25.90105	repression	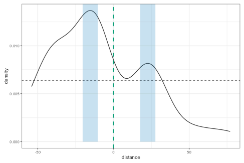
PA14_43920	braB	branched chain amino acid transporter	branched-chain amino acid transmembrane transporter activity, integral component of membrane, branched-chain amino acid transport		-26.73978	repression	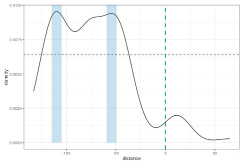

Modeled binding peaks for 1043 predicted targets of RsmA

Modeled binding peaks for 1043 predicted targets of RsmA

PA14 gene ID	gene name	description	GO terms	KEGG pathways	overall affinity score in RT	predicted effect on translation	binding site predictions
PA14_44100	NA	hypothetical protein			-26.59117	repression	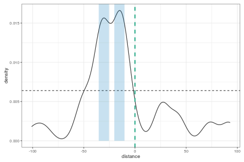
PA14_44120	NA	3-hydroxyisobutyrate dehydrogenase	NADP binding, oxidoreductase activity, oxidation-reduction process, NAD binding		-25.83289	repression	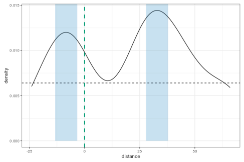
PA14_44130	NA	hypothetical protein	nucleic acid binding		-26.22370	repression	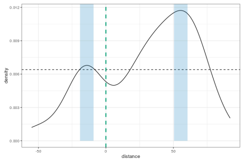
PA14_44150	NA	hypothetical protein			-27.39948	repression	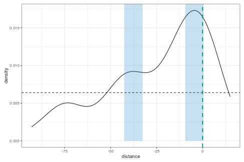
PA14_44160	NA	ATP-NAD kinase	NAD+ kinase activity, NADP biosynthetic process	NAD/NADH phosphorylation and dephosphorylation, NAD/NADP-NADH/NADPH cytosolic interconversion (yeast), NAD/NADP-NADH/NADPH mitochondrial interconversion (yeast), Nicotinate and nicotinamide metabolism	-26.70352	no impact	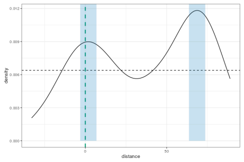
PA14_44340	NA	cbb3-type cytochrome c oxidase subunit I	cytochrome-c oxidase activity, plasma membrane respiratory chain complex IV, oxidation-reduction process, aerobic respiration, integral component of membrane, heme binding	Metabolic pathways, Oxidative phosphorylation, Two-component system	-25.78613	repression	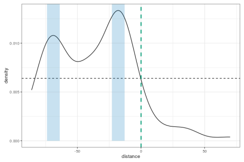
PA14_44440	NA	cation-transporting P-type ATPase	integral component of membrane, metal ion transport, metal ion binding, cation transport, ATPase-coupled cation transmembrane transporter activity, nucleotide binding		-26.17072	repression	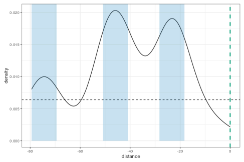
PA14_44510	NA	hypothetical protein			-26.10331	repression	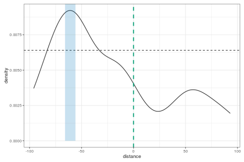
PA14_44640	NA	hypothetical protein			-25.95118	repression	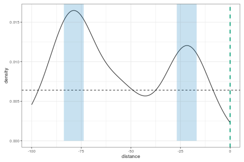
PA14_44670	zipA	cell division protein ZipA	cell septum assembly, integral component of membrane		-26.98139	repression	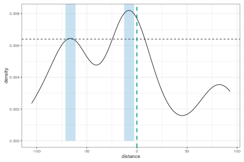

Modeled binding peaks for 1043 predicted targets of RsmA

PA14 gene ID	gene name	description	GO terms	KEGG pathways	overall affinity score in RT	predicted effect on translation	binding site predictions
PA14_44690	NA	GntR family transcriptional regulator	DNA-binding transcription factor activity, regulation of transcription, DNA-templated		-26.46880	no impact	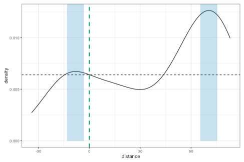
PA14_44700	alkB2	alkane-1 monooxygenase	oxidoreductase activity, lipid metabolic process	Aliphatic compound catabolism, Caprolactam degradation, Fatty acid degradation	-26.94204	repression	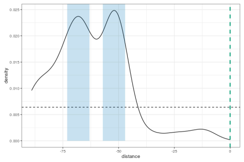
PA14_44890	hcpA	secreted protein Hcp			-27.62500	NA	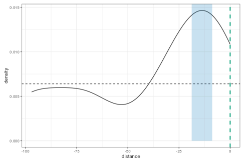
PA14_44900	NA	hypothetical protein			-27.15546	no impact	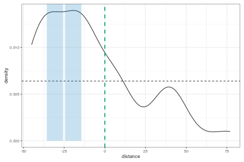
PA14_44910	NA	hypothetical protein			-25.88364	repression	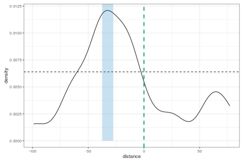
PA14_44920	NA	hypothetical protein			-26.47918	repression	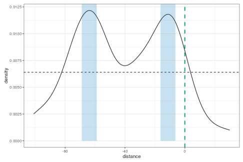
PA14_44930	NA	hypothetical protein			-26.46739	repression	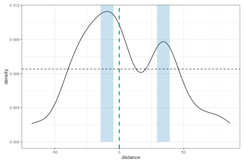
PA14_45130	NA	transporter	integral component of membrane, ethanolamine transmembrane transporter activity, ethanolamine transport		-26.40485	repression	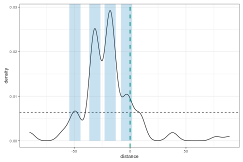
PA14_45250	NA	transcriptional regulator	DNA binding, regulation of transcription, DNA-templated		-26.37498	repression	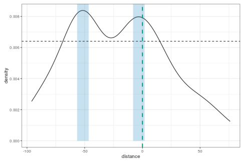
PA14_45470	NA	hypothetical protein	protein binding	Glutathione metabolism	-25.95135	repression	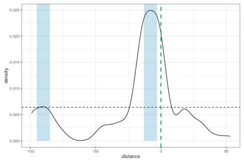

Modeled binding peaks for 1043 predicted targets of RsmA

PA14 gene ID	gene name	description	GO terms	KEGG pathways	overall affinity score in RT	predicted effect on translation	binding site predictions
PA14_45520	NA	plasmid partitioning protein			-26.03872	repression	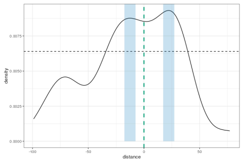
PA14_45560	motC	flagellar motor protein	bacterial-type flagellum-dependent cell motility, bacterial-type flagellum-dependent swarming motility	Bacterial chemotaxis, Flagellar assembly, Two-component system	-26.58162	repression	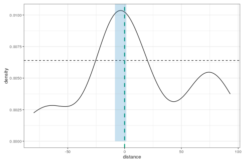
PA14_45580	NA	chemotaxis-specific methylesterase	phosphorelay response regulator activity, phosphorelay signal transduction system, cytoplasm, chemotaxis, protein-glutamate methylesterase activity	Bacterial chemotaxis, Two-component system	-25.96472	repression	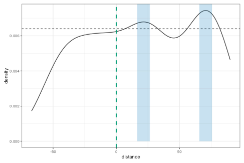
PA14_45610	cheZ	chemotaxis protein CheZ	catalytic activity, bacterial-type flagellum, regulation of chemotaxis, chemotaxis, bacterial-type flagellum-dependent swarming motility	Bacterial chemotaxis	-26.24730	no impact	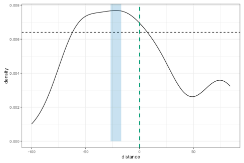
PA14_45640	fleN	flagellar synthesis regulator FleN	ATP binding		-27.02558	repression	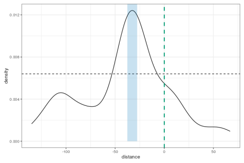
PA14_45700	NA	hypothetical protein			-25.89156	repression	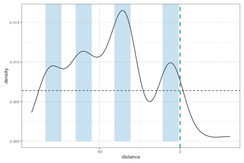
PA14_45720	flhB	flagellar biosynthesis protein FlhB	protein secretion, membrane, protein transport, integral component of membrane, bacterial-type flagellum assembly	Flagellar assembly	-26.68334	no impact	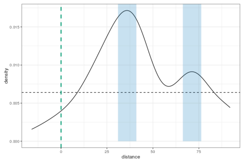
PA14_45790	fliN	flagellar motor switch protein	chemotaxis, bacterial-type flagellum, membrane, bacterial-type flagellum-dependent cell motility, motor activity, bacterial-type flagellum basal body	Bacterial chemotaxis, Flagellar assembly	-26.25479	repression	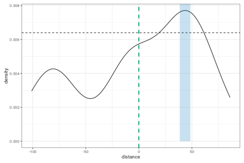
PA14_45810	fliL	flagellar basal body-associated protein FliL	chemotaxis, bacterial-type flagellum basal body, bacterial-type flagellum-dependent cell motility		-26.15769	repression	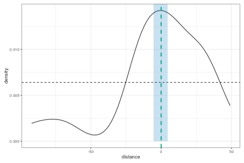
PA14_45880	NA	two-component response regulator	phosphorelay signal transduction system, DNA binding, regulation of transcription, DNA-templated	Two-component system	-26.46480	no impact	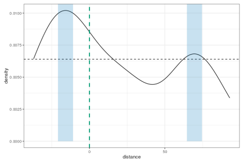

Modeled binding peaks for 1043 predicted targets of RsmA

PA14 gene ID	gene name	description	GO terms	KEGG pathways	overall affinity score in RT	predicted effect on translation	binding site predictions
PA14_45910	NA	RND efflux membrane fusion protein	membrane, transmembrane transporter activity, transmembrane transport		-26.60681	repression	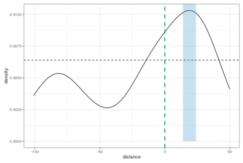
PA14_45950	rsaL	regulatory protein RsaL	quorum sensing, regulation of transcription, DNA-templated, negative regulation of secondary metabolite biosynthetic process, negative regulation of elastin catabolic process, negative regulation of cytolysis in other organism, negative regulation of cell motility, positive regulation of single-species biofilm formation, DNA binding		-25.82502	no impact	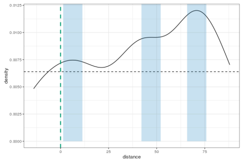
PA14_45960	lasR	transcriptional regulator LasR	regulation of transcription, DNA-templated, DNA binding	Biofilm formation - Pseudomonas aeruginosa, Quorum sensing	-26.54263	repression	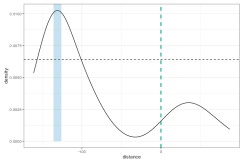
PA14_46010	NA	ABC transporter ATP-binding protein	ATP binding, ATPase activity		-26.20967	repression	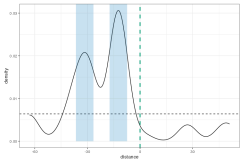
PA14_46160	NA	hypothetical protein			-25.86268	repression	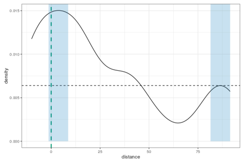
PA14_46250	NA	hypothetical protein	methyltransferase activity		-25.81420	repression	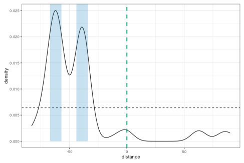
PA14_46290	NA	TetR family transcriptional regulator	DNA binding		-26.06230	no impact	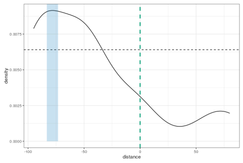
PA14_46340	NA	hypothetical protein			-26.62954	repression	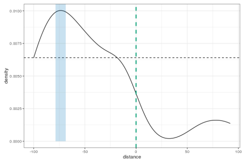
PA14_46370	NA	two-component sensor	phosphorelay signal transduction system, phosphorelay sensor kinase activity, signal transduction, phosphorylation, transferase activity, transferring phosphorus-containing groups		-27.09095	repression	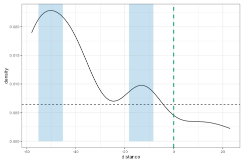
PA14_46420	NA	short chain dehydrogenase	oxidoreductase activity		-25.83749	repression	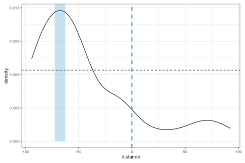

PA14 gene ID	gene name	description	GO terms	KEGG pathways	overall affinity score in RT	predicted effect on translation	binding site predictions
PA14_46480	NA	transcriptional regulator			-25.80681	repression	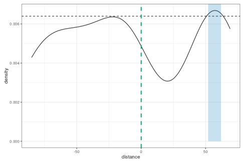
PA14_46490	fabF2	3-oxoacyl-ACP synthase	catalytic activity, fatty acid biosynthetic process, transferase activity, transferring acyl groups other than amino-acyl groups	Biotin metabolism, Fatty acid biosynthesis, Fatty acid metabolism, Metabolic pathways	-25.82721	repression	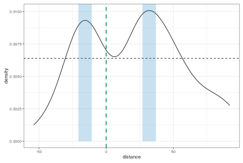
PA14_46650	NA	transmembrane sensor			-26.36611	no impact	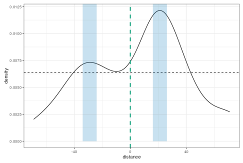
PA14_46670	NA	hypothetical protein			-25.95791	repression	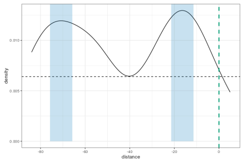
PA14_46710	NA	transcriptional regulator	DNA binding		-27.22564	no impact	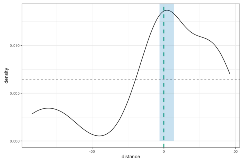
PA14_46760	NA	hypothetical protein			-26.12279	repression	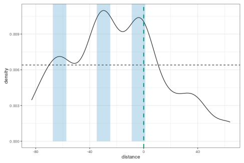
PA14_46770	NA	hypothetical protein			-26.54755	repression	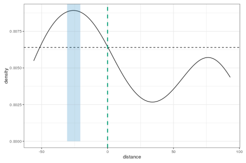
PA14_46930	NA	ABC transporter permease	membrane, transmembrane transport, integral component of membrane, transmembrane transporter activity, ATP-binding cassette (ABC) transporter complex, nitrogen compound transport	ABC transporters, Two-component system	-26.11625	repression	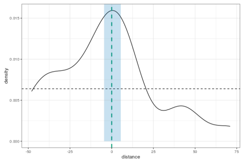
PA14_47010	NA	hypothetical protein			-25.78725	repression	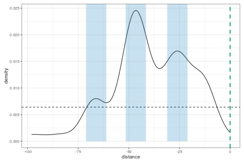
PA14_47040	NA	TerC family protein	integral component of membrane, flavin adenine dinucleotide binding		-26.10806	repression	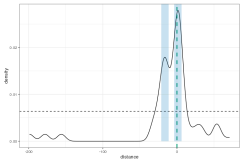

Modeled binding peaks for 1043 predicted targets of RsmA

Modeled binding peaks for 1043 predicted targets of RsmA

PA14 gene ID	gene name	description	GO terms	KEGG pathways	overall affinity score in RT	predicted effect on translation	binding site predictions
PA14_47090	NA	protease	nucleic acid binding, hydrolase activity, metal ion binding, proteolysis, serine-type peptidase activity		-26.71298	repression	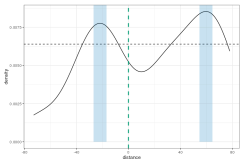
PA14_47140	NA	TonB-dependent receptor	cell outer membrane, siderophore uptake transmembrane transporter activity, siderophore transport, signaling receptor activity		-25.81110	repression	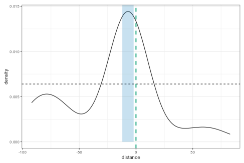
PA14_47190	cyoB	cytochrome o ubiquinol oxidase subunit I	oxidation-reduction process, cytochrome-c oxidase activity, aerobic respiration, integral component of membrane, heme binding, oxidoreductase activity, acting on diphenols and related substances as donors, oxygen as acceptor	Metabolic pathways, Oxidative phosphorylation	-26.40979	repression	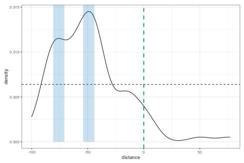
PA14_47210	cyoA	cytochrome o ubiquinol oxidase subunit II	cytochrome o ubiquinol oxidase activity, integral component of membrane, electron transport chain, oxidation-reduction process, cytochrome-c oxidase activity, copper ion binding, membrane, oxidoreductase activity, acting on diphenols and related substances as donors, oxygen as acceptor	Metabolic pathways, Oxidative phosphorylation	-26.01759	repression	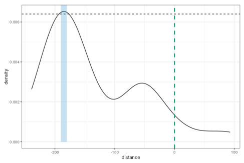
PA14_47320	NA	hypothetical protein			-26.33781	repression	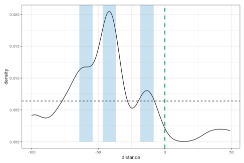
PA14_47330	NA	hypothetical protein			-26.17864	repression	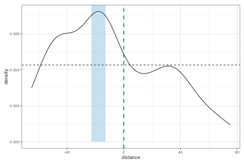
PA14_47370	NA	signal peptidase	proteolysis, serine-type peptidase activity, membrane, integral component of membrane	Protein export, Quorum sensing	-25.92900	repression	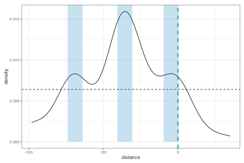
PA14_47390	NA	transmembrane sensor			-26.44576	repression	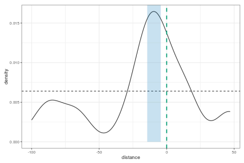
PA14_47400	NA	RNA polymerase ECF-subfamily sigma-70 factor	DNA-binding transcription factor activity, DNA-templated transcription, initiation, regulation of transcription, DNA-templated, DNA binding, sigma factor activity		-27.30230	repression	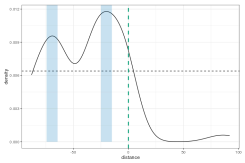
PA14_47500	sseA	3-mercaptopyruvate sulfurtransferase			-26.49995	no impact	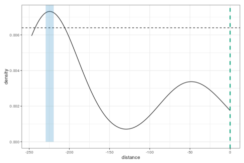

Modeled binding peaks for 1043 predicted targets of RsmA

PA14 gene ID	gene name	description	GO terms	KEGG pathways	overall affinity score in RT	predicted effect on translation	binding site predictions
PA14_47610	NA	transcriptional regulator	DNA binding		-26.98664	repression	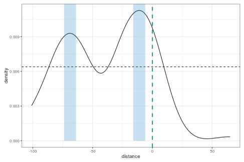
PA14_47650	cobS	cobalamin synthase	cobalamin 5'-phosphate synthase activity, cobalamin biosynthetic process, adenosylcobinamide-GDP ribazoletransferase activity	2-methyladeninyl adenosylcobamide biosynthesis from adenosylcobinamide-GDP, 4-methylphenyl adenosylcobamide biosynthesis from adenosylcobinamide-GDP, 5-hydroxybenzimidazolyl adenosylcobamide biosynthesis from adenosylcobinamide-GDP, 5-methoxy-6-methylbenzimidazolyl adenosylcobamide biosynthesis from adenosylcobinamide-GDP, 5-methoxybenzimidazolyl adenosylcobamide biosynthesis from adenosylcobinamide-GDP, 5-methylbenzimidazolyl adenosylcobamide biosynthesis from adenosylcobinamide-GDP, adeninyl adenosylcobamide biosynthesis from adenosylcobinamide-GDP, adenosylcobalamin biosynthesis from adenosylcobinamide-GDP I, adenosylcobalamin biosynthesis from adenosylcobinamide-GDP II, benzimidazolyl adenosylcobamide biosynthesis from adenosylcobinamide-GDP, Metabolic pathways, phenyl adenosylcobamide biosynthesis from adenosylcobinamide-GDP, Porphyrin and chlorophyll metabolism, Porphyrin and chlorophyll metabolism, superpathway of adenosylcobalamin salvage from cobinamide II	-25.77193	repression	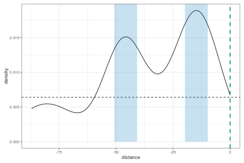
PA14_47690	cobQ	cobyric acid synthase	catalytic activity, cobalamin biosynthetic process	Metabolic pathways, Porphyrin and chlorophyll metabolism	-26.33093	repression	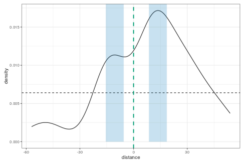
PA14_47720	cobC	threonine-phosphate decarboxylase	catalytic activity, cobalamin biosynthetic process, biosynthetic process, pyridoxal phosphate binding	Metabolic pathways, Porphyrin and chlorophyll metabolism	-26.38774	repression	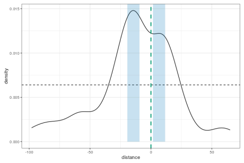
PA14_47760	cobB	cobyrinic acid a,c-diamide synthase	cobalamin biosynthetic process, cobyrinic acid a,c-diamide synthase activity, catalytic activity	Metabolic pathways, Porphyrin and chlorophyll metabolism	-26.00685	repression	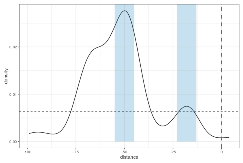
PA14_47790	cobO	cob(I)yrinic acid a,c-diamide adenosyltransferase	ATP binding, cob(I)yrinic acid a,c-diamide adenosyltransferase activity, cobalamin biosynthetic process	Metabolic pathways, Porphyrin and chlorophyll metabolism	-26.43013	no impact	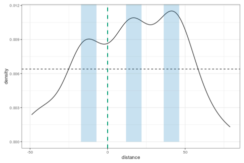
PA14_47960	NA	ABC transporter ATP-binding protein	ATP binding, ATPase activity, amino acid transmembrane transport, ATPase-coupled amino acid transmembrane transporter activity		-26.42819	no impact	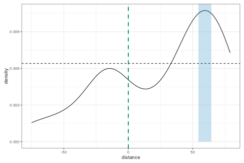
PA14_48115	aprD	alkaline protease secretion protein AprD	ATP binding, integral component of membrane, ATPase-coupled transmembrane transporter activity, transmembrane transport, protein secretion by the type I secretion system, type I protein secretion system complex, ATPase activity	ABC transporters	-26.67465	repression	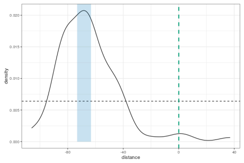
PA14_48140	NA	hypothetical protein			-26.51556	repression	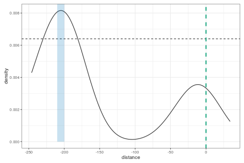

Modeled binding peaks for 1043 predicted targets of RsmA

PA14 gene ID	gene name	description	GO terms	KEGG pathways	overall affinity score in RT	predicted effect on translation	binding site predictions
PA14_48280	NA	multidrug resistance efflux pump			-25.76993	repression	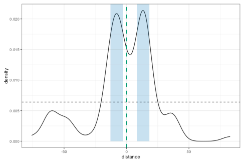
PA14_48300	NA	MFS transporter	integral component of membrane, transmembrane transporter activity, transmembrane transport, integral component of plasma membrane		-25.77971	repression	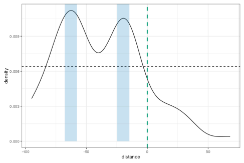
PA14_48330	NA	hypothetical protein			-26.62131	repression	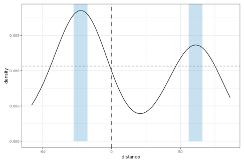
PA14_48830	NA	transcriptional regulator	ATP binding, regulation of transcription, DNA-templated, transcription factor binding, DNA binding, sequence-specific DNA binding		-26.09298	repression	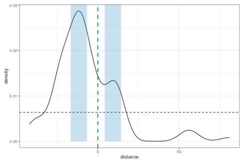
PA14_48850	NA	amino acid permease	membrane, transmembrane transporter activity, transmembrane transport, amino acid transport, integral component of membrane		-25.88486	repression	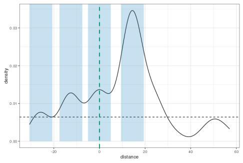
PA14_48880	NA	bacteriophage integrase	DNA binding, DNA recombination, DNA integration		-26.13767	no impact	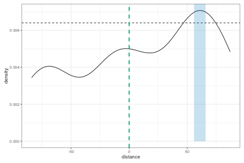
PA14_48890	NA	hypothetical protein			-26.69162	no impact	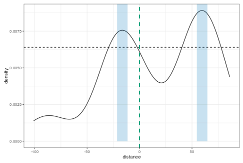
PA14_48920	NA	bacteriophage protein			-25.80865	repression	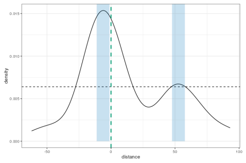
PA14_48970	NA	helix destabilizing protein of bacteriophage Pf1			-25.89847	repression	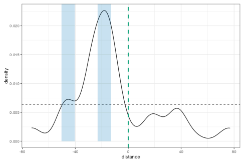
PA14_49000	NA	hypothetical protein			-25.95352	repression	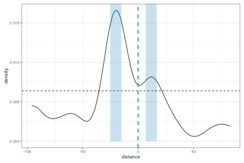

Modeled binding peaks for 1043 predicted targets of RsmA

PA14 gene ID	gene name	description	GO terms	KEGG pathways	overall affinity score in RT	predicted effect on translation	binding site predictions
PA14_49050	NA	hypothetical protein			-26.07349	repression	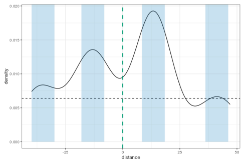
PA14_49070	NA	hypothetical protein	CoA-transferase activity		-26.06063	repression	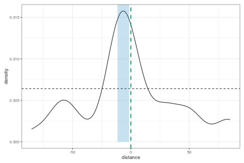
PA14_49130	dctA	C4-dicarboxylate transporter DctA	symporter activity, integral component of membrane, dicarboxylic acid transport	Two-component system	-26.00502	repression	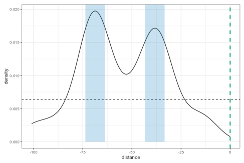
PA14_49210	napE	periplasmic nitrate reductase NapE			-26.51881	no impact	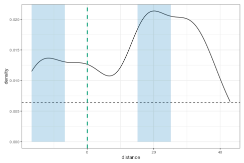
PA14_49280	NA	transglycosylase			-26.03826	repression	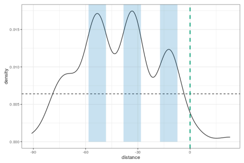
PA14_49420	NA	two-component sensor	signal transduction, integral component of membrane, phosphorylation, transferase activity, transferring phosphorus-containing groups, phosphorelay sensor kinase activity	Two-component system	-26.28780	repression	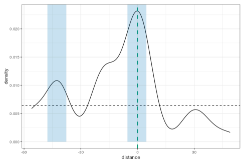
PA14_49440	NA	two-component response regulator	phosphorelay signal transduction system, DNA binding, regulation of transcription, DNA-templated	Two-component system	-25.85214	repression	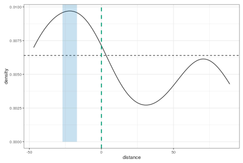
PA14_49500	NA	hypothetical protein			-26.35732	repression	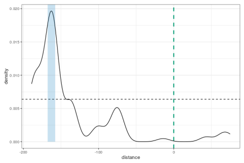
PA14_49700	NA	transcriptional regulator	regulation of transcription, DNA-templated, DNA binding		-26.01341	NA	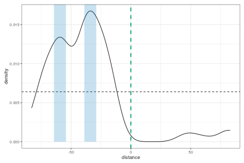
PA14_49740	NA	hypothetical protein			-26.44220	repression	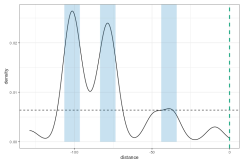

Modeled binding peaks for 1043 predicted targets of RsmA

PA14 gene ID	gene name	description	GO terms	KEGG pathways	overall affinity score in RT	predicted effect on translation	binding site predictions
PA14_49760	rhlC	rhamnosyltransferase 2			-26.82896	repression	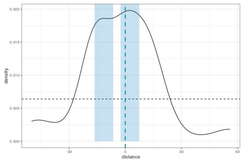
PA14_49810	NA	hypothetical protein			-27.06762	repression	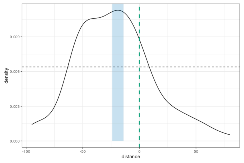
PA14_49840	dgt	deoxyguanosinetriphosphate triphosphohydrolase	triphosphoric monoester hydrolase activity, magnesium ion binding, dGTP catabolic process, dGTPase activity	Purine metabolism, Purine metabolism, Purine metabolism	-27.08635	no impact	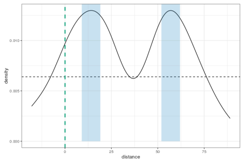
PA14_49850	NA	hypothetical protein			-26.22860	repression	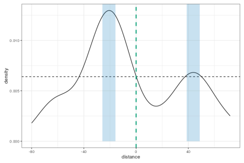
PA14_49870	NA	peptide deformylase			-25.76081	repression	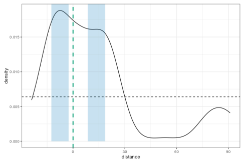
PA14_50020	NA	hypothetical protein			-26.72329	repression	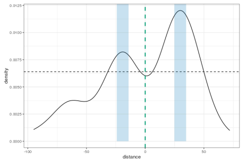
PA14_50080	fliJ	flagellar biosynthesis chaperone	bacterial-type flagellum, bacterial-type flagellum-dependent cell motility, motor activity, chemotaxis, membrane	Flagella assembly , Flagellar assembly	-25.84592	repression	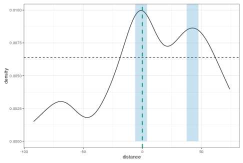
PA14_50240	NA	hypothetical protein			-26.49918	repression	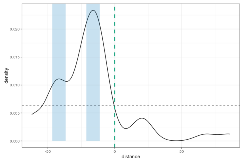
PA14_50330	NA	hypothetical protein			-26.87031	repression	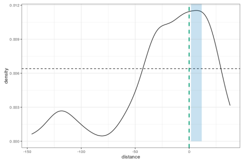
PA14_50560	braG	branched-chain amino acid transport protein BraG	branched-chain amino acid transmembrane transporter activity, branched-chain amino acid transport, ATP binding, ATPase activity	ABC transporters, Quorum sensing	-26.00637	no impact	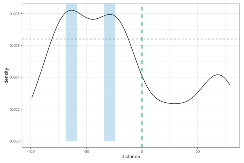

Modeled binding peaks for 1043 predicted targets of RsmA

PA14 gene ID	gene name	description	GO terms	KEGG pathways	overall affinity score in RT	predicted effect on translation	binding site predictions
PA14_50570	NA	hypothetical protein	protein binding		-26.90941	repression	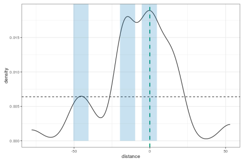
PA14_50590	NA	HSP90 family protein	ATP binding, protein folding, unfolded protein binding		-26.43048	repression	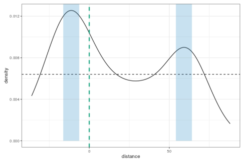
PA14_50600	NA	transcriptional regulator	DNA-binding transcription factor activity, regulation of transcription, DNA-templated		-26.07004	repression	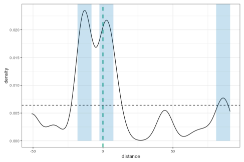
PA14_50620	NA	hypothetical protein			-27.12309	repression	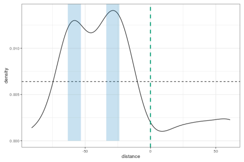
PA14_50650	NA	hypothetical protein	N-acetyltransferase activity		-26.27532	repression	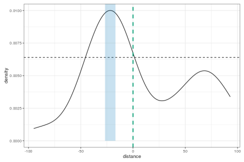
PA14_50830	NA	hypothetical protein			-25.87298	repression	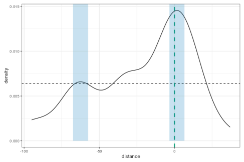
PA14_51000	NA	hypothetical protein			-26.53643	no impact	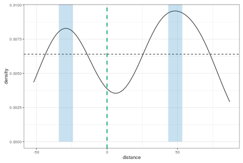
PA14_51040	NA	oxidoreductase	oxidoreductase activity, oxidation-reduction process		-26.23634	repression	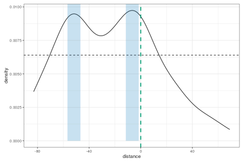
PA14_51080	NA	dioxygenase	catalytic activity, nitronate monooxygenase activity, oxidation-reduction process	Nitrogen metabolism	-26.43890	repression	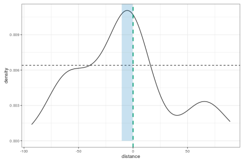
PA14_51150	mucK	cis,cis-muconate transporter MucK	integral component of plasma membrane, transmembrane transport, integral component of membrane, transmembrane transporter activity		-26.35531	repression	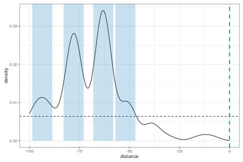

Modeled binding peaks for 1043 predicted targets of RsmA

PA14 gene ID	gene name	description	GO terms	KEGG pathways	overall affinity score in RT	predicted effect on translation	binding site predictions
PA14_51205	NA	transcriptional regulator	DNA binding, regulation of transcription, DNA-templated		-26.20300	repression	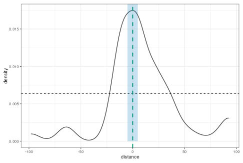
PA14_51340	mvfR	transcriptional regulator MvfR	DNA-binding transcription factor activity, regulation of transcription, DNA-templated, positive regulation of lyase activity, positive regulation of multi-organism process, regulation of transmembrane transport, transcription regulatory region DNA binding, plasma membrane	Biofilm formation - Pseudomonas aeruginosa, Quorum sensing	-26.55724	repression	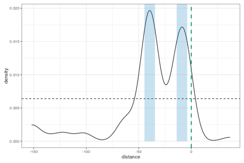
PA14_51360	phnA	anthranilate synthase component I	biosynthetic process, anthranilate synthase activity, phenazine biosynthetic process	4-hydroxy-2(1<i>H</i>)-quinolone biosynthesis, acridone alkaloid biosynthesis, Biofilm formation - Pseudomonas aeruginosa, Biosynthesis of amino acids, Biosynthesis of antibiotics, Biosynthesis of secondary metabolites, Metabolic pathways, Phenazine biosynthesis, Phenylalanine, tyrosine and tryptophan biosynthesis, Phenylalanine, tyrosine and tryptophan biosynthesis, Quorum sensing	-26.38567	repression	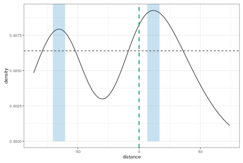
PA14_51390	pqsD	3-oxoacyl-ACP synthase	catalytic activity, 3-oxoacyl-[acyl-carrier-protein] synthase activity, fatty acid biosynthetic process	Biofilm formation - Pseudomonas aeruginosa, Fatty acid biosynthesis (path 1) , Quorum sensing	-26.08746	repression	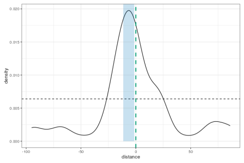
PA14_51420	pqsB	PqsB	catalytic activity, secondary metabolite biosynthetic process	Biofilm formation - Pseudomonas aeruginosa, Quorum sensing	-25.85330	repression	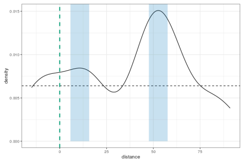
PA14_51440	ogt	methylated-DNA--protein-cysteine methyltransferase	catalytic activity, DNA repair, methylated-DNA-[protein]-cysteine S-methyltransferase activity		-26.20872	no impact	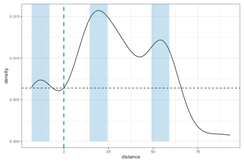
PA14_51450	cupC3	usher CupC3	pilus assembly, fimbrial usher porin activity, membrane, protein binding		-26.82031	repression	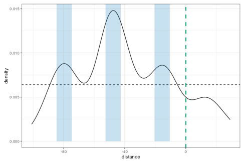
PA14_51480	NA	hypothetical protein	phosphorelay signal transduction system		-26.32230	repression	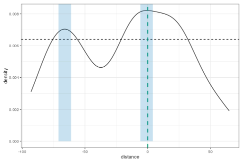
PA14_51500	NA	hypothetical protein			-25.94821	repression	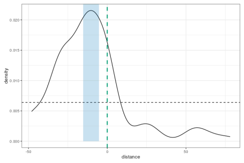
PA14_51810	NA	hypothetical protein			-26.17124	repression	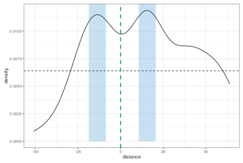

Modeled binding peaks for 1043 predicted targets of RsmA							
PA14 gene ID	gene name	description	GO terms	KEGG pathways	overall affinity score in RT	predicted effect on translation	binding site predictions
PA14_51830	NA	DNA-binding stress protein	oxidoreductase activity, oxidizing metal ions, oxidation-reduction process, cellular iron ion homeostasis, ferric iron binding		-26.76716	repression	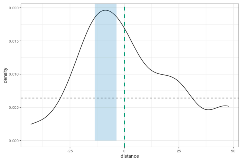
PA14_51850	NA	hypothetical protein			-26.71951	repression	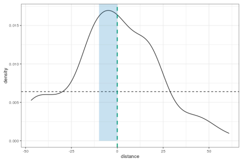
PA14_51890	NA	hypothetical protein			-26.08026	no impact	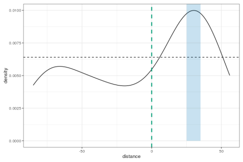
PA14_51920	NA	acylphosphatase			-26.18392	repression	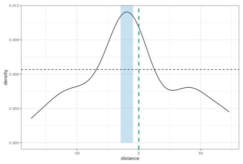
PA14_51930	NA	thioredoxin	cell redox homeostasis, antioxidant activity, oxidoreductase activity, oxidation-reduction process		-26.26459	no impact	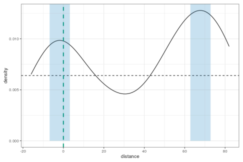
PA14_51940	NA	hypothetical protein			-25.90736	repression	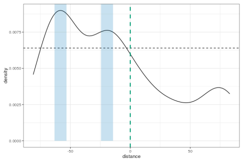
PA14_51960	NA	ribonuclease			-26.14785	repression	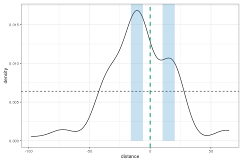
PA14_52040	purM	phosphoribosylaminoimidazole synthetase	phosphoribosylformylglycinamidine cyclo-ligase activity, 'de novo' IMP biosynthetic process	Biosynthesis of antibiotics, Biosynthesis of secondary metabolites, Metabolic pathways, Purine metabolism, Purine metabolism	-25.75832	no impact	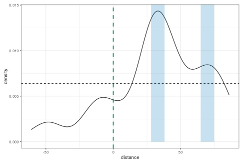
PA14_52060	NA	hypothetical protein			-26.18165	repression	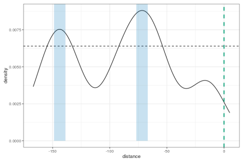
PA14_52090	NA	hypothetical protein			-26.71398	repression	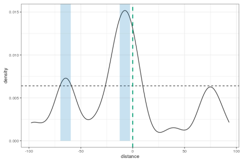

Modeled binding peaks for 1043 predicted targets of RsmA

PA14 gene ID	gene name	description	GO terms	KEGG pathways	overall affinity score in RT	predicted effect on translation	binding site predictions
PA14_52180	relA	GTP pyrophosphokinase	guanosine tetraphosphate metabolic process	Purine metabolism	-25.77444	repression	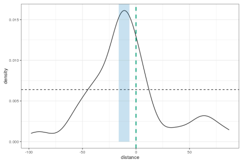
PA14_52260	NA	sensor/response regulator hybrid	signal transduction, integral component of membrane, phosphorelay signal transduction system, phosphorelay sensor kinase activity, phosphorylation, transferase activity, transferring phosphorus-containing groups, positive regulation of cell motility, positive regulation of secondary metabolite biosynthetic process	Biofilm formation - Pseudomonas aeruginosa, Two-component system	-26.99472	repression	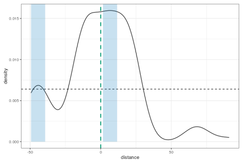
PA14_52330	NA	hypothetical protein			-27.00334	repression	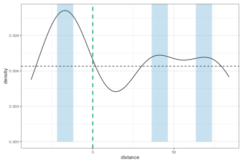
PA14_52340	NA	hypothetical protein			-26.31841	no impact	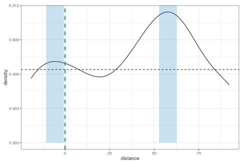
PA14_52380	NA	cytochrome b561	electron transfer activity, integral component of membrane, membrane, respiratory electron transport chain		-26.16503	repression	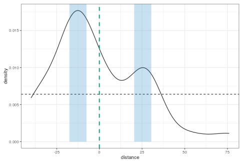
PA14_52400	kup	potassium uptake protein Kup	potassium ion transmembrane transporter activity, membrane, potassium ion transmembrane transport		-26.41985	no impact	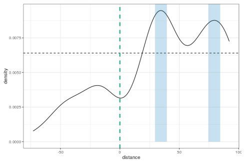
PA14_52460	mgtE	Mg transporter MgtE	cation transport, cation transmembrane transporter activity, magnesium ion transmembrane transporter activity, magnesium ion transport, membrane		-25.76921	NA	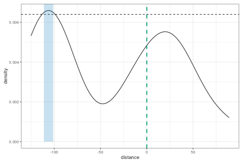
PA14_52480	NA	hypothetical protein			-26.33184	repression	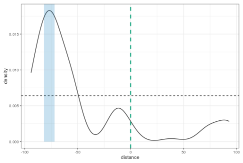
PA14_52520	NA	hypothetical protein			-26.09445	repression	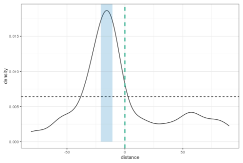
PA14_52570	rsmA	carbon storage regulator	RNA binding, regulation of carbohydrate metabolic process, mRNA catabolic process	Biofilm formation - Pseudomonas aeruginosa, Two-component system	-26.17486	no impact	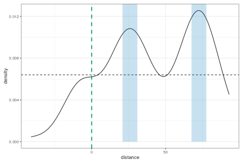

Modeled binding peaks for 1043 predicted targets of RsmA

PA14 gene ID	gene name	description	GO terms	KEGG pathways	overall affinity score in RT	predicted effect on translation	binding site predictions
PA14_52580	lysC	aspartate kinase	aspartate kinase activity, cellular amino acid biosynthetic process, lysine biosynthetic process via diaminopimelate	2-Oxocarboxylic acid metabolism, Biosynthesis of amino acids, Biosynthesis of antibiotics, Biosynthesis of secondary metabolites, Cysteine and methionine metabolism, Glycine, serine and threonine metabolism, Glycine, serine and threonine metabolism;Lysine biosynthesis , Lysine biosynthesis, Metabolic pathways, Microbial metabolism in diverse environments, Monobactam biosynthesis	-26.56291	no impact	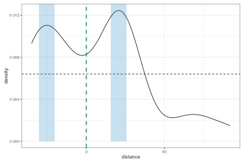
PA14_52670	astD	succinylglutamic semialdehyde dehydrogenase	oxidoreductase activity, oxidation-reduction process, arginine catabolic process, succinylglutamate-semialdehyde dehydrogenase activity, oxidoreductase activity, acting on the aldehyde or oxo group of donors, NAD or NADP as acceptor	Arginine and proline metabolism, Arginine and proline metabolism, Arginine and proline metabolism; Butanoate metabolism; Glutamate metabolism, Metabolic pathways	-25.95964	repression	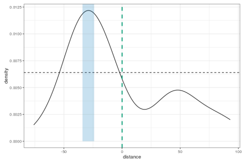
PA14_52700	aruF	arginine/ornithine succinyltransferase AI subunit	arginine catabolic process, arginine N-succinyltransferase activity	Arginine and proline metabolism, Metabolic pathways	-26.19102	repression	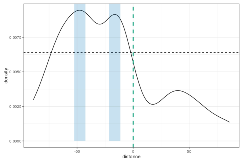
PA14_52730	NA	hypothetical protein			-26.60771	no impact	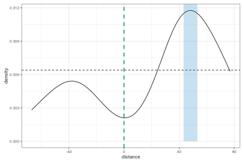
PA14_52900	NA	acyl-CoA dehydrogenase	oxidoreductase activity, acting on the CH-CH group of donors, flavin adenine dinucleotide binding, oxidation-reduction process, acyl-CoA dehydrogenase activity	beta-Alanine metabolism, Biosynthesis of antibiotics, Biosynthesis of secondary metabolites, Carbon metabolism, Fatty acid degradation, Fatty acid metabolism, Metabolic pathways, Propanoate metabolism, Valine, leucine and isoleucine degradation	-26.00531	repression	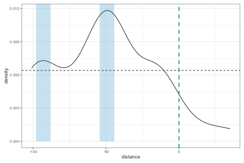
PA14_52920	NA	transcriptional regulator	DNA-binding transcription factor activity, regulation of transcription, DNA-templated		-26.25181	no impact	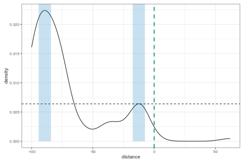
PA14_52930	NA	transcriptional regulator	DNA-binding transcription factor activity, regulation of transcription, DNA-templated		-26.06311	repression	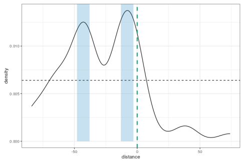
PA14_53140	NA	hypothetical protein	regulation of transcription, DNA-templated		-26.74931	repression	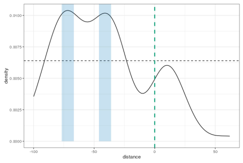
PA14_53360	plcH	hemolytic phospholipase C	hydrolase activity, acting on ester bonds, phosphatidylcholine phospholipase C activity, phospholipase C activity, lipid catabolic process, catalytic activity	2-arachidonoylglycerol biosynthesis, Biosynthesis of secondary metabolites, Ether lipid metabolism, Ether lipid metabolism, Glycerophospholipid metabolism, Glycerophospholipid metabolism, Inositol phosphate metabolism, Inositol phosphate metabolism, Metabolic pathways, plasmalogen biosynthesis, plasmalogen degradation, Quorum sensing	-26.03698	repression	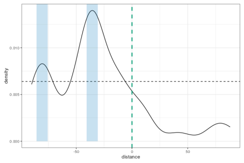
PA14_53420	NA	glutathione peroxidase	glutathione peroxidase activity, response to oxidative stress, oxidation-reduction process	Arachidonic acid metabolism, Glutathione metabolism	-26.35472	no impact	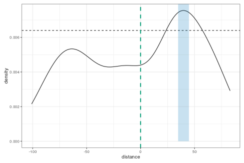

Modeled binding peaks for 1043 predicted targets of RsmA

PA14 gene ID	gene name	description	GO terms	KEGG pathways	overall affinity score in RT	predicted effect on translation	binding site predictions
PA14_53550	NA	transcriptional regulator	DNA binding		-26.60532	no impact	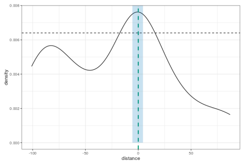
PA14_53620	NA	hypothetical protein			-27.27390	repression	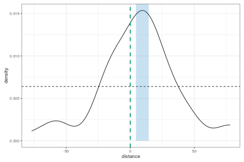
PA14_53650	NA	hypothetical protein	hydrolase activity, acting on ester bonds		-26.35420	no impact	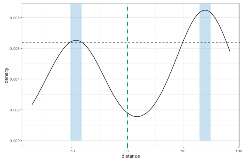
PA14_53660	NA	hypothetical protein			-26.04535	repression	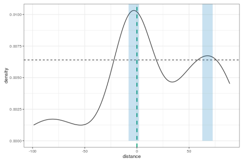
PA14_53720	NA	transcriptional regulator	DNA-binding transcription factor activity, regulation of transcription, DNA-templated		-25.82646	repression	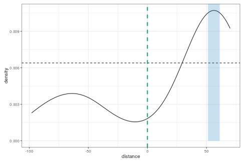
PA14_53770	NA	hypothetical protein	transferase activity, L-lysine catabolic process to acetate, catalytic activity		-26.84553	repression	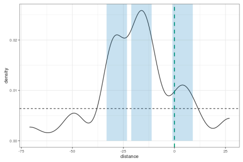
PA14_53890	NA	hypothetical protein			-26.08598	repression	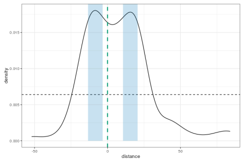
PA14_54150	putP	sodium/proline symporter PutP	proline:sodium symporter activity, sodium ion transport, proline transport, integral component of membrane, sodium ion binding, membrane, transmembrane transporter activity, transmembrane transport, proline catabolic process to glutamate, L-proline transmembrane transporter activity		-26.90802	repression	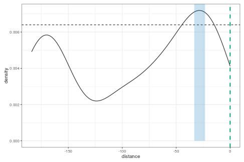
PA14_54240	NA	hypothetical protein			-25.95856	repression	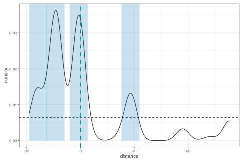
PA14_54400	mucC	positive regulator for alginate biosynthesis MucC	regulation of polysaccharide biosynthetic process		-26.24973	no impact	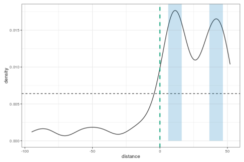

Modeled binding peaks for 1043 predicted targets of RsmA

PA14 gene ID	gene name	description	GO terms	KEGG pathways	overall affinity score in RT	predicted effect on translation	binding site predictions
PA14_54430	algU	RNA polymerase sigma factor AlgU	DNA binding, DNA-binding transcription factor activity, DNA-templated transcription, initiation, regulation of transcription, DNA-templated, sigma factor activity, negative regulation of bacterial-type flagellum-dependent cell motility, regulation of polysaccharide biosynthetic process		-26.12164	no impact	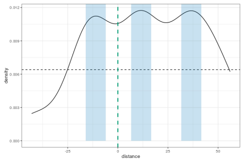
PA14_54450	nadB	L-aspartate oxidase	oxidoreductase activity, oxidation-reduction process, L-aspartate oxidase activity, NAD biosynthetic process	Alanine, aspartate and glutamate metabolism, Metabolic pathways, Nicotinate and nicotinamide metabolism	-25.75042	repression	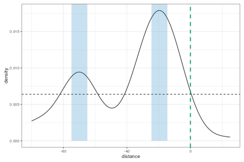
PA14_54510	NA	two-component response regulator	phosphorelay signal transduction system, DNA binding, regulation of transcription, DNA-templated	Two-component System, Two-component system	-26.15022	no impact	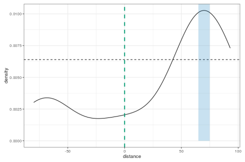
PA14_54590	ung	uracil-DNA glycosylase	uracil DNA N-glycosylase activity, DNA repair, base-excision repair, hydrolase activity, hydrolyzing N-glycosyl compounds	Base excision repair	-26.09117	no impact	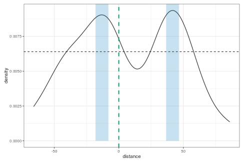
PA14_54670	NA	3-hydroxyisobutyrate dehydrogenase	3-hydroxyisobutyrate dehydrogenase activity, NAD binding, oxidation-reduction process, oxidoreductase activity, NADP binding	Valine, leucine and isoleucine degradation	-25.82852	no impact	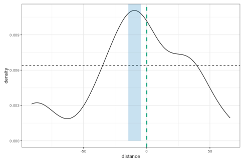
PA14_54760	NA	hypothetical protein			-25.90424	repression	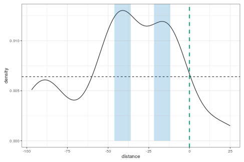
PA14_55100	NA	hypothetical protein			-27.04477	no impact	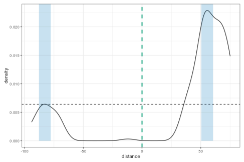
PA14_55170	cat	chloramphenicol acetyltransferase			-26.91880	repression	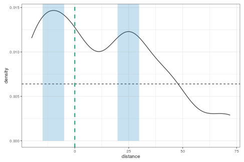
PA14_55320	NA	hypothetical protein			-26.00816	repression	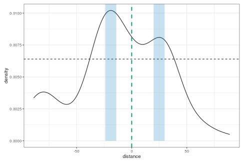
PA14_55390	NA	hypothetical protein			-26.31723	repression	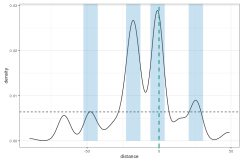

Modeled binding peaks for 1043 predicted targets of RsmA

PA14 gene ID	gene name	description	GO terms	KEGG pathways	overall affinity score in RT	predicted effect on translation	binding site predictions
PA14_55490	hxcT	HxcT	type II protein secretion system complex, protein secretion by the type II secretion system	Bacterial secretion system	-26.47400	repression	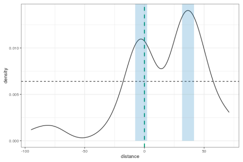
PA14_55550	NA	ECF subfamily RNA polymerase sigma-70 factor	DNA-binding transcription factor activity, DNA-templated transcription, initiation, regulation of transcription, DNA-templated, DNA binding, sigma factor activity		-27.36791	repression	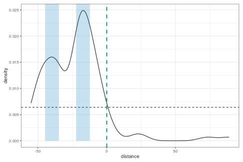
PA14_55760	NA	hypothetical protein			-26.25671	no impact	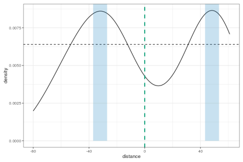
PA14_55770	NA	phosphate transporter	inorganic phosphate transmembrane transporter activity, phosphate ion transport, membrane		-25.90061	repression	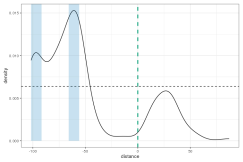
PA14_55790	NA	hypothetical protein			-25.79495	no impact	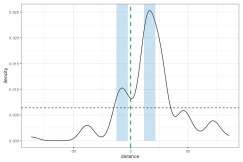
PA14_55800	NA	hypothetical protein	aspartic-type endopeptidase activity, membrane	Type II secretion system	-26.01556	repression	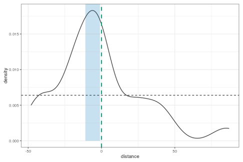
PA14_55820	NA	hypothetical protein			-26.61864	repression	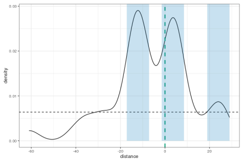
PA14_55850	NA	pilus assembly protein	protein binding		-25.98150	no impact	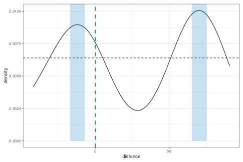
PA14_55920	NA	type II secretion system protein	protein secretion		-26.32353	repression	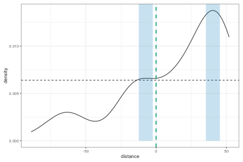
PA14_55930	NA	pilus assembly protein			-26.42236	repression	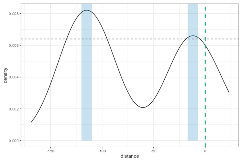

Modeled binding peaks for 1043 predicted targets of RsmA

PA14 gene ID	gene name	description	GO terms	KEGG pathways	overall affinity score in RT	predicted effect on translation	binding site predictions
PA14_56000	pctA	chemotactic transducer PctA	transmembrane signaling receptor activity, chemotaxis, signal transduction, membrane, integral component of membrane	Bacterial chemotaxis, Two-component system	-26.89068	repression	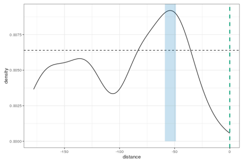
PA14_56070	mvaT	transcriptional regulator MvaT, P16 subunit			-26.29723	no impact	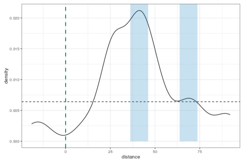
PA14_56380	NA	hypothetical protein			-26.03489	repression	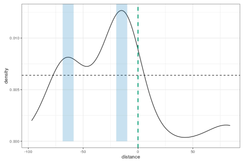
PA14_56430	NA	transcriptional regulator	DNA binding, regulation of transcription, DNA-templated		-25.90179	repression	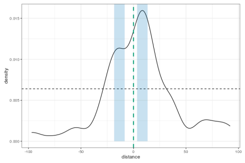
PA14_56510	NA	hypothetical protein			-26.10623	repression	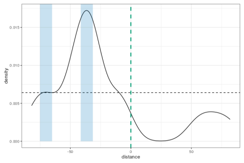
PA14_56600	NA	hypothetical protein	fatty acid biosynthetic process, [acyl-carrier-protein] phosphodiesterase activity		-26.32993	no impact	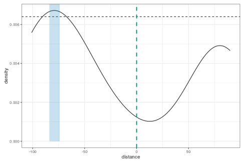
PA14_56620	NA	hypothetical protein	DNA-binding transcription factor activity, regulation of transcription, DNA-templated, regulation of single-species biofilm formation on inanimate substrate, transcription regulatory region DNA binding		-25.97000	repression	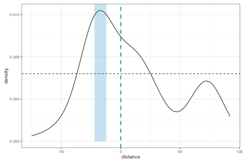
PA14_56720	NA	oxidoreductase	catalytic activity, coenzyme binding		-26.96950	repression	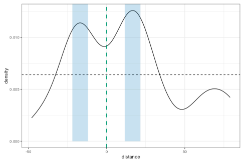
PA14_56890	NA	multidrug efflux protein	membrane, transmembrane transporter activity, transmembrane transport	beta-Lactam resistance, Cationic antimicrobial peptide (CAMP) resistance	-26.04431	repression	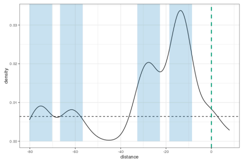
PA14_56940	NA	two-component sensor	phosphorelay sensor kinase activity, signal transduction, integral component of membrane, phosphorylation, transferase activity, transferring phosphorus-containing groups		-26.02488	repression	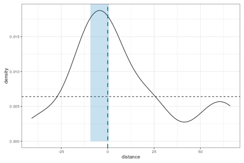

Modeled binding peaks for 1043 predicted targets of RsmA

PA14 gene ID	gene name	description	GO terms	KEGG pathways	overall affinity score in RT	predicted effect on translation	binding site predictions
PA14_57030	fxsA	FxsA protein	membrane		-26.22241	repression	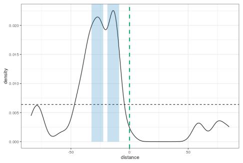
PA14_57040	NA	hypothetical protein	cofactor binding		-26.11177	repression	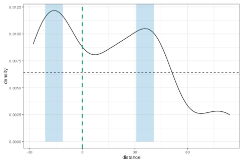
PA14_57050	fabG	3-ketoacyl-ACP reductase			-26.37681	no impact	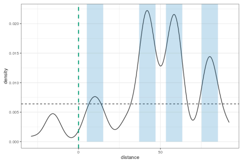
PA14_57080	NA	hypothetical protein	catalytic activity, DNA repair		-27.28387	repression	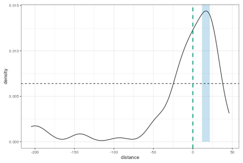
PA14_57110	NA	hypothetical protein	membrane, transmembrane transport		-27.19536	repression	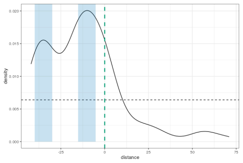
PA14_57140	NA	two-component response regulator	phosphorelay signal transduction system		-26.59798	repression	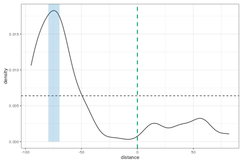
PA14_57180	NA	hypothetical protein			-26.03844	repression	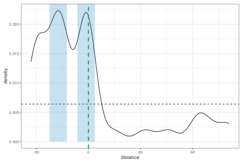
PA14_57240	NA	hypothetical protein			-26.90621	repression	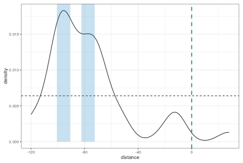
PA14_57275	ftsZ	cell division protein FtsZ	GTP binding, GTPase activity		-26.37723	repression	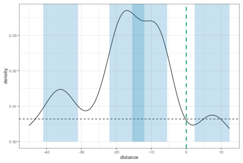
PA14_57320	ddl	D-alanine--D-alanine ligase	cytoplasm, D-alanine-D-alanine ligase activity, ATP binding, metal ion binding	D-Alanine metabolism, D-Alanine metabolism, Metabolic pathways, Peptidoglycan biosynthesis, Peptidoglycan biosynthesis, UDP-<i>N</i>-acetylmuramoyl-pentapeptide biosynthesis I (<i>meso</i>-diaminopimelate containing), UDP-<i>N</i>-acetylmuramoyl-pentapeptide biosynthesis II (lysine-containing), UDP-<i>N</i>-acetylmuramoyl-pentapeptide biosynthesis III (<i>meso</i>-diaminopimelate containing), Vancomycin resistance	-25.91337	repression	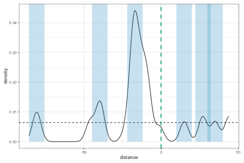

Modeled binding peaks for 1043 predicted targets of RsmA

PA14 gene ID	gene name	description	GO terms	KEGG pathways	overall affinity score in RT	predicted effect on translation	binding site predictions
PA14_57340	murG	UDPdiphospho-muramoylpentapeptide beta-N-acetylglucosaminyltransferase	transferase activity, transferring hexosyl groups, undecaprenyldiphospho-muramoylpentapeptide beta-N-acetylglucosaminyltransferase activity, carbohydrate metabolic process, lipid glycosylation	Metabolic pathways, Peptidoglycan biosynthesis, Vancomycin resistance	-26.11866	repression	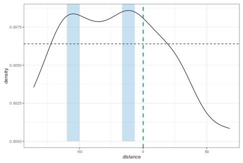
PA14_57490	NA	hypothetical protein	nucleic acid binding, nuclease activity		-26.95879	repression	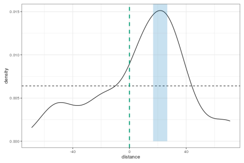
PA14_57520	sspB	ClpXP protease specificity-enhancing factor			-26.06874	repression	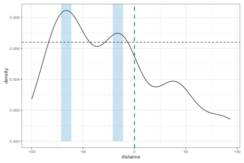
PA14_57540	NA	cytochrome c1	electron transfer activity, heme binding	Metabolic pathways, Oxidative phosphorylation, Two-component system	-26.20564	repression	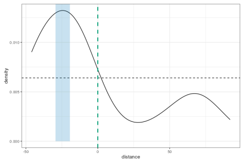
PA14_57710	cysN	bifunctional sulfate adenylyltransferase subunit 1/adenylylsulfate kinase	GTPase activity, GTP binding, sulfate assimilation, adenylylsulfate kinase activity, ATP binding, sulfur compound metabolic process, cellular response to sulfate starvation	Biosynthesis of antibiotics, Metabolic pathways, Microbial metabolism in diverse environments, Monobactam biosynthesis, Purine metabolism, Selenocompound metabolism, Sulfur metabolism	-26.27893	repression	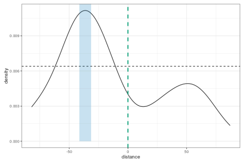
PA14_57740	NA	hypothetical protein			-26.00524	repression	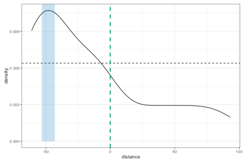
PA14_57810	murA	UDP-N-acetylglucosamine 1-carboxyvinyltransferase	transferase activity, transferring alkyl or aryl (other than methyl) groups, UDP-N-acetylglucosamine 1-carboxyvinyltransferase activity, UDP-N-acetylgalactosamine biosynthetic process, catalytic activity	Amino sugar and nucleotide sugar metabolism, Metabolic pathways, Peptidoglycan biosynthesis	-26.16469	repression	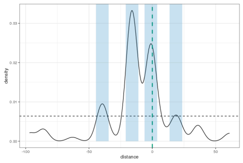
PA14_57830	NA	hypothetical protein			-26.29156	repression	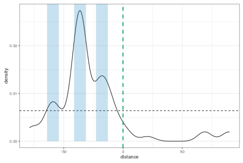
PA14_57880	NA	ABC transporter ATP-binding protein	ATP binding, ATPase activity	ABC transporters	-27.20033	repression	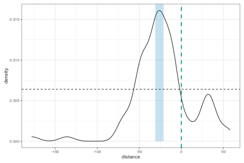
PA14_58090	NA	hypothetical protein			-25.79988	no impact	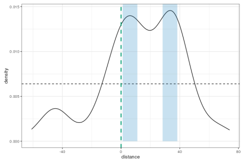

Modeled binding peaks for 1043 predicted targets of RsmA

PA14 gene ID	gene name	description	GO terms	KEGG pathways	overall affinity score in RT	predicted effect on translation	binding site predictions
PA14_58100	cafA	cytoplasmic axial filament protein	RNA binding, ribonuclease activity, RNA processing, nucleic acid binding		-26.50096	no impact	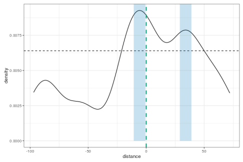
PA14_58300	NA	two-component response regulator	phosphorelay signal transduction system, sequence-specific DNA binding, transcription regulatory region DNA binding	Two-component system	-25.99365	repression	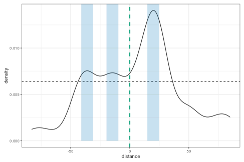
PA14_58375	mdpA	metallopeptidase MdpA	hydrolase activity, peptide catabolic process		-25.85669	no impact	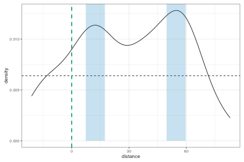
PA14_58390	dppA3	dipeptide ABC transporter substrate-binding protein DppA3	ATP-binding cassette (ABC) transporter complex, transmembrane transport, dipeptide transport, dipeptide transport, peptide binding	ABC transporters, Bacterial chemotaxis	-26.34292	repression	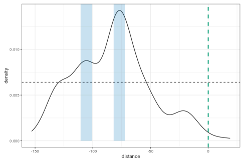
PA14_58570	NA	outer membrane ferric siderophore receptor	cell outer membrane, siderophore uptake transmembrane transporter activity, siderophore transport, signaling receptor activity		-26.24695	repression	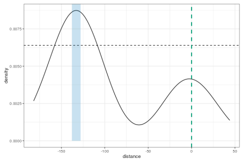
PA14_58800	NA	hypothetical protein			-26.56649	repression	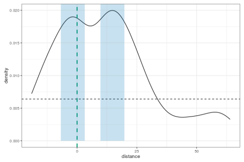
PA14_58890	NA	hemolysin activation/secretion protein			-25.87944	repression	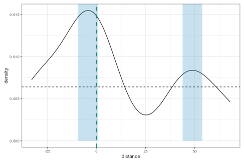
PA14_60190	clpB	clpB protein	protein metabolic process, ATP binding, cytoplasm, response to heat, protein refolding		-26.39608	repression	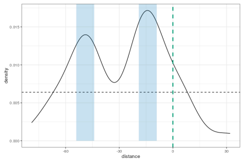
PA14_60230	comL	competence protein ComL	protein binding		-26.48308	repression	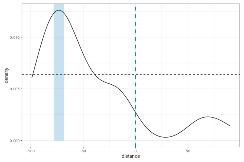
PA14_60410	NA	hypothetical protein			-26.14572	repression	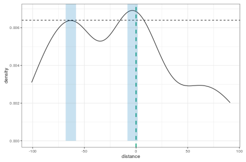

Modeled binding peaks for 1043 predicted targets of RsmA							
PA14 gene ID	gene name	description	GO terms	KEGG pathways	overall affinity score in RT	predicted effect on translation	binding site predictions
PA14_60490	NA	cytochrome c	electron transfer activity, heme binding		-27.04017	repression	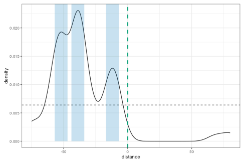
PA14_60520	NA	hypothetical protein			-25.93051	no impact	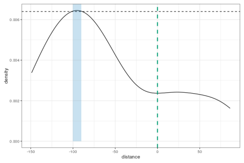
PA14_60540	NA	hypothetical protein			-27.86924	no impact	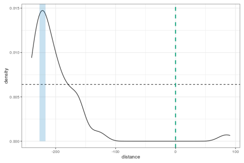
PA14_60590	NA	hypothetical protein			-26.24540	no impact	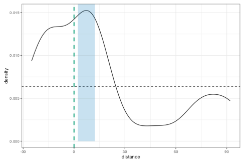
PA14_60630	NA	hypothetical protein	membrane		-26.73767	repression	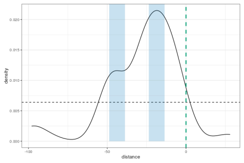
PA14_60650	NA	hypothetical protein	RNA processing, RNA ligase activity		-26.23403	repression	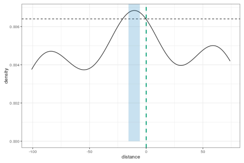
PA14_60660	NA	nucleotidyltransferase			-26.86003	no impact	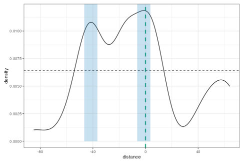
PA14_60670	rtcA	RNA 3'-terminal-phosphate cyclase	RNA processing, RNA-3'-phosphate cyclase activity, catalytic activity		-26.70312	repression	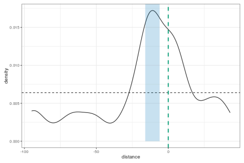
PA14_60790	NA	ABC transporter ATP-binding protein	ATP binding, ATPase activity		-27.09936	no impact	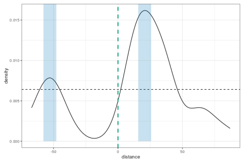
PA14_60810	NA	transcriptional regulator NfxB	DNA binding		-26.05989	NA	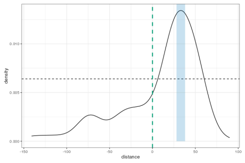

Modeled binding peaks for 1043 predicted targets of RsmA

PA14 gene ID	gene name	description	GO terms	KEGG pathways	overall affinity score in RT	predicted effect on translation	binding site predictions
PA14_60850	mexC	multidrug efflux RND membrane fusion protein	membrane, transmembrane transporter activity, transmembrane transport		-25.93127	no impact	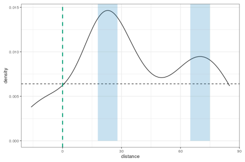
PA14_60930	NA	hypothetical protein			-26.03655	no impact	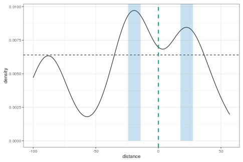
PA14_60960	NA	hypothetical protein			-26.96288	repression	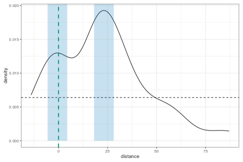
PA14_60990	radA	DNA repair protein RadA	damaged DNA binding, ATP binding, DNA repair, DNA binding, DNA-dependent ATPase activity		-25.85632	no impact	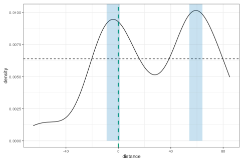
PA14_61050	mscL	large-conductance mechanosensitive channel	mechanosensitive ion channel activity, integral component of membrane, ion transmembrane transport		-26.49816	repression	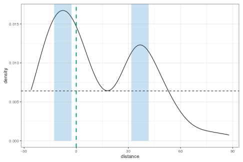
PA14_61190	NA	hypothetical protein			-25.95880	no impact	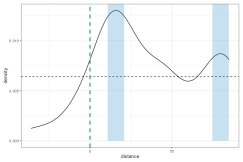
PA14_61200	NA	hypothetical protein			-25.92076	repression	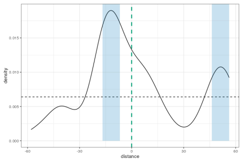
PA14_61260	NA	hypothetical protein			-25.97979	repression	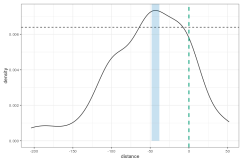
PA14_61270	NA	hypothetical protein	polyketide metabolic process		-25.92757	no impact	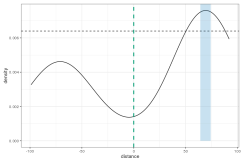
PA14_61450	NA	hypothetical protein			-26.52770	repression	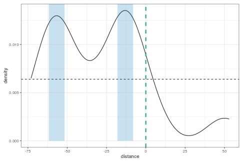

Modeled binding peaks for 1043 predicted targets of RsmA

PA14 gene ID	gene name	description	GO terms	KEGG pathways	overall affinity score in RT	predicted effect on translation	binding site predictions
PA14_61480	uraA	uracil permease	membrane, transmembrane transporter activity, transmembrane transport		-26.11963	repression	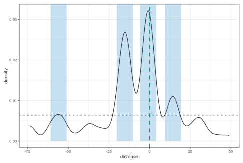
PA14_61590	NA	hypothetical protein	catalytic activity, coenzyme binding		-26.56598	no impact	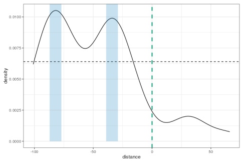
PA14_61680	prmC	S-adenosylmethionine-dependent methyltransferase, PrmC	methyltransferase activity, nucleic acid binding, methylation, protein methylation, protein methyltransferase activity, S-adenosylmethionine-dependent methyltransferase activity		-26.09569	repression	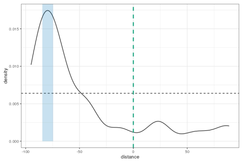
PA14_61880	rimI	peptide n-acetyltransferase RimI	N-terminal protein amino acid acetylation, acetyltransferase activity, N-acetyltransferase activity		-25.85148	repression	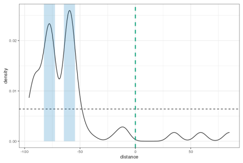
PA14_61920	NA	hypothetical protein			-25.78273	repression	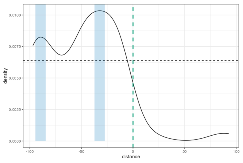
PA14_61950	NA	hypothetical protein			-26.32781	repression	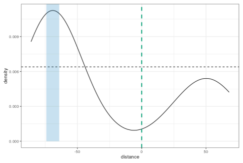
PA14_61980	NA	hypothetical protein			-26.25335	repression	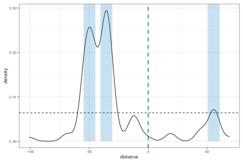
PA14_62100	NA	sulfite oxidase subunit YedZ	integral component of plasma membrane, protein repair		-26.17245	repression	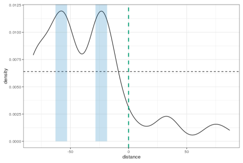
PA14_62120	pssA	phosphatidylserine synthase	phospholipid biosynthetic process, membrane, phosphotransferase activity, for other substituted phosphate groups	Biosynthesis of secondary metabolites, Glycerophospholipid metabolism, Glycine, serine and threonine metabolism, Metabolic pathways	-26.15956	no impact	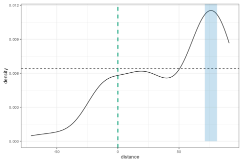
PA14_62180	NA	hypothetical protein			-26.45452	repression	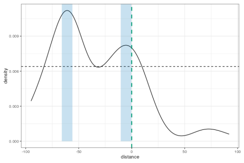

Modeled binding peaks for 1043 predicted targets of RsmA								
PA14 gene ID	gene name	description	GO terms	KEGG pathways	overall affinity score in RT	predicted effect on translation	binding site predictions
PA14_62360	NA	Rieske family iron-sulfur cluster-binding protein	oxidoreductase activity, 2 iron, 2 sulfur cluster binding, oxidation-reduction process		-26.20957	repression	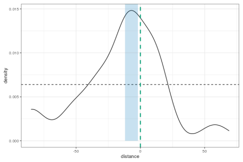
PA14_62370	NA	hypothetical protein			-26.54000	repression	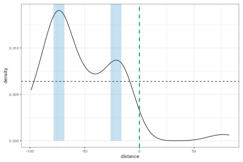
PA14_62380	NA	hypothetical protein			-26.68019	no impact	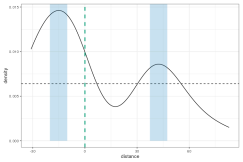
PA14_62420	NA	hypothetical protein			-26.09600	no impact	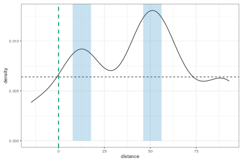
PA14_62440	NA	transporter	membrane, transmembrane transporter activity, transmembrane transport, nucleobase transmembrane transporter activity		-26.18425	repression	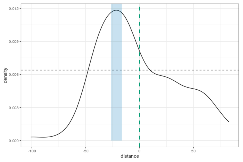
PA14_62650	NA	hypothetical protein			-26.43604	repression	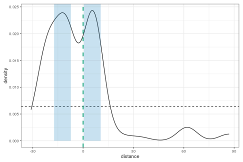
PA14_62660	NA	hypothetical protein			-26.32516	repression	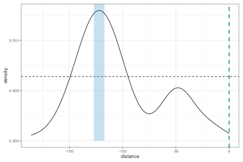
PA14_62810	secG	preprotein translocase subunit SecG	protein secretion, P-P-bond-hydrolysis-driven protein transmembrane transporter activity, integral component of membrane	Bacterial secretion system, Protein export, Quorum sensing	-26.54087	no impact	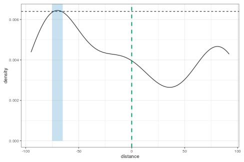
PA14_62840	glmM	phosphoglucosamine mutase	carbohydrate metabolic process, intramolecular transferase activity, phosphotransferases, magnesium ion binding, organic substance metabolic process, phosphoglucosamine mutase activity, phosphomannomutase activity, phosphoglucomutase activity, peptidoglycan biosynthetic process	Amino sugar and nucleotide sugar metabolism, Biosynthesis of antibiotics, Metabolic pathways	-26.22505	repression	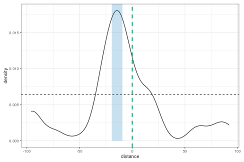
PA14_62860	ftsH	cell division protein FtsH	metalloendopeptidase activity, ATP binding, proteolysis, membrane, zinc ion binding, integral component of membrane, cellular response to antibiotic		-26.31823	repression	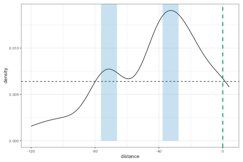

Modeled binding peaks for 1043 predicted targets of RsmA

PA14 gene ID	gene name	description	GO terms	KEGG pathways	overall affinity score in RT	predicted effect on translation	binding site predictions
PA14_62970	dnaK	molecular chaperone DnaK	ATP binding, protein folding, unfolded protein binding	RNA degradation	-26.70614	repression	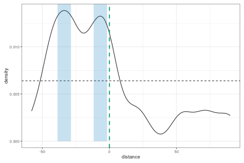
PA14_63010	recN	DNA repair protein RecN	ATP binding, DNA repair, DNA recombination		-26.73269	repression	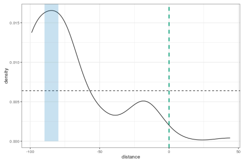
PA14_63070	NA	GntR family transcriptional regulator	DNA-binding transcription factor activity, regulation of transcription, DNA-templated		-26.37493	repression	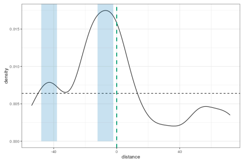
PA14_63150	pmrA	two-component response regulator	phosphorelay signal transduction system, DNA binding, regulation of transcription, DNA-templated	Quorum sensing, Two-component system	-26.02368	no impact	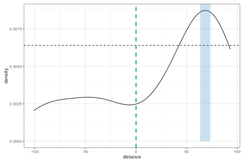
PA14_63210	NA	two-component response regulator	phosphorelay signal transduction system		-26.55538	repression	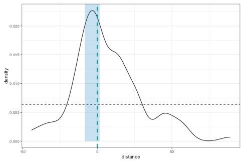
PA14_63430	NA	hypothetical protein			-26.99795	repression	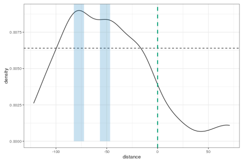
PA14_63470	NA	methyltransferase	methyltransferase activity		-26.09983	repression	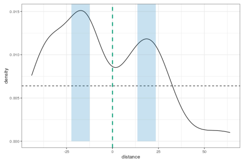
PA14_63480	NA	amino acid permease	membrane, transmembrane transporter activity, transmembrane transport	Quorum sensing	-25.87824	repression	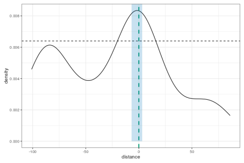
PA14_63850	lpd3	dihydrolipoamide dehydrogenase	oxidoreductase activity, oxidation-reduction process, electron transfer activity, cell redox homeostasis, flavin adenine dinucleotide binding, dihydrolipoyl dehydrogenase activity, oxidoreductase activity, acting on a sulfur group of donors, NAD(P) as acceptor	Biosynthesis of antibiotics, Biosynthesis of secondary metabolites, Carbon metabolism, Citrate cycle (TCA cycle), Glycine, serine and threonine metabolism, Glycolysis / Gluconeogenesis, Glyoxylate and dicarboxylate metabolism, Metabolic pathways, Microbial metabolism in diverse environments, Propanoate metabolism, Pyruvate metabolism, Valine, leucine and isoleucine degradation	-26.72344	repression	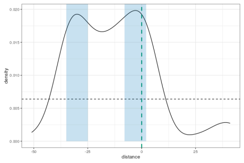
PA14_63990	speA	arginine decarboxylase	catalytic activity, arginine catabolic process, spermidine biosynthetic process, arginine decarboxylase activity, putrescine biosynthetic process from arginine	Arginine and proline metabolism, Metabolic pathways	-26.41367	repression	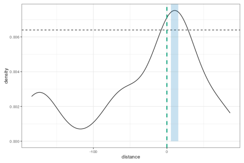

Modeled binding peaks for 1043 predicted targets of RsmA

PA14 gene ID	gene name	description	GO terms	KEGG pathways	overall affinity score in RT	predicted effect on translation	binding site predictions
PA14_64030	NA	hypothetical protein			-26.75446	repression	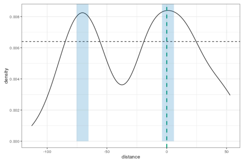
PA14_64280	NA	branched-chain amino acid ABC transporter permease	membrane, transmembrane transporter activity, transmembrane transport	ABC transporters	-26.31395	repression	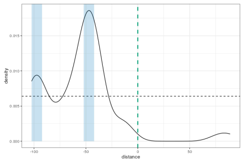
PA14_64290	NA	ABC transporter permease	membrane, transmembrane transporter activity, transmembrane transport	ABC transporters	-26.50258	repression	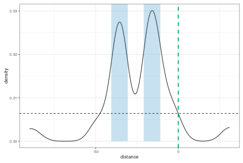
PA14_64310	NA	ABC transporter ATP-binding protein	ATP binding, ATPase activity	ABC transporters	-25.89234	repression	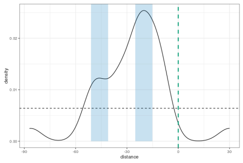
PA14_64540	NA	hypothetical protein	nitrate assimilation		-26.68686	repression	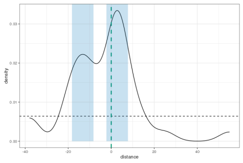
PA14_64690	NA	transmembrane sensor			-26.02169	no impact	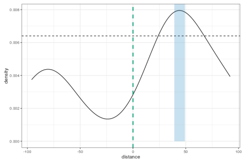
PA14_64790	NA	MFS transporter	integral component of membrane, transmembrane transporter activity, transmembrane transport, integral component of plasma membrane		-25.86214	repression	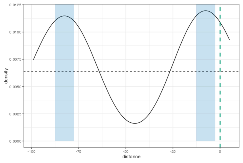
PA14_64860	NA	ABC transporter ATP-binding protein	ATP binding, ATPase activity, branched-chain amino acid transmembrane transporter activity, branched-chain amino acid transport	ABC transporters, Quorum sensing	-25.90276	no impact	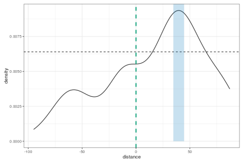
PA14_64870	NA	ABC transporter ATP-binding protein	ATP binding, ATPase activity	ABC transporters, Quorum sensing	-25.82008	repression	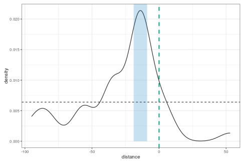
PA14_64890	NA	branched chain amino acid ABC transporter permease	membrane, transmembrane transporter activity, transmembrane transport	ABC transporters, Quorum sensing	-26.07133	repression	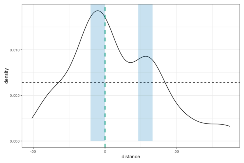

Modeled binding peaks for 1043 predicted targets of RsmA

PA14 gene ID	gene name	description	GO terms	KEGG pathways	overall affinity score in RT	predicted effect on translation	binding site predictions
PA14_64900	NA	ABC transporter substrate-binding protein	amino acid transport	ABC transporters, Quorum sensing	-26.79778	repression	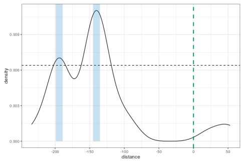
PA14_64930	NA	hypothetical protein	hydrolase activity		-26.41814	repression	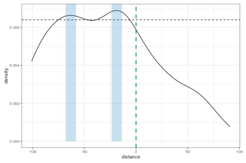
PA14_65040	NA	hypothetical protein	membrane, transmembrane transport		-26.98324	repression	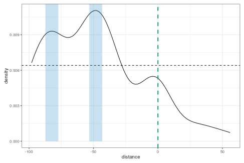
PA14_65150	rplI	50S ribosomal protein L9	structural constituent of ribosome, ribosome, translation	Ribosome	-26.32846	repression	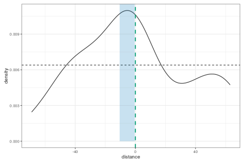
PA14_65230	purA	adenylosuccinate synthetase	GTP binding, adenylosuccinate synthase activity, purine nucleotide biosynthetic process	Alanine, aspartate and glutamate metabolism, Metabolic pathways, Purine metabolism	-26.94784	repression	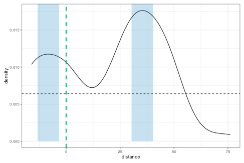
PA14_65250	hisZ	ATP phosphoribosyltransferase	histidine biosynthetic process, cytoplasm	Biosynthesis of amino acids, Biosynthesis of secondary metabolites, Histidine metabolism, Metabolic pathways	-26.48545	no impact	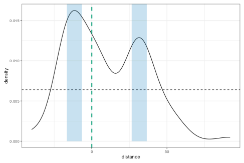
PA14_65270	hflC	protease subunit HflC	membrane, integral component of membrane, regulation of peptidase activity		-26.00770	repression	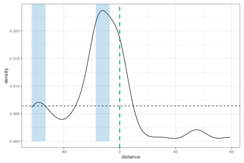
PA14_65300	hflX	GTP-binding protein	GTP binding		-26.08092	no impact	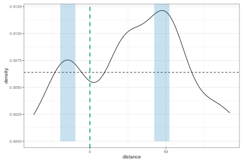
PA14_65310	hfq	RNA-binding protein Hfq	RNA binding, regulation of transcription, DNA-templated	Quorum sensing, RNA degradation	-26.44463	repression	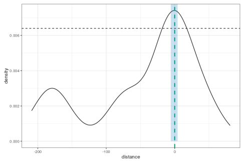
PA14_65370	amiB	N-acetylmuramoyl-L-alanine amidase	N-acetylmuramoyl-L-alanine amidase activity, peptidoglycan catabolic process	Cationic antimicrobial peptide (CAMP) resistance	-26.47295	repression	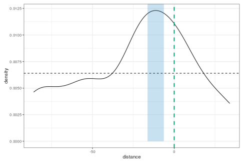

Modeled binding peaks for 1043 predicted targets of RsmA

PA14 gene ID	gene name	description	GO terms	KEGG pathways	overall affinity score in RT	predicted effect on translation	binding site predictions
PA14_65390	NA	hypothetical protein	ADP-dependent NAD(P)H-hydrate dehydratase activity	NADH repair	-27.08775	repression	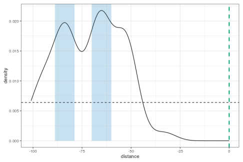
PA14_65500	psd	phosphatidylserine decarboxylase	phosphatidylserine decarboxylase activity, phospholipid biosynthetic process	Biosynthesis of secondary metabolites, Glycerophospholipid metabolism, Metabolic pathways	-25.78809	repression	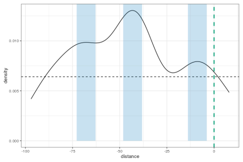
PA14_65740	thiC	thiamine biosynthesis protein ThiC	thiamine biosynthetic process, iron-sulfur cluster binding, carbon-carbon lyase activity	Metabolic pathways, Thiamine metabolism	-25.91016	repression	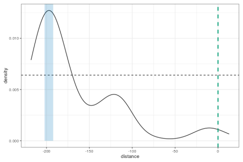
PA14_65795	NA	hypothetical protein	magnesium ion binding, thiamine pyrophosphate binding, catalytic activity	Arginine and proline metabolism, Metabolic pathways	-26.33738	no impact	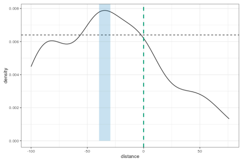
PA14_65820	NA	acyl-CoA dehydrogenase	acyl-CoA dehydrogenase activity, oxidation-reduction process, oxidoreductase activity, acting on the CH-CH group of donors, flavin adenine dinucleotide binding		-26.37244	no impact	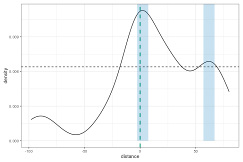
PA14_65900	NA	TetR family transcriptional regulator	DNA binding		-26.25507	repression	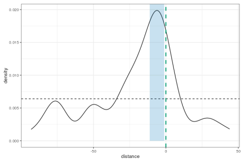
PA14_65970	NA	transcriptional regulator	DNA-binding transcription factor activity, regulation of transcription, DNA-templated		-26.51420	repression	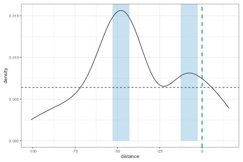
PA14_66110	NA	glycosyl transferase family protein			-26.56124	repression	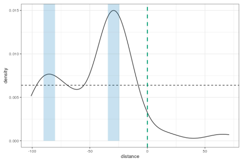
PA14_66120	NA	hypothetical protein			-26.01147	repression	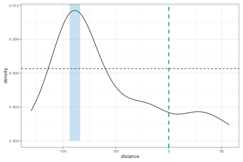
PA14_66160	NA	glycosyl transferase family protein			-26.22993	repression	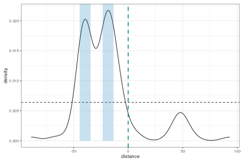

Modeled binding peaks for 1043 predicted targets of RsmA

PA14 gene ID	gene name	description	GO terms	KEGG pathways	overall affinity score in RT	predicted effect on translation	binding site predictions
PA14_66210	NA	hypothetical protein	protein kinase activity, protein phosphorylation		-26.40993	repression	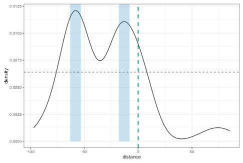
PA14_66250	waaF	heptosyltransferase II	lipopolysaccharide biosynthetic process, transferase activity, transferring glycosyl groups	Lipopolysaccharide biosynthesis, Metabolic pathways	-26.36280	repression	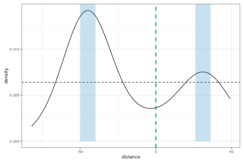
PA14_66340	NA	hypothetical protein	rRNA methyltransferase activity, rRNA base methylation		-25.91521	no impact	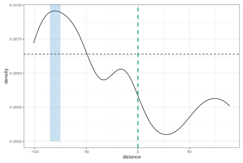
PA14_66620	pilQ	type 4 fimbrial biogenesis outer membrane protein PilQ precursor	outer membrane, protein secretion		-26.43861	repression	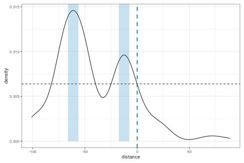
PA14_66640	pilO	type 4 fimbrial biogenesis protein PilO	type IV pilus-dependent motility, type IV pilus biogenesis		-26.45195	repression	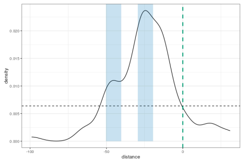
PA14_66690	NA	protease	metalloendopeptidase activity, proteolysis		-26.59438	repression	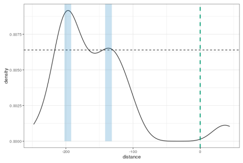
PA14_66840	phaC2	poly(3-hydroxyalkanoic acid) synthase 2	poly-hydroxybutyrate biosynthetic process, transferase activity, transferring acyl groups	Butanoate metabolism	-26.08728	repression	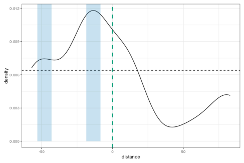
PA14_66890	NA	hypothetical protein			-26.07996	no impact	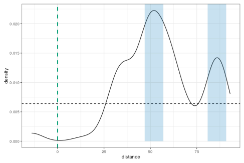
PA14_66970	tatB	sec-independent translocase	membrane, protein transport by the Tat complex, protein transport	Bacterial secretion system, Protein export	-25.81061	repression	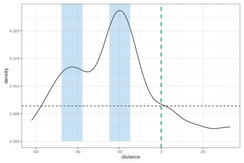
PA14_67110	NA	prolyl aminopeptidase	proteolysis, peptidase activity, aminopeptidase activity, cytoplasm	Arginine and proline metabolism	-25.81514	repression	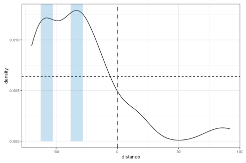

Modeled binding peaks for 1043 predicted targets of RsmA

PA14 gene ID	gene name	description	GO terms	KEGG pathways	overall affinity score in RT	predicted effect on translation	binding site predictions
PA14_67170	NA	LysR family transcriptional regulator	DNA-binding transcription factor activity, regulation of transcription, DNA-templated		-25.98541	repression	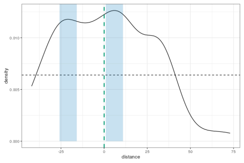
PA14_67300	NA	ABC transporter substrate-binding protein	transmembrane transporter activity, ATP-binding cassette (ABC) transporter complex, transmembrane transport	ABC transporters	-26.70841	repression	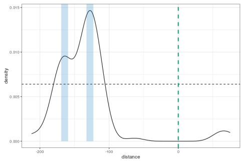
PA14_67310	NA	amino acid permease	amino acid transport, integral component of membrane, transmembrane transport, membrane, transmembrane transporter activity		-26.57136	repression	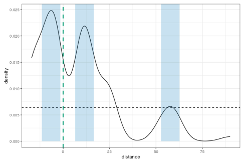
PA14_67530	NA	hypothetical protein			-26.40825	repression	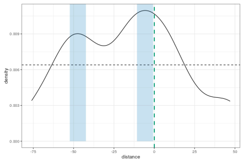
PA14_67540	NA	hypothetical protein			-26.14696	no impact	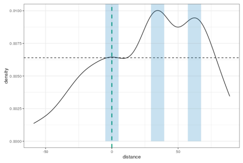
PA14_67550	NA	transcriptional regulator	DNA binding, regulation of transcription, DNA-templated		-25.80983	repression	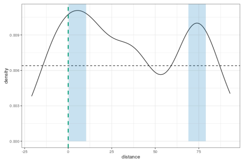
PA14_67620	NA	hypothetical protein			-26.00664	repression	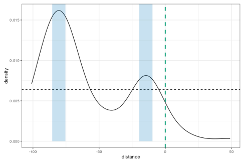
PA14_67700	NA	hypothetical protein			-25.92493	repression	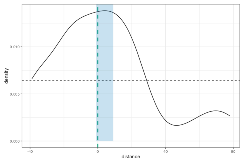
PA14_67770	pgm	phosphoglyceromutase	catalytic activity, phosphoglycerate mutase activity, cytoplasm, glucose catabolic process, manganese ion binding, metal ion binding	1-butanol autotrophic biosynthesis (engineered), Biosynthesis of amino acids, Biosynthesis of antibiotics, Biosynthesis of secondary metabolites, Carbon metabolism, Entner-Doudoroff pathway III (semi-phosphorylative), ethylene biosynthesis V (engineered), gluconeogenesis II (<i>Methanobacterium thermoautotrophicum</i>), glycerol degradation to butanol, Glycine, serine and threonine metabolism, Glycine, serine and threonine metabolism, Glycolysis / Gluconeogenesis, Glycolysis / Gluconeogenesis, glycolysis II (from fructose 6-phosphate), glycolysis IV (plant cytosol), Metabolic pathways, Methane metabolism, Methane metabolism, Microbial metabolism in diverse environments, photosynthetic 3-hydroxybutanoate biosynthesis (engineered), Rubisco shunt, superpathway of glucose and xylose degradation	-26.11933	repression	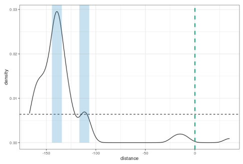
PA14_67840	NA	ABC-type amino acid transporter			-25.93803	repression	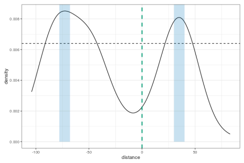

Modeled binding peaks for 1043 predicted targets of RsmA

PA14 gene ID	gene name	description	GO terms	KEGG pathways	overall affinity score in RT	predicted effect on translation	binding site predictions
PA14_67990	mutY	A/G-specific adenine glycosylase	base-excision repair, DNA binding, catalytic activity, DNA N-glycosylase activity, hydrolase activity, DNA repair	Base excision repair	-26.32967	repression	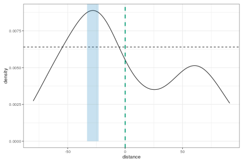
PA14_68040	NA	short-chain dehydrogenase	oxidoreductase activity		-26.28830	repression	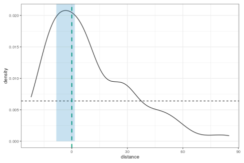
PA14_68110	NA	transcriptional regulator	DNA-binding transcription factor activity, regulation of transcription, DNA-templated		-25.78841	repression	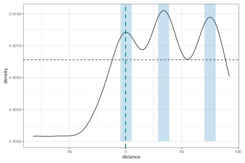
PA14_68200	rmlA	glucose-1-phosphate thymidylyltransferase	glucose-1-phosphate thymidylyltransferase activity, extracellular polysaccharide biosynthetic process, biosynthetic process, nucleotidyltransferase activity	Acarbose and validamycin biosynthesis, Biosynthesis of antibiotics, Metabolic pathways, Polyketide sugar unit biosynthesis, Streptomycin biosynthesis	-26.04083	repression	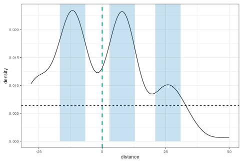
PA14_68340	arcB	ornithine carbamoyltransferase	cellular amino acid metabolic process, amino acid binding, carboxyl- or carbamoyltransferase activity, ornithine metabolic process, ornithine carbamoyltransferase activity, arginine deiminase pathway	Arginine and proline metabolism; Urea cycle and metabolism of amino groups, Arginine biosynthesis, Biosynthesis of amino acids, Biosynthesis of antibiotics, Biosynthesis of secondary metabolites, Metabolic pathways	-25.85066	repression	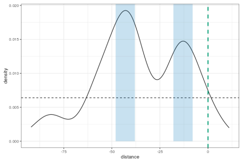
PA14_68380	nudE	ADP-ribose diphosphatase NudE	hydrolase activity	Purine metabolism	-25.92373	repression	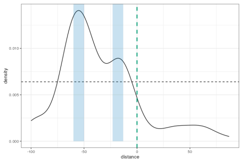
PA14_68400	NA	LysM domain/BON superfamily protein			-27.58552	repression	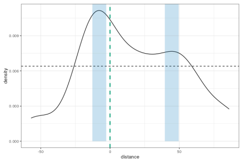
PA14_68470	NA	hypothetical protein	RNA binding, regulation of carbohydrate metabolic process, mRNA catabolic process		-26.85627	repression	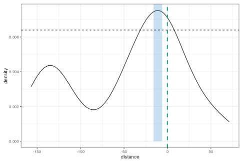
PA14_68500	NA	iron-containing alcohol dehydrogenase	oxidoreductase activity, metal ion binding, oxidation-reduction process		-25.87900	repression	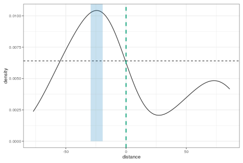
PA14_68570	NA	hypothetical protein			-26.37132	repression	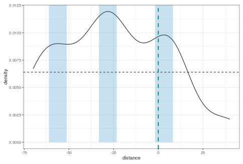

Modeled binding peaks for 1043 predicted targets of RsmA

PA14 gene ID	gene name	description	GO terms	KEGG pathways	overall affinity score in RT	predicted effect on translation	binding site predictions
PA14_68620	NA	hypothetical protein			-26.61808	repression	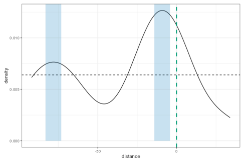
PA14_68830	NA	hypothetical protein			-26.66519	repression	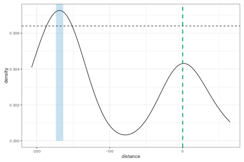
PA14_68870	gcvT	glycine cleavage system aminomethyltransferase T	aminomethyltransferase activity, glycine catabolic process, protein binding	Biosynthesis of antibiotics, Biosynthesis of secondary metabolites, Carbon metabolism, Glycine, serine and threonine metabolism, Glycine, serine and threonine metabolism, Glyoxylate and dicarboxylate metabolism, Metabolic pathways, One carbon pool by folate, One carbon pool by folate	-25.78242	repression	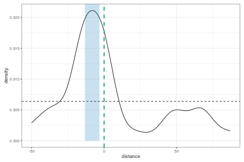
PA14_68920	NA	LysR family transcriptional regulator	DNA-binding transcription factor activity, regulation of transcription, DNA-templated		-27.18480	repression	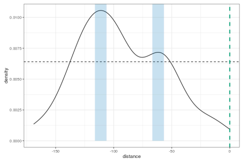
PA14_68955	NA	2-octaprenyl-3-methyl-6-methoxy-1,4-benzoquinol hydroxylase	ubiquinone biosynthetic process, oxidoreductase activity, acting on paired donors, with incorporation or reduction of molecular oxygen, NAD(P)H as one donor, and incorporation of one atom of oxygen, flavin adenine dinucleotide binding, oxidation-reduction process, FAD binding	Biosynthesis of secondary metabolites, Metabolic pathways, Ubiquinone and other terpenoid-quinone biosynthesis	-25.90195	repression	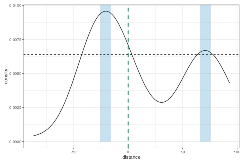
PA14_69090	NA	hypothetical protein			-26.29489	repression	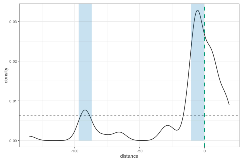
PA14_69140	NA	CDP-6-deoxy-delta-3,4-glucoseen reductase	electron transfer activity, iron-sulfur cluster binding, oxidoreductase activity, oxidation-reduction process	Amino sugar and nucleotide sugar metabolism	-26.24278	repression	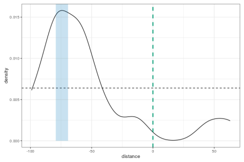
PA14_69170	NA	O-antigen acetylase	transferase activity, transferring acyl groups other than amino-acyl groups		-26.66883	repression	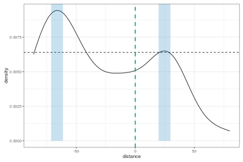
PA14_69230	ppk	polyphosphate kinase	polyphosphate biosynthetic process, polyphosphate kinase activity, polyphosphate kinase complex	Oxidative phosphorylation, Oxidative phosphorylation, RNA degradation	-26.01586	no impact	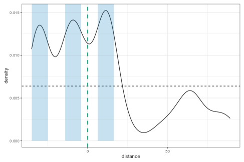
PA14_69320	NA	integral membrane transport protein	integral component of membrane		-25.84492	repression	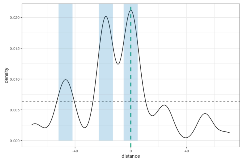

Modeled binding peaks for 1043 predicted targets of RsmA

PA14 gene ID	gene name	description	GO terms	KEGG pathways	overall affinity score in RT	predicted effect on translation	binding site predictions
PA14_69330	NA	hypothetical protein	membrane, transmembrane transport		-26.36819	repression	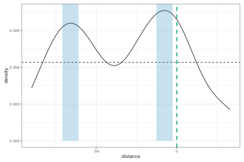
PA14_69390	algQ	anti-RNA polymerase sigma 70 factor	positive regulation of secondary metabolite biosynthetic process, negative regulation of proteolysis, positive regulation of single-species biofilm formation, regulation of transcription, DNA-templated		-26.26350	repression	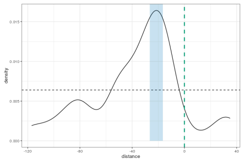
PA14_69620	NA	hypothetical protein			-25.91965	repression	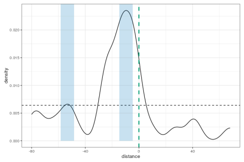
PA14_69670	lysA	diaminopimelate decarboxylase	catalytic activity, lysine biosynthetic process via diaminopimelate, diaminopimelate decarboxylase activity	Biosynthesis of amino acids, Biosynthesis of antibiotics, Biosynthesis of secondary metabolites, Lysine biosynthesis, Metabolic pathways, Microbial metabolism in diverse environments	-26.64274	repression	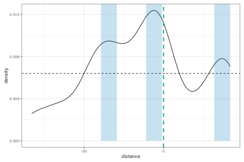
PA14_69850	NA	choline transporter	membrane, transmembrane transporter activity, nitrogen compound transport		-25.78194	repression	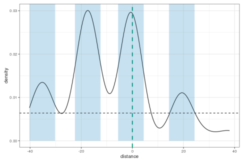
PA14_69870	pchP	phosphorylcholine phosphatase			-27.14901	repression	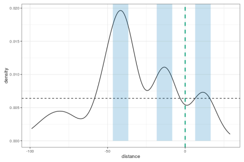
PA14_70060	NA	lipoprotein			-26.34428	repression	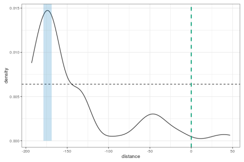
PA14_70070	NA	hypothetical protein			-26.82594	repression	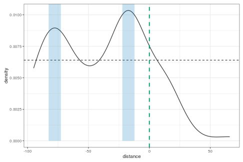
PA14_70190	rpmB	50S ribosomal protein L28	structural constituent of ribosome, ribosome, translation	Ribosome	-25.96308	no impact	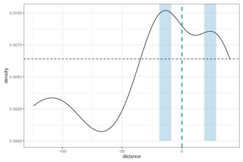
PA14_70230	radC	DNA repair protein RadC			-26.88931	repression	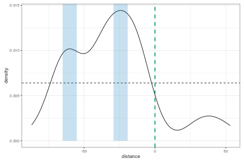

Modeled binding peaks for 1043 predicted targets of RsmA

PA14 gene ID	gene name	description	GO terms	KEGG pathways	overall affinity score in RT	predicted effect on translation	binding site predictions
PA14_70270	algC	phosphomannomutase	carbohydrate metabolic process, intramolecular transferase activity, phosphotransferases, magnesium ion binding, organic substance metabolic process, pathogenesis, phosphoglucomutase activity, lipopolysaccharide core region biosynthetic process, phosphomannomutase activity, alginic acid biosynthetic process	Amino sugar and nucleotide sugar metabolism, Amino sugar and nucleotide sugar metabolism, Biosynthesis of antibiotics, Biosynthesis of secondary metabolites, CMP-legionaminate biosynthesis I, Fructose and mannose metabolism, Galactose metabolism, Glycolysis / Gluconeogenesis, Metabolic pathways, Microbial metabolism in diverse environments, Pentose phosphate pathway, Purine metabolism, Starch and sucrose metabolism, Streptomycin biosynthesis	-26.44743	no impact	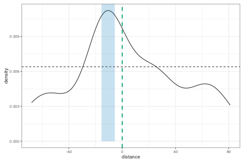
PA14_70360	NA	hypothetical protein			-26.70193	repression	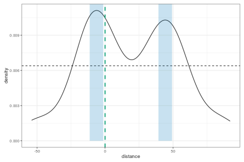
PA14_70470	spoT	guanosine-3',5'-bis(diphosphate) 3'-pyrophosphohydrolase	guanosine tetraphosphate metabolic process	Purine metabolism	-25.99657	repression	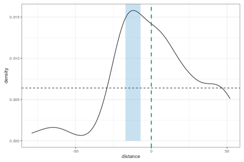
PA14_70490	NA	lipoprotein			-26.94896	no impact	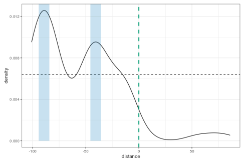
PA14_70600	NA	HU family DNA-binding protein	DNA binding		-26.12252	no impact	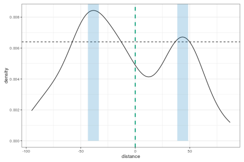
PA14_70650	NA	GlcG protein			-26.19697	repression	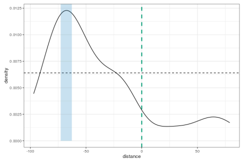
PA14_70760	phoR	two-component sensor PhoR	phosphorelay sensor kinase activity, phosphorelay signal transduction system, protein histidine kinase activity, integral component of membrane, regulation of transcription, DNA-templated, signal transduction, phosphorylation, transferase activity, transferring phosphorus-containing groups, phosphoprotein phosphatase activity	Two-component system	-26.07836	repression	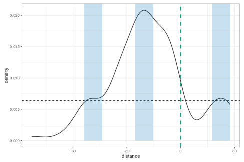
PA14_71060	sdaB	L-serine dehydratase	L-serine ammonia-lyase activity, gluconeogenesis, 4 iron, 4 sulfur cluster binding	Biosynthesis of amino acids, Biosynthesis of antibiotics, Biosynthesis of secondary metabolites, Carbon metabolism, Cysteine and methionine metabolism, Cysteine and methionine metabolism, Glycine, serine and threonine metabolism, Glycine, serine and threonine metabolism, Metabolic pathways	-25.78747	repression	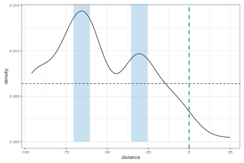
PA14_71160	NA	hypothetical protein	choline transport, choline binding, periplasmic space, transmembrane transporter activity, ATP-binding cassette (ABC) transporter complex, transmembrane transport	ABC transporters	-26.08623	repression	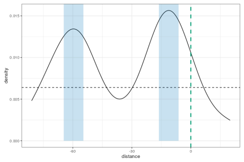
PA14_71250	NA	hypothetical protein	glycine betaine catabolic process, choline catabolic process		-26.07017	repression	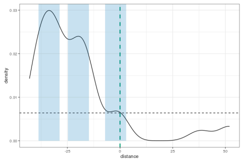

Modeled binding peaks for 1043 predicted targets of RsmA

PA14 gene ID	gene name	description	GO terms	KEGG pathways	overall affinity score in RT	predicted effect on translation	binding site predictions
PA14_71280	NA	ferredoxin	iron-sulfur cluster binding, choline catabolic process, glycine betaine catabolic process		-25.76588	repression	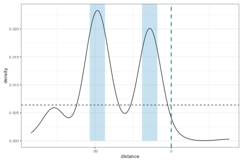
PA14_71390	NA	hypothetical protein			-26.50089	no impact	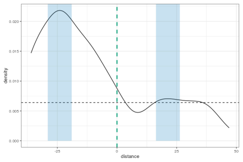
PA14_71410	NA	ring hydroxylating dioxygenase, alpha-subunit	oxidoreductase activity, 2 iron, 2 sulfur cluster binding, oxidation-reduction process, iron ion binding, cellular metabolic process		-26.05708	repression	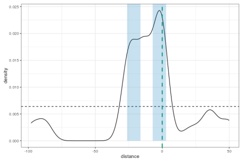
PA14_71430	NA	hypothetical protein			-26.43930	repression	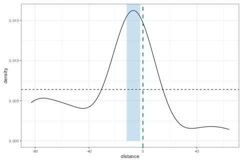
PA14_71460	glyA1	serine hydroxymethyltransferase	pyridoxal phosphate binding, glycine hydroxymethyltransferase activity, glycine biosynthetic process from serine, tetrahydrofolate interconversion, catalytic activity	Biosynthesis of amino acids, Biosynthesis of antibiotics, Biosynthesis of secondary metabolites, Carbon metabolism, Cyanoamino acid metabolism, Glycine, serine and threonine metabolism, Glyoxylate and dicarboxylate metabolism, Metabolic pathways, Methane metabolism, Microbial metabolism in diverse environments, One carbon pool by folate	-26.53998	no impact	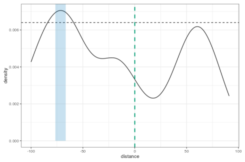
PA14_71490	soxD	sarcosine oxidase delta subunit	sarcosine oxidase activity, tetrahydrofolate metabolic process, sarcosine catabolic process	Glycine, serine and threonine metabolism, Metabolic pathways	-26.21340	repression	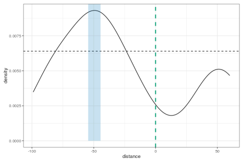
PA14_71510	soxG	sarcosine oxidase gamma subunit	protein binding, sarcosine oxidase activity, sarcosine catabolic process	Glycine, serine and threonine metabolism, Metabolic pathways	-26.72305	repression	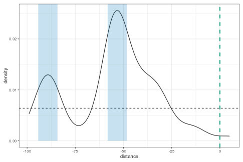
PA14_71590	NA	hypothetical protein	integral component of membrane		-26.73632	repression	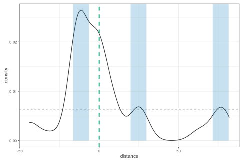
PA14_71620	purE	phosphoribosylaminoimidazole carboxylase catalytic subunit	'de novo' IMP biosynthetic process	Biosynthesis of antibiotics, Biosynthesis of secondary metabolites, Metabolic pathways, Purine metabolism	-25.92860	no impact	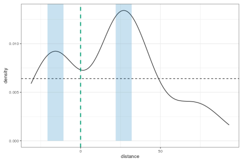
PA14_71630	adhA	alcohol dehydrogenase	oxidoreductase activity, zinc ion binding, oxidation-reduction process	Biosynthesis of antibiotics, Biosynthesis of secondary metabolites, Chloroalkane and chloroalkene degradation, Degradation of aromatic compounds, Fatty acid degradation, Glycolysis / Gluconeogenesis, Metabolic pathways, Microbial metabolism in diverse environments, Naphthalene degradation, Tyrosine metabolism	-25.94997	repression	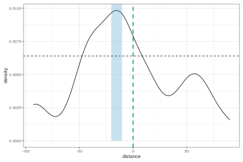

Modeled binding peaks for 1043 predicted targets of RsmA

PA14 gene ID	gene name	description	GO terms	KEGG pathways	overall affinity score in RT	predicted effect on translation	binding site predictions
PA14_71900	NA	hypothetical protein			-25.87426	repression	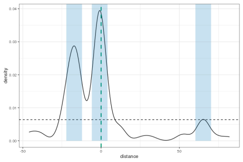
PA14_71990	gmd	GDP-mannose 4,6-dehydratase	GDP-mannose 4,6-dehydratase activity, GDP-mannose metabolic process	Amino sugar and nucleotide sugar metabolism, Fructose and mannose metabolism, Metabolic pathways	-26.43427	no impact	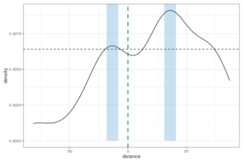
PA14_72010	NA	glycosyltransferase	lipopolysaccharide biosynthetic process		-26.07097	repression	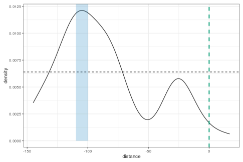
PA14_72210	NA	hypothetical protein	RNA processing, RNA ligase activity	beta-Lactam resistance	-25.81286	repression	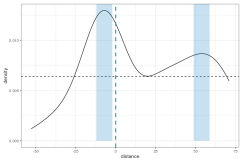
PA14_72340	gltP	glutamate/aspartate:proton symporter	symporter activity, integral component of membrane, dicarboxylic acid transport		-25.91665	repression	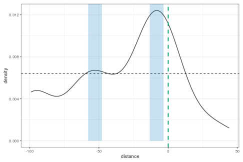
PA14_72370	NA	hypothetical protein	plasma membrane		-26.08570	repression	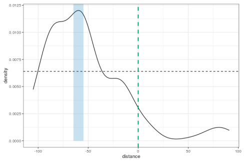
PA14_72380	algB	two-component response regulator AlgB	sequence-specific DNA binding, DNA binding, phosphorelay signal transduction system, ATP binding, regulation of transcription, DNA-templated, transcription factor binding, alginic acid biosynthetic process, positive regulation of transcription, DNA-templated, negative regulation of single-species biofilm formation on inanimate substrate, transcription regulatory region DNA binding, phosphorelay response regulator activity, phosphorelay sensor kinase activity, positive regulation of proteolysis, positive regulation of secondary metabolite biosynthetic process, positive regulation of cell motility	Two-component system	-27.21684	repression	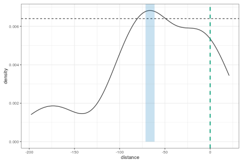
PA14_72520	NA	hypothetical protein			-25.89468	no impact	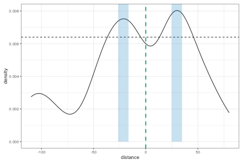
PA14_72580	znuC	zinc transporter	ATP binding, ATPase activity, plasma membrane, zinc ion transport, ATPase-coupled zinc transmembrane transporter activity, response to zinc ion	ABC transporters	-26.03917	repression	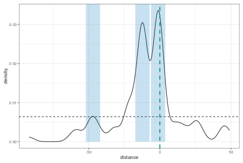
PA14_72740	NA	two-component sensor	phosphorelay sensor kinase activity, signal transduction, phosphorylation, transferase activity, transferring phosphorus-containing groups	Two-component system	-26.43810	no impact	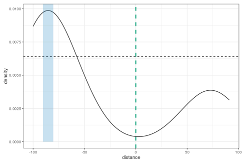

Modeled binding peaks for 1043 predicted targets of RsmA

PA14 gene ID	gene name	description	GO terms	KEGG pathways	overall affinity score in RT	predicted effect on translation	binding site predictions
PA14_72760	NA	beta-lactamase	penicillin binding, beta-lactam antibiotic catabolic process, beta-lactamase activity	beta-Lactam resistance	-26.58347	repression	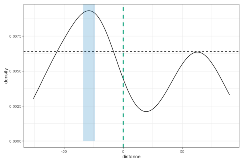
PA14_72770	NA	hypothetical protein			-26.09289	repression	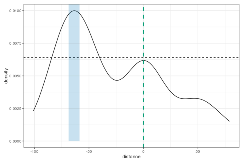
PA14_72810	NA	hypothetical protein			-25.93243	no impact	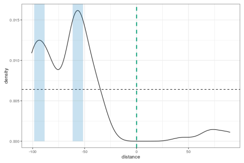
PA14_72960	NA	MFS dicarboxylate transporter	integral component of membrane, transmembrane transporter activity, transmembrane transport, integral component of plasma membrane		-25.83824	repression	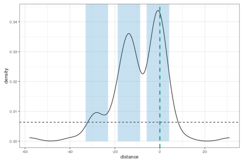
PA14_73150	NA	hypothetical protein			-26.92076	repression	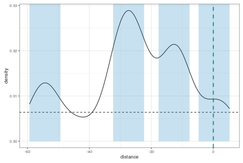
PA14_73200	NA	hypothetical protein			-27.04344	repression	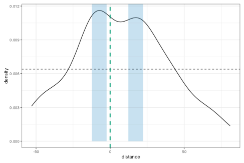
PA14_73350	soj	chromosome partitioning protein Soj			-26.12023	repression	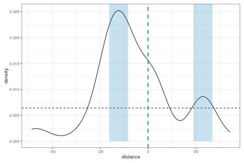
